# Sedimentary conditions drive modern pyrite burial flux to exceed oxidation

**DOI:** 10.1038/s41561-025-01855-5

**Published:** 2025-12-12

**Authors:** Cornelia Mertens, Sarah Paradis, Jordon D. Hemingway

**Affiliations:** 1 Geological Institute, Department of Earth and Planetary Sciences, ETH Zurich, Zurich, Switzerland; 2 Institute of Biogeochemistry and Pollutant Dynamics, Department of Environmental Systems Science, ETH Zurich, Zurich, Switzerland

**Keywords:** Element cycles, Element cycles

## Abstract

Pyrite (iron sulfide) formation and burial in sediments decreases atmospheric CO_2_ and increases O_2_ levels. However, the environmental and sedimentological conditions that regulate pyrite burial remain poorly constrained. Here we investigate such controlling mechanisms using a non-dimensional diagenetic model that extracts the natural variables governing pyrite formation rate and sulfur isotopic composition (*δ*^34^S). Both properties are controlled by the local ratios of organic carbon content to sulfate concentration and organic carbon reactivity to sedimentation rate; formation rate is additionally sensitive to reactive iron delivery. Using only globally interpolated boundary conditions, our model accurately predicts signals in 216 sediment cores distributed across the modern ocean. Extrapolating this, we estimate a global pyrite burial flux of 7.0 × 10^12^ mol S yr^−1^ (sensitivity test range: 2.5 × 10^12^ to 19.0 × 10^12^ mol S yr^−1^) with a weighted-average *δ*^34^S value of −4‰ (range: −8 to +3‰). This flux is substantially larger than terrestrial pyrite oxidation, indicating that the sulfur cycle is currently not in steady state but is instead described by net pyrite burial and thus atmospheric O_2_ accumulation. Finally, we interpret the geologic pyrite *δ*^34^S record within this model framework and identify flooded shelf area as the main control on pyrite burial throughout the Phanerozoic Eon.

## Abstract (web summary)

Modern pyrite burial in seafloor sediments exceeds pyrite oxidation on land, indicating that the sulfur cycle is out of balance, and acts to increase atmospheric oxygen levels, according to a diagenetic model validated by sediment cores.

## Main

The long-term evolution of Earth’s atmospheric composition is controlled by the interactive cycling of carbon, oxygen, sulfur and iron^1,2^. Of particular interest here, the sulfur cycle regulates atmospheric O_2_ and CO_2_ levels (pO_2_, pCO_2_) via two competing processes: (1) oxidative weathering on land of the iron sulfide mineral pyrite (FeS_2_), which directly consumes O_2_ and effectively releases CO_2_ by acidifying carbonate rocks^3^, and (2) microbial sulfate (${{\rm{SO}}}_{4}^{\!2-}$) reduction (MSR) in sediments^4^, which consumes organic carbon (OC) following the general reaction

1 $$1.7\,{\rm{OC}}+{{\rm{SO}}}_{4}^{\!2}\to {{\rm{S}}}^{-{\rm{II}}}+2\,{{\rm{HCO}}}_{3}^{\!-},$$

where S^−II^ represents the sum of sulfide species (that is, S^−2^, HS^−^ and H_2_S) and 1.7 is an empirically determined stoichiometric factor^5^. Product S^−II^ can subsequently react with reactive iron minerals (for example, ferrihydrite, goethite, haematite) to form pyrite, whereas ${{\rm{HCO}}}_{3}^{\!-}$ increases marine alkalinity and thus carbonate mineral formation^1,2^. By promoting both reduced sulfur and carbonate burial, MSR effectively increases pO_2_ and decreases pCO_2_, opposite to pyrite weathering. Thus, the balance between pyrite oxidation on land and pyrite burial in sediments determines the strength and magnitude of sulfur-cycle control on atmospheric composition^1,2,6^.

Pyrite burial is often reconstructed using paired sulfur isotope compositions (*δ*^34^S, reported in ‰ relative to the Vienna Canyon Diablo Troilite (VCDT) international standard; Methods) of pyrite and coeval sulfate-bearing minerals^7–11^. The utility of this approach lies in the fact that MSR preferentially reduces ${32\atop}{{\rm{SO}}}_{4}^{\!2-}$ relative to ${34\atop}{{\rm{SO}}}_{4}^{\!2-}$, which typically leads to pyrite exhibiting lower *δ*^34^S values than sulfate. If one assumes that (1) the sulfur cycle is in steady state on timescales shorter than that of marine sulfate turnover (that is, ~10^6^–10^7^ yr in the Phanerozoic Eon; 540 Ma to present)^12^, (2) riverine input fluxes and isotopic compositions are constant or can be constrained independently and (3) measured pyrite *δ*^34^S reflects global signals, then the paired isotopic offset, defined as

2 $${\Delta }_{{\rm{pyrite}}}={\delta }^{34}{{\rm{S}}}_{{{\rm{SO}}}_{4}}-{\delta }^{34}{{\rm{S}}}_{{{\rm{FeS}}}_{2}},$$

can be inverted to directly estimate pyrite burial flux through time^7,9–11^.

However, more recent work has shown that the sum of local—rather than global—environmental factors play a major role in setting *Δ*_pyrite_ values recorded at any given sampling location. For example, pyrite *δ*^34^S variability up to 50‰ has been observed on continental shelves over glacial–interglacial timescales (that is, ~10^5^ yr) (refs. ^13–16^). Because this is substantially shorter than the turnover time of marine sulfate, such variability must instead reflect local changes in sedimentation rate, OC content and reactivity, and/or reactive iron (Fe_HR_) delivery^17,18^. This interpretation questions the validity of traditional isotope mass-balance approaches for reconstructing pyrite burial flux. Despite these advancements, a detailed and quantitative examination of these drivers across environmentally relevant conditions remains lacking, precluding robust and independent estimates of pyrite burial flux and its relationship to preserved *Δ*_pyrite_ values. This knowledge gap hinders our ability to predict and interpret how the sulfur cycle drives and responds to Earth-system perturbations, both today and in the geologic past.

## Conceptual framework

To address this, here we build a non-dimensional advection–diffusion–reaction diagenetic model to quantify the formation rate and isotopic composition of pyrite buried in marine sediments globally (assumptions and derivation details in Methods and Supplementary Discussion, sections 1–3). This work builds upon two recent observations: (1) MSR exhibits slow reduction rates near the thermodynamic limit—and thus expresses equilibrium isotope fractionation factors—in nearly all natural settings^4,17,19–23^ and (2) preserved pyrite isotopic compositions change with local sedimentological conditions^13–15,18^. Using this understanding, we identify two key non-dimensional variables that govern pyrite isotopic composition (all variables defined in Extended Data Tables 1 and 2). First is the OC-to-sulfate ratio, expressed as

3 $${\Gamma }_{0}=\frac{{f}_\mathrm{G}{G}_{0}}{{S}_{0}},$$

where *f*_G_ is a factor that converts wt% OC respired to mM ${{\rm{SO}}}_{4}^{2-}$ reduced, *G*_0_ is the OC content at the top of the sulfidic anoxic zone^24,25^ and *S*_0_ is the marine sulfate concentration. For the modern ocean with 28 mM sulfate^7^, typical sedimentary OC contents of ~0.2 to 1.4 wt% (refs. ^24,25^) and porosities of ~0.6 to 0.9 (ref. ^26^) translate to *Γ*_0_ values of ~1 to 40. Second is a modified Damköhler number, defined as

4 $${{\rm{Da}}}^{* }=\frac{\sqrt{{D}_\mathrm{S}{k}_{\mathrm{G}_{0}}}}{w},$$

where *D*_S_ is sulfate diffusivity^27^, ${k}_{\mathrm{G}_{0}}$ is OC respiration-rate coefficient at the top of the sulfidic anoxic zone and *w* is sedimentation rate^28^. Broadly, *Γ*_0_ describes the extent and rate at which MSR progresses, whereas Da^*^ quantifies the relative importance of sedimentation, diffusion and reaction in supplying or removing sulfate from porewaters. Pyrite burial flux is additionally sensitive to Fe_HR_ delivery, defined as

5 $${\Psi }_{0}=\frac{{f}_\mathrm{F}{F}_{0}}{{S}_{0}},$$

where *f*_F_ is a factor that converts wt % Fe_HR_ to mM S^−II^ in porewater and *F*_0_ is the Fe_HR_ content at the top of the sulfidic anoxic zone^25,29^. For the modern ocean, typical sedimentary Fe_HR_ contents of ~0.3 to 3.0 wt% translate to *Ψ*_0_ values of ~1 to 50, similar to *Γ*_0_. Equations (3)–(5) define the natural variables controlling pyrite formation.

Our model solves for non-dimensional concentrations/contents and *δ*^34^S values of sedimentary pyrite, porewater sulfate and porewater sulfide as functions of *Γ*_0_, Da^*^ and *Ψ*_0_. To accurately describe the well-known observation that OC reactivity declines as bioavailable components are progressively degraded, we allow decay-rate coefficients to decrease with time following a power law^30^. We similarly conceptualize reactive iron reduction^29^ by (1) treating Fe_HR_ as a continuum of iron(hydr)oxide species with varying reactivities towards sulfide; (2) deriving, testing and validating a power-law relationship for Fe_HR_ decay; and (3) extracting a time-dependent decay-rate coefficient from global down-core Fe_HR_ trends (Supplementary Table 1 and Supplementary Figs. 1 and 2). Following common practice^8^, we assume sulfate reduction and pyritization follow Monod-like kinetics, and we constrain Monod coefficients using available experimental pyritization rate data (Supplementary Fig. 3). Finally, we assume all decayed Fe_HR_ contributes to pyrite formation when sulfide is present and that MSR always expresses a temperature-dependent equilibrium sulfur isotope fractionation factor^17,19,31^.

After constraining these governing relationships, the only required inputs are local sedimentological boundary conditions at the onset of the sulfidic anoxic zone, which allow us to calculate *Γ*_0_, Da^*^ and *Ψ*_0_ (compilation details in Methods, Extended Data Table 3, Supplementary Discussion, sections 4 and 5, and Supplementary Figs. 4–21). Global dynamics—for example, changes in sea-level or riverine sulfate delivery—impact our model only insofar as they modify these boundary conditions. Our approach requires no free fitting parameters or OC/FeS_2_ scaling factors (compare with refs. ^1,2,7,17,32^) and is independent of assumptions/parameterizations regarding sulfur-cycle steady state, sulfate in-/output fluxes and isotopic compositions (compare with refs. ^6,7,11,33^). In what follows, we (1) validate our model using globally distributed sedimentary profiles of OC, Fe_HR_ and all relevant sulfur species; (2) predict global burial flux and *Δ*_pyrite_ in the modern ocean and (3) interpret the Phanerozoic *Δ*_pyrite_ record to reconstruct how pyrite burial drivers have changed through time.

## Model validation in the modern ocean

**Fig. 1:**
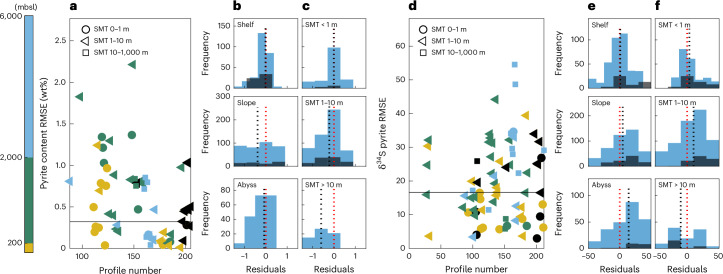
Assessing model performance across diverse bathymetric regions. **a**,**d**, Model–data misfit RMSE from 216 globally distributed sediment profiles for pyrite content (**a**) and *δ*^34^S value (**d**) as functions of (arbitrary) profile number. **b**,**c**,**e**,**f**, Also shown are histograms of model–data misfit residuals as functions of water depth^49^ (**b**,**e**) and sulfate–methane transition (SMT) depth^50^ (**c**,**f**). Marker colours in **a** and **d** refer to profile water depth, whereas shapes refer to SMT depth. Euxinic Black Sea profiles are shown as black markers in **a** and **d** and as black bars in **b**,**c** and **e**,**f**. Median RMSE values across all profiles are shown as horizontal black lines (0.32 wt% for pyrite content, 16.6‰ for pyrite *δ*^34^S). Residual medians (vertical dotted black lines) are consistently near zero (vertical dotted red lines) for all bathymetric regions.

**Extended Data Fig. 1:**
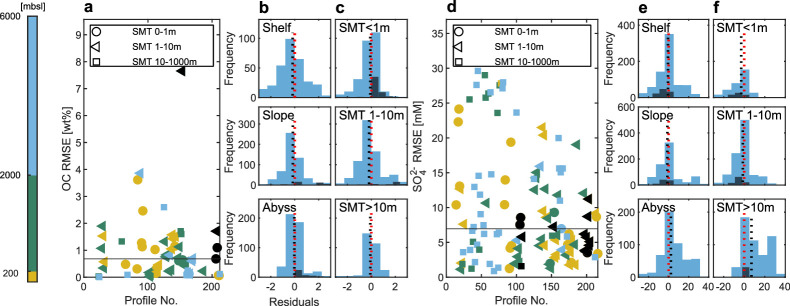
Assessing OC and sulfate concentration model performance across diverse bathymetric regions. Model–data misfit root-mean square error (RMSE) for (**a**) OC content and (**d**) sulfate concentration as functions of (arbitrary) profile number. Histograms showing the distributions of model–data misfit residuals for OC and sulfate concentrations as functions of (**b**, **c**) water depth and (**e**, **f**) sulfate-methane transition (SMT) depth. Marker colours in panels (**a**) and (**d**) refer to profile water depth, whereas marker shapes refer to SMT depth. Additionally, to assess the importance of bottom-water euxinia, Black Sea profiles are shown as black markers in panels (**a**) and (**d**) and as black bars in panels (**b**-**c**) and (**e**-**f**). Median RMSE values across all profiles are shown as horizontal black lines and are 0.66 wt% for OC content and 7.0 mM for sulfate concentration. Median residual values (vertical dotted black lines) are generally close to zero (vertical dotted red lines) for all bathymetric regions, indicating the robustness of our model fits.

**Extended Data Fig. 2:**
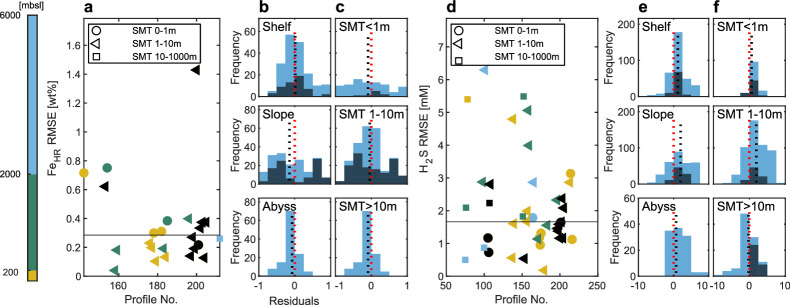
Assessing Fe_HR_ and sulfide concentration model performance across diverse bathymetric regions. Model–data misfit root-mean square error (RMSE) for (**a**) Fe_HR_ content and (**d**) sulfide concentration as functions of (arbitrary) profile number. Histograms showing the distributions of model–data misfit residuals for Fe_HR_ and sulfide concentrations as functions of (**b**, **c**) water depth and (**e**, **f**) sulfate-methane transition (SMT) depth. Marker colours in panels (**a**) and (**d**) refer to profile water depth, whereas marker shapes refer to SMT depth. Additionally, to assess the importance of bottom-water euxinia, Black Sea profiles are shown as black markers in panels (**a**) and (**d**) and as black bars in panels (**b**-**c**) and (**e**-**f**). Median RMSE values across all profiles are shown as horizontal black lines and are 0.28 wt% for Fe_HR_ content and 1.67 mM for sulfide concentration. Median residual values (vertical dotted black lines) are generally close to zero (vertical dotted red lines) for all bathymetric regions, indicating the robustness of our model fits.

**Extended Data Fig. 3:**
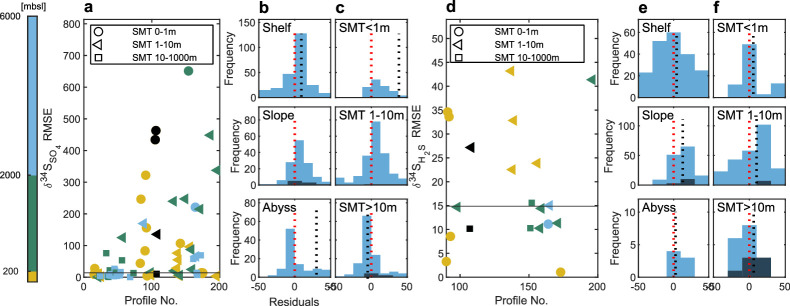
Assessing sulfate and sulfide isotopic composition model performance across diverse bathymetric regions. Model–data misfit root-mean square error (RMSE) for (**a**) sulfate and (**d**) sulfide *δ*^34^S values as functions of (arbitrary) profile number. Histograms showing the distributions of model–data misfit residuals for sulfate and sulfide *δ*^34^S values as functions of (**b**, **c**) water depth and (**e**, **f**) sulfate-methane transition (SMT) depth. Marker colours in panels (**a**) and (**d**) refer to profile water depth, whereas marker shapes refer to SMT depth. Additionally, to assess the importance of bottom-water euxinia, Black Sea profiles are shown as black markers in panels (**a**) and (**d**) and as black bars in panels (**b**-**c**) and (**e**-**f**). Median RMSE values across all profiles are shown as horizontal black lines and are 14.2 ‰ for sulfate and 14.9 ‰ for sulfide *δ*^34^S values. Median residual values (vertical dotted black lines) are generally close to zero (vertical dotted red lines) for all bathymetric regions, indicating the robustness of our model fits. Note that high sulfate *δ*^34^S RMSE (that is, approaching ~ 700 ‰) results from large model–data misfit at depth when sulfate concentrations approach zero (that is, highly distilled by Rayleigh fractionation). Nevertheless, pyrite is not actively precipitating at these depths; thus, large RMSE in these regions exhibits minimal impact on resulting sulfide and pyrite isotopic compositions (see Supplementary Discussion, section 7 for details).

First we confirm solver stability over the range of conditions considered here using the method of manufactured solutions^34^ (Supplementary Discussion, section 6, and Supplementary Figs. 22–25). Next we validate our model by predicting down-core sulfur species content/concentration and *δ*^34^S profiles and comparing to measured values from 216 globally distributed sediment cores representing a range of natural oceanographic settings (Supplementary Discussion, section 7, Supplementary Table 2 and Supplementary Figs. 26 and 27). Finally, we quantify accuracy and precision using model–data misfit root-mean square error (RMSE) and residual distributions. Our model performs equally well across three bathymetric regimes—continental shelves (0–200 metres below sea level, mbsl), continental slopes (200–2,000 mbsl) and the abyssal ocean (≥ 2,000 mbsl)—and all sulfate–methane transition zone depths (Fig. 1 and Extended Data Figs. 1–3). For all settings, results are unbiased with reasonably high accuracy and precision despite several simplifying assumptions.

Across our entire dataset, resulting RMSE exhibits median values of 0.3 wt% for pyrite content and 16‰ for pyrite *δ*^34^S (Fig. 1); the latter mainly reflects measured data scatter, for example, due to short-term variability in sedimentological conditions^35^. Although we are aware of no previous studies to compare pyrite model performance, our sulfate concentration RMSE values (median = 7.0 mM; Extended Data Fig. 1) are only slightly higher than those of ref. ^36^ despite their model being tuned to each individual profile. Combined with residuals that approach normal distributions with median values always near zero (Fig. 1 and Extended Data Figs. 1–3), this suggests that our approach does not add significant bias or error beyond that of natural scatter. We therefore treat this RMSE as representing natural uncertainty and we solve our model at each point in the ocean using gridded boundary condition maps to estimate global pyrite burial flux and isotopic composition.

## Modern pyrite burial signals

**Fig. 2:**
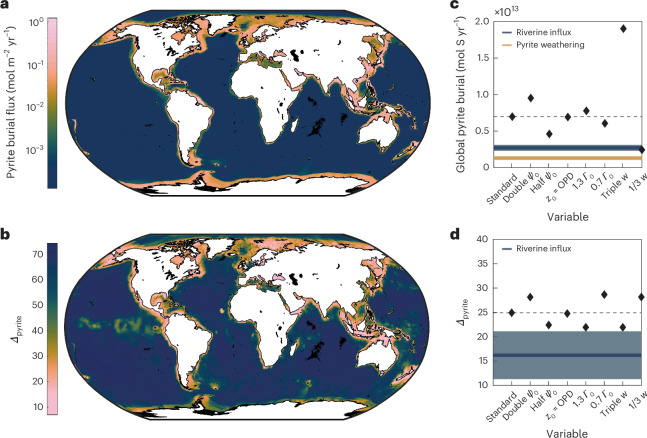
Predicted global signals and sensitivity test results in the modern ocean. **a**,**b**, Pyrite burial flux (**a**) and isotopic composition (**b**), reported as *Δ*_pyrite_ (equation (2)). Black areas represent regions of no pyrite formation due to oxygen penetration to basement^51^. **c**,**d**, Also shown are sensitivity test results highlighting the most important controlling variables for total global pyrite burial flux (**c**) and isotopic composition (**d**) (complete results in Extended Data Fig. 4). In both panels, dashed horizontal black line represents our ‘standard’ model result, whereas blue horizontal line and shading represents the estimated flux and isotopic composition of sulfate delivered to the oceans by rivers (*μ* ± 1*σ*; ref. ^3^). Panel **c** additionally shows the estimated flux of pyrite-weathering-derived riverine sulfate^3^. Variables refer to: *Ψ*_0_ = Fe_HR_ delivery, OPD = oxygen-penetration depth, *Γ*_0_ = OC delivery and *w* = sedimentation rate. Basemaps in **a** and **b** created with the MATLAB Mapping Toolbox with data from the US National Geospatial-Intelligence Agency under a GNU Lesser General Public License.

**Extended Data Fig. 4:**
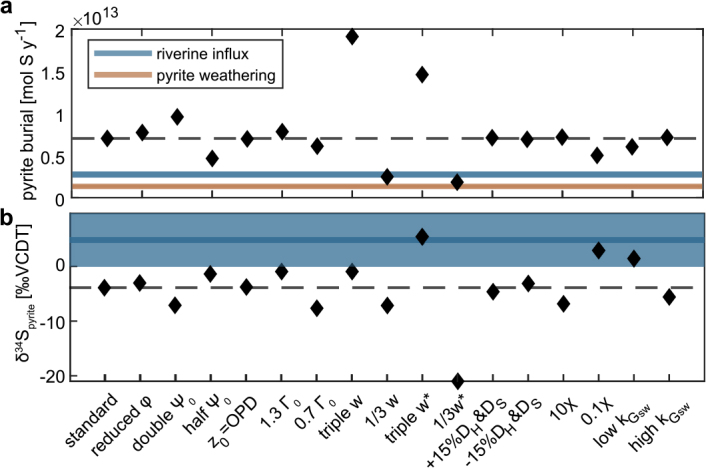
Sensitivity test results. Model-predicted global pyrite (**a**) burial flux and (**b**) weighted-average isotopic composition for various sensitivity test conditions. In all cases, we re-solved our global model after independently adjusting each boundary condition as stated in the x-axis labels. For all tests except for sedimentation rate, all boundary conditions other than the boundary condition of interest were held constant. For sedimentation rate, w refers to the solutions where all sedimentation-rate-dependent boundary conditions were also adjusted accordingly; in contrast, w? refers to the solutions in which sedimentation rate was adjusted but all dependent boundary conditions were held constant. For a given test, the exact value by which we increase/decrease each boundary condition is chosen based on observed uncertainty in that boundary condition (see Supplementary Discussion, section 4 for quantification of these values). In both panels, dashed horizontal black line represents the value for our ‘standard’ model conditions, whereas blue horizontal line and shading represents the estimated flux and isotopic composition of sulfate delivered to the oceans by rivers (*μ* ± 1*σ*; Ref. ^3^). Panel (**a**) additionally shows the estimated flux of pyrite-weathering derived riverine sulfate, which represents a subset of the total riverine delivery. With the exception of low w and low w? tests, pyrite burial flux always exceeds that of oxidative pyrite weathering on land. Similarly, buried pyrite is depleted in ^34^S relative to riverine inputs for all but low w and low w^*^ test results.

We first compile globally gridded environmental and sedimentological boundary conditions at ≤ 25 arcminute resolution. Next we use these parameters to generate maps of the non-dimensional variables *Γ*_0_, Da^*^ and *Ψ*_0_. Finally, to predict pyrite content, burial flux and *Δ*_pyrite_ globally, we solve our model at each grid point in our non-dimensional variable maps (Fig. 2a,b). Because globally gridded boundary conditions are not known perfectly, we perform a sensitivity test that propagates input uncertainty to predicted pyrite burial flux and isotopic composition (details in Methods and Supplementary Discussion, section 8). Our model predicts that *Δ*_pyrite_ values are most sensitive to sedimentation-rate uncertainty^28^; burial fluxes are additionally sensitive to Fe_HR_ content uncertainty, which remains high owing to sparse data^25^ (Fig. 2c,d, Extended Data Fig. 4 and Supplementary Figs. 28–31).

**Extended Data Fig. 5:**
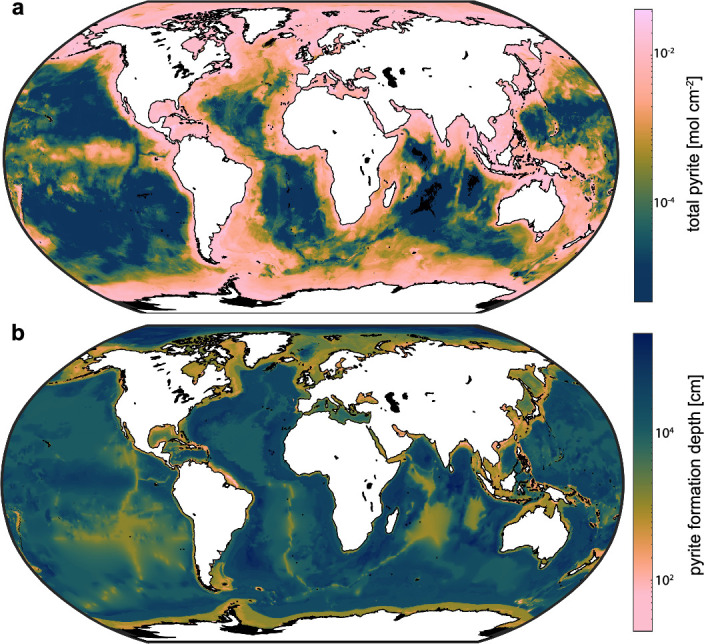
Global pyrite content and formation depth. Model-predicted (**a**) pyrite content within the sulfidic anoxic zone and (**b**) maximum depth of formation, ${\zeta }_{\max }$. For both panels, we use a pyrite formation threshold of *β* = 5 × 10^−4^ (see Supplementary Fig. 28 for choice of *β* value); both panels have additionally been re-cast into dimensional units (Eqs. S114 and S138) for ease of interpretation. Pyrite formation depth can become large in sediments characterized by steep pyrite concentration profiles such as deep-sea environments. However, total pyrite content in these settings still remains low due to slow pyrite formation rates (c.f., blue-green regions in both panels). Although absolute values depend on our choice of *β*, relative changes are less sensitive to this choice. Black areas represent regions with no pyrite formation due to oxygen penetration to basement (that is, z_OPD_ > z_tot_). Basemaps created with the MATLAB Mapping Toolbox with data from the US National Geospatial-Intelligence Agency under a GNU Lesser General Public License.

We predict that 7.0 × 10^12^ mol S yr^−1^ are buried as pyrite (sensitivity test range: 2.5 × 10^12^ to 1.9 × 10^13^ mol S yr^−1^; Fig. 2c), with 48% occurring on continental shelves, 31% on slopes and only 21% in the abyss despite comprising ≈ 85% of total ocean area (Extended Data Fig. 5 and Supplementary Fig. 32). To our knowledge, this represents the first independent pyrite burial flux estimate that does not rely on OC burial scaling factors^32^, global mass-balance box models^37^ or the assumption of sulfur-cycle steady state^3,6^. Still, previous work^32^ has estimated modern pyrite burial fluxes of ~1 × 10^12^ to 2 × 10^12^ mol S yr^−1^, which is ~3.5× to 7× lower than our estimate (range: ~1.3× to 19× lower). We therefore conclude that modern pyrite burial flux is significantly higher than previously assumed.

Recent studies estimate that rivers discharge 2.8 ± 0.3 × 10^12^ mol S yr^−1^ as sulfate, with ~50% derived from pyrite weathering^3^. Because rivers dominate sulfate inputs^38^ and pyrite burial dominates sulfate outputs from the modern ocean^39^, we take the difference in these fluxes to estimate a net loss of ~4.2 × 10^12^ mol S yr^−1^ due to excess pyrite burial (range: ~0 to 1.7 × 10^13^ mol S yr^−1^). Any additional ‘cryptic’ gypsum or anhydrite precipitation (for example, during dolomitization in carbonate platforms^40^) would further increase net sulfur loss and exacerbate departure from steady state. This finding contrasts with a reconstructed sulfate concentration increase across the Cenozoic Era (66 Ma to present) based on evaporite fluid inclusions^12^, suggesting that modern (interglacial) conditions do not represent the long-term average. Indeed, exposure of continental shelves during (glacial) sea-level lowstands would substantially decrease pyrite burial flux and increase terrestrial oxidation relative to today^41,42^. Importantly, our model boundary conditions reflect present-day conditions; any conclusions about steady state (or lack thereof) apply only to the modern system and not to other periods in Earth’s history when sedimentation rate, OC and Fe_HR_ delivery, and sulfur-cycle dynamics may have been considerably different.

Still, by comparing pyrite burial and oxidation fluxes^3^, we estimate the impact of sulfur cycling on pO_2_. Because 15 mol e^−^ are transferred per mol FeS_2_ oxidized or buried, we predict the modern sulfur cycle promotes the release of 1.1 × 10^13^ mol O_2_ yr^−1^ (range: 1.9 × 10^12^ to 3.3 × 10^13^ mol O_2_ yr^−1^). This estimate is similar to that of O_2_ released by net OC burial in sediments relative to oxidative weathering of rock-derived OC on land (excluding anthropogenic emissions): assuming 4 mol e^−^ per mol OC oxidized or buried, OC cycling promotes the release of 4.3 × 10^12^ mol O_2_ yr^−1^ (uncertainty range: 2.8 × 10^12^ to 7.8 × 10^12^ mol O_2_ yr^−1^) (refs. ^32,43^). These two processes combined should increase pO_2_ by ~1% (relative) per 100 kyr (uncertainty/sensitivity range: 0.3% to 2.5% per 100 kyr). However, ice core data suggest that pO_2_ has decreased by 0.7% (relative) since 800 ka (ref. ^44^). This indicates: (1) the modern sulfur and/or OC cycles do not represent long-term averages and/or (2) there exists a major ‘missing’ O_2_ sink. Regardless of long-term variability, both elemental cycles today very likely promote O_2_ accumulation, even when considering relatively large flux-balance uncertainty.

We additionally estimate a modern burial flux-weighted-average pyrite *δ*^34^S value of −4‰ VCDT (range: −8 to 3‰ VCDT; Fig. 2d and Extended Data Fig. 4b), identical to that reported recently using an independent modelling approach^17^. This corresponds to a *Δ*_pyrite_ value of 25‰ (range: 18 to 29‰), significantly smaller than the equilibrium microbial isotope effect of ~65 to 70‰ (refs. ^21–23,31^). The departure of *Δ*_pyrite_ from the microbial isotope effect reflects the dominance of pyrite formation on shelf environments, where high sedimentation rates, OC and Fe_HR_ delivery lead to ‘closed-system’ dynamics that dampen the expression of MSR fractionation^17^.

## Interpreting the Phanerozoic *Δ*_pyrite_ record

**Fig. 3:**
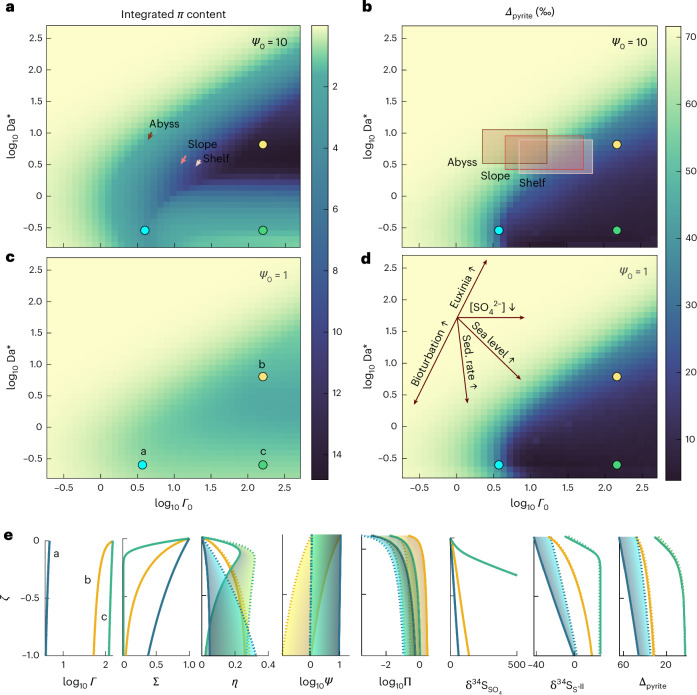
Sulfur-cycle parameter-space heat maps. **a**–**d**, Integrated non-dimensional pyrite content (**a**,**c**) and isotopic composition (**b**,**d**) exported from the sulfidic anoxic zone as functions of *Γ*_0_ and Da^*^. Panels **a** and **b** were calculated using the global averaged Fe_HR_ content, *Ψ*_0_ = 10, whereas panels **c** and **d** were calculating using *Ψ*_0_ = 1 as a low-iron endmember scenario. **e**, Example profiles showing predicted non-dimensional species contents/concentrations (OC, *Γ*; sulfate, *Σ*; sulfide, *η*; Fe_HR_, *Ψ*; and pyrite, *Π*) and isotopic compositions (${\delta }^{34}{{\rm{S}}}_{{{\rm{SO}}}_{4}}$, ${\delta }^{34}{{\rm{S}}}_{{{\rm{S}}}^{-{\rm{II}}}}$ and *Δ*_pyrite_) as functions of non-dimensional depth, *ζ*, for three contrasting parameter-space conditions (marked as coloured circles in **a**–**d**). Stippled lines and lighter colours represent low *Ψ*_0_ whereas solid lines and darker colours represent high *Ψ*_0_ conditions. Vectors in panel **a** represent the expected change in global-average *Γ*_0_ and Da^*^ for each bathymetric region when using the oxygen-penetration depth (vector base) vs the mixed-layer depth (vector tip) as model input. Boxes in panel **b** represent the 5th to 95th percentile range of *Γ*_0_ vs Da^*^ extent observed for each bathymetric region in the modern ocean. Vectors in panel **d** represent global changes to various Earth-system evolution processes discussed in the main text. Sed. rate, sedimentation rate.

To interpret the geologic record, we generate parameter-space heat maps and visualize how Earth-system perturbations to environmental and sedimentological conditions can influence non-dimensional governing variables and thus sedimentary pyrite content and isotopic composition. Within this framework, each physical and biological process/innovation can be interpreted as unique vectors in *Γ*_0_ vs Da^*^ space (Fig. 3). For example, changing marine sulfate concentration represents a horizontal line, as this influences *Γ*_0_ but not Da^*^; depending on the starting conditions and magnitude of change, such a shift may or may not impact *Δ*_pyrite_ and *Π*, the non-dimensional sedimentary pyrite content (red arrows in Fig. 3d). Visualizing the sulfur cycle in this way yields three important insights.

First, *Δ*_pyrite_ and *Π* are largely insensitive to bioturbation depth, which was previously proposed as a primary driver of pyrite *δ*^34^S across the Phanerozoic^7^. We show instead that by increasing the amount of OC respired aerobically, deeper bioturbation simultaneously decreases OC content and reactivity, thus decreasing both *Γ*_0_ and Da^*^ along a line of (near) constant *Δ*_pyrite_ and *Π*. To validate this, we calculate how modern signals shift if one assumes net MSR begins at the oxygen-penetration depth vs at the mixed-layer depth, which can approach 10× deeper than the oxygen-penetration depth^45^. For all bathymetric settings, this choice negligibly impacts *Δ*_pyrite_ and *Π* despite changing *Γ*_0_ and Da^*^ (arrows in Fig. 3a). A corollary states that *Δ*_pyrite_ and *Π* are also insensitive to bottom-water euxinia. By decreasing aerobic respiration, euxinia increases both OC content and reactivity and thus moves along the same vector as bioturbation, although opposite in sign. The fact that our model predicts Black Sea profiles—with bottom-water S^−II^ concentrations of ≤ 400 μM (ref. ^46^)—equally well as open-ocean settings further supports this claim. Nevertheless, substantially higher bottom-water S^−II^ concentrations may still have exerted a large influence on pyrite burial flux and isotopic composition in Precambrian oceans (Supplementary Fig. 33).

Second, while *Π* is highly sensitive to Fe_HR_ delivery, *Δ*_pyrite_ is not (Fig. 2c,d). Because ^34^S fractionation during the conversion of sulfide to pyrite is negligible, any impact of Fe_HR_ delivery on pyrite *δ*^34^S must occur via a shift in the locus of pyrite formation depth—and thus of substrate sulfide ^34^S composition—within the sediment column. However, sulfide profiles exhibit relatively shallow *δ*^34^S gradients under typical environmental conditions due to the importance of diffusion (Fig. 3e), thus minimizing this effect. By contrast, sulfide is effectively sequestered as pyrite at sites with exceedingly high Fe_HR_ delivery (that is, ‘closed-system’ dynamics). This leads to the direct incorporation of instantaneously formed sulfide at the top of the sediment column—which is ^34^S-depleted—into pyrite without being impacted by diffusion of isotopically heavier sulfide from below. Although such settings appear to be rare in the modern ocean, marine-sediment reactive iron measurements are sparse^26^ and Fe_HR_ remains relatively underconstrained.

Third, both *Δ*_pyrite_ and *Π* are highly sensitive to changes in sedimentation rate and, by extension, global sea level (Fig. 2c,d and 3d). Increasing sea level increases the area of flooded continental shelf^47^ and thus globally averaged sedimentation rate and OC delivery; this decreases Da^*^ and increases *Γ*_0_. Unlike bioturbation, the sea-level vector pushes the system (nearly) perpendicular to lines of constant *Δ*_pyrite_ and *Π*. This is supported by measured signals in several shelf settings^13–16^. For example, observed sedimentation-rate and OC content variability in a New Zealand site studied by ref. ^14^ leads to a glacial–interglacial change in *Γ*_0_ from 3.9 to 25.2 and in Da^*^ from 3.7 to 11.4; this corresponds to a model-predicted shift in *Δ*_pyrite_ from 21 to 66‰, similar to measured values (3 to 68‰). Such model–data agreement supports the conclusion that our model effectively reconstructs observed pyrite isotopic variations driven by sea-level and sedimentation-rate changes. Thus, whereas *Δ*_pyrite_ sensitivity to flooded shelf area has been suggested previously^8,17,33^, here we provide the first testable framework to quantify its impact, including on pyrite content in addition to isotopic composition.

**Fig. 4:**
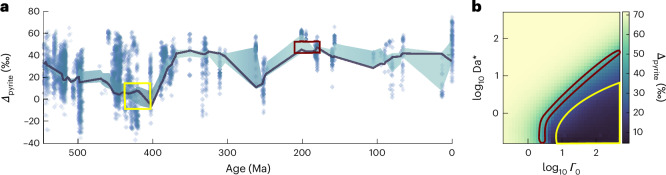
Sedimentological conditions as predicted by the *Δ*_pyrite_ record. **a**, Compilation of *Δ*_pyrite_ across the Phanerozoic Eon (541 million years ago (Ma) to present) showing individual data points (blue circles), smoothed average (thick blue line) and bootstrapped 5th to 95th percentiles of resampled data (shaded blue region; Methods). **b**, Heat map of *Δ*_pyrite_ as a function of *Γ*_0_ (the OC-to-sulfate ratio) vs Da^*^ (the ratio of sulfate diffusivity, OC reactivity and sedimentation rate; assuming *Ψ*_0_ = 10; copied from Fig. 3b). Coloured boxes in panel **a** highlight two periods discussed in the main text: the upper Silurian to Early Devonian (yellow) and the Late Triassic to Early Jurassic (red). Corresponding coloured regions in panel **b** represent the permissible *Γ*_0_ and Da^*^ ranges for these two time periods, as determined by their *Δ*_pyrite_ values (using bootstrapped 5th to 95th percentile ranges).

We apply this framework to interpret the Earth-history *Δ*_pyrite_ record (Fig. 4 and Methods). Because our assumption of MSR equilibrium isotope fractionation is only valid when sulfate concentrations exceed ~3 mM (ref. ^17^), we limit our analysis to the Phanerozoic Eon, when this criterion was continuously met^12^. We exemplify our approach by comparing two periods: (1) the upper Silurian to Early Devonian, when *Δ*_pyrite_ averages ~0 to 10‰ (~430 to 393 Ma; yellow outline in Fig. 4) and (2) the latest Triassic to Early Jurassic, when *Δ*_pyrite_ averages ≥ 40‰ (~215 to 175 Ma; red outline in Fig. 4). Using these *Δ*_pyrite_ ranges as constraints, we predict the upper Silurian to Early Devonian was described by high *Γ*_0_ and low Da^*^, whereas the latest Triassic to Early Jurassic occupied a narrow diagonal band at intermediate *Γ*_0_ and Da^*^ (Fig. 4b). These results agree with Phanerozoic sea-level^47^ and sulfate concentration^12^ estimates: whereas sulfate concentrations were relatively similar in both periods (~5–15 mM), the upper Silurian to Early Devonian contained the largest area of flooded continental shelf at any point in the Phanerozoic. This drove elevated sedimentation rates and OC delivery, leading to high *Γ*_0_ and low Da^*^. By contrast, the latest Triassic to Early Jurassic was a period with substantially less flooded shelf area and thus relatively low sedimentation rate, OC delivery and *Γ*_0_ and high Da^*^.

We use these constraints to predict buried pyrite content at these times. Although Fe_HR_ delivery remains unknown, this was probably greater than modern values because flooded shelf area today is lower than in both periods; *Π* predictions described here thus represent minimum estimates. Nevertheless, permissible *Γ*_0_ and Da^*^ ranges in the latest Triassic to Early Jurassic predict homogeneous, intermediate *Π* values, whereas those permissible in the upper Silurian to Early Devonian predict *Π* values ranging from considerably higher (if Da^*^ ≳ 1) to considerably lower (if Da^*^ ≪ 1) than today. Because of its coupled influence on OC delivery and sedimentation rate, we thus identify flooded shelf area as the primary variable governing the marine sulfur cycle over the Phanerozoic^48^. Our approach provides the first quantitative framework to test and validate how various Earth-system processes have impacted pyrite isotopic compositions, burial fluxes and atmospheric composition throughout Earth’s history.

## Methods

Here we first briefly summarize our model approach, governing equations, implementation and sensitivity tests; we then report Earth-history *Δ*_pyrite_ data compilation methods. All details, including justification of model assumptions, model derivation, boundary condition derivation and compilation, MATLAB solver verification, model performance assessment and model interpretation and sensitivity tests are discussed in Supplementary Discussion, sections 1–8. All modern validation sedimentary profile input data, globally gridded modern model output data and MATLAB code used to generate all results are included in Supplementary Data and Supplementary Information, software.

### General considerations and model assumptions

To model pyrite content and isotopic composition as functions of various environmental drivers, we derive the relevant differential equations in an advection–diffusion–reaction context and solve for all relevant species. In deriving our model, we make eight simplifying assumptions (Supplementary Discussion, section 1): (1) all processes are described by one-dimensional reactions^4,26,52–54^; (2) compaction is negligible relative to sedimentation and can be ignored^55,56^; (3) all processes are in steady state (compare with ref. ^57^); (4) bioturbation can be ignored in the sulfidic anoxic zone^38,45,58–64^; (5) methanotrophic sulfate reduction negligibly impacts pyrite isotopic compositions^4,5,52,65,66^; (6) sulfate reduction and sulfide oxidation follow Monod kinetics^8,67–70^; (7) intermediate sulfur species formation is minor^54,71–75^ and (8) MSR is the only sulfur-isotope-fractionating process, and it always operates at thermodynamic equilibrium^17,19,31,76^.

### Dimensional model

Following these assumptions, we first derive our dimensional model before non-dimensionalizing all governing equations. We treat OC and reactive iron decay as following a reactive continuum^29,30,77,78^, and we derive decay-rate coefficient vs time relationships (Supplementary Discussion, section 2). Following this approach, we arrive at the following governing equations:

First, organic carbon content follows

6 $$G(z)={G}_{0}{\left(\frac{aw}{aw+{k}_{\mathrm{G}_{0}}z}\right)}^{a},$$

where *G*(*z*) is OC content in wt% at depth *z*, *G*_0_ and ${k}_{\mathrm{G}_{0}}$ are OC content and reactivity at the top of the sulfidic anoxic zone, *w* is sedimentation rate and *a* is an empirically determined power-law scaling factor (all variables defined in Extended Data Table 1; Supplementary Discussion, section 2.1, provides derivation).

Second, sulfate concentration follows

7 $${D}_\mathrm{S}\frac{{\partial }^{2}S(z)}{\partial {z}^{2}}-w\left[\frac{\partial S(z)}{\partial z}+{f}_\mathrm{G}\frac{\partial G(z)}{\partial z}\left(\frac{S(z)}{{K}_\mathrm{S}+S(z)}\right)\right]=0.$$

where *D*_S_ is sulfate diffusivity, *S*(*z*) is sulfate concentration at depth *z*, *f*_G_ is a factor to convert from wt% OC respired to moles ${{\rm{SO}}}_{4}^{2-}$ reduced and *K*_S_ is the sulfate Monod half-velocity constant (Supplementary Discussion, section 2.2). This is subject to the initial condition

8 $$S(0)={S}_{0},$$

where *S*_0_ = 28 mM in the modern ocean, and the boundary condition

9 $$\mathop{\lim}\limits_{z\to \infty }\frac{\partial S(z)}{\partial z}=0.$$

Third, solid-phase reactive iron (Fe_HR_) in sediments reacts with dissolved-phase sulfide produced by MSR to form FeS and eventually pyrite^70,79^. Similar to the approach used for OC, we describe Fe_HR_ content as

10 $$F(z)={F}_{0}{\left(\frac{bw}{bw+{k}_{\mathrm{F}_{0}}z}\right)}^{b},$$

where *F*(*z*) is reactive iron content in wt% at depth *z*, *F*_0_ and ${k}_{\mathrm{F}_{0}}$ are reactive iron content and reactivity at the top of the sulfidic anoxic zone, and *b* is an empirically determined power-law scaling factor (Supplementary Discussion, section 2.3).

Fourth, sulfide is produced by MSR within the sulfidic anoxic zone, consumed by oxidation by Fe_HR_ to form solid-phase sulfur species, and allowed to move via diffusion (that is, diffusive gradient driven by decreasing concentration above and below the sulfate reduction zone) and sedimentation (that is, burial in sediment porewaters). Sulfide concentration thus follows

11 $${D}_\mathrm{H}\frac{{\partial }^{2}H(z)}{\partial {z}^{2}}-w\left[\frac{\partial H(z)}{\partial z}+{f}_\mathrm{G}\frac{\partial G(z)}{\partial z}\left(\frac{S(z)}{{K}_\mathrm{S}+S(z)}\right)-{f}_\mathrm{F}\frac{\partial F(z)}{\partial z}\left(\frac{H(z)}{{K}_\mathrm{H}+H(z)}\right)\right]=0,$$

where *D*_H_ is sulfide diffusivity, *H*(*z*) is sulfide concentration at depth *z*, *f*_f_ is a factor to convert from wt% reactive iron reduced to moles S^−II^ consumed and *K*_H_ is the sulfide Monod half-velocity constant (Supplementary Discussion, section 2.4). This is subject to the initial condition

12 $$H(0)={H}_{0},$$

where *H*_0_ = 0 mM in the modern ocean and the boundary condition

13 $$\mathop{\lim}\limits_{z\to \infty }\frac{\partial H(z)}{\partial z}=0.$$

Finally, we assume all decayed Fe_HR_ contributes to pyrite formation when S^−II^ is available—a corollary of Assumptions 3 and 7, which preclude the accumulation of intermediate sulfur species. We therefore explicitly set the pyrite formation rate as equal to the sulfide oxidation rate, Ox(*z*, *H*). In making this assumption, our model treats pyrite formation rate as insensitive to formation pathway (that is, S^−II^ or polysulfide pathway)^80,81^. Pyrite content thus follows

14 $$\frac{\partial P(z)}{\partial z}=-\frac{{f}_\mathrm{F}}{{f}_\mathrm{P}}\frac{\partial F(z)}{\partial z}\left(\frac{H(z)}{{K}_\mathrm{H}+H(z)}\right),$$

where *f*_F _/ *f*_P_ is a ratio of conversion factors that reduces to the mass ratio of iron to pyrite (Supplementary Discussion, section 2.5).

In addition to their concentrations/contents, we solve for the sulfur isotopic composition of sulfate, sulfide and pyrite (Supplementary Discussion, section 2.6). Isotopic compositions are written in ‘delta’ notation as

15 $${\delta }^{34}{{\rm{S}}}_\mathrm{A}=\frac{{34\atop}{{\rm{R}}}_\mathrm{A}}{{34\atop}{{\rm{R}}}_{{\rm{VCDT}}}}-1,$$

where ^34^R is the ^34^S/^32^R isotope ration, A is any measured material and VCDT is the Vienna Canyon Diablo Troilite international standard^82^; results are reported in units of ‘per mille’ by multiplying equation (15) by 1,000‰. We specifically solve for each isotopologue of each species individually, and we convert to delta values as functions of depth using equation (15).

For sulfate, equation (7) is rewritten for each isotopologue as

16 $$\begin{array}{l}{D}_\mathrm{S}\frac{{\partial}^{2}\left[{32\atop}S(z)\right]}{\partial {z}^{2}}\\-\,w\left\{\frac{\partial \left[{32\atop}S(z)\right]}{\partial z}-{f}_\mathrm{G}\frac{\partial G(z)}{\partial z}\left(\frac{{32\atop}S(z)}{{34\atop}{\alpha }_{{{\rm{S}}}^{-{\rm{II}}}/{{\rm{SO}}}_{4}^{2-}}\times {34\atop}S(z)+{32\atop}S(z)}\right)\left(\frac{S(z)}{{K}_\mathrm{S}+S(z)}\right)\right\}=0\end{array}$$

and

17 $$\begin{array}{l}{D}_\mathrm{S}\frac{{\partial }^{2}\left[{34\atop}S(z)\right]}{\partial {z}^{2}}\\-\,w\left\{\frac{\partial \left[{34\atop}S(z)\right]}{\partial z}-{f}_\mathrm{G}\frac{\partial G(z)}{\partial z}\left(\frac{{34\atop}{\alpha }_{{{\rm{S}}}^{-{\rm{II}}}/{{\rm{SO}}}_{4}^{2-}}\times {34\atop}S(z)}{{34\atop}{\alpha }_{{{\rm{S}}}^{-{\rm{II}}}/{{\rm{SO}}}_{4}^{2-}}\times {34\atop}S(z)+{32\atop}S(z)}\right)\left(\frac{S(z)}{{K}_\mathrm{S}+S(z)}\right)\right\}=0,\end{array}$$

where ${34\atop}{\alpha }_{{{\rm{S}}}^{-{\rm{II}}}/{{\rm{SO}}}_{4}^{2-}}$ is the temperature-dependent MSR equilibrium fractionation factor between sulfate and sulfide^17,19,31^. The initial condition is rewritten as

18 $${32\atop}S(0)=\frac{1}{1+{34\atop}{{\rm{R}}}_{\mathrm{S}_{0}}}{S}_{0}$$

and

19 $${34\atop}S(0)=\frac{{34\atop}{{\rm{R}}}_{\mathrm{S}_{0}}}{1+{34\atop}{{\rm{R}}}_{\mathrm{S}_{0}}}{S}_{0},$$

where ${34\atop}{{\rm{R}}}_{\mathrm{S}_{0}}$ is the sulfur isotope ratio of seawater sulfate (*δ*^34^S = 28‰ VCDT in the modern ocean); the boundary condition applies equally for both isotopologues.

For sulfide, equation (11) is rewritten for each isotopologue as

20 $$\begin{array}{rcl}&&{D}_\mathrm{H}\frac{{\partial }^{2}\left[{32\atop}H(z)\right]}{\partial {z}^{2}}-w\left\{\frac{\partial \left[{32\atop}H(z)\right]}{\partial z}+{f}_\mathrm{G}\frac{\partial G(z)}{\partial z}\left(\frac{{32\atop}S(z)}{{34\atop}{\alpha }_{{{\rm{S}}}^{-{\rm{II}}}/{{\rm{SO}}}_{4}^{2-}}\times {34\atop}S(z)+{32\atop}S(z)}\right)\left(\frac{S(z)}{{K}_\mathrm{S}+S(z)}\right)\right.\\ &&\left.-{f}_\mathrm{F}\frac{\partial F(z)}{\partial z}\left(\frac{{32\atop}H(z)}{{32\atop}H(z)+{34\atop}H(z)}\right)\left(\frac{H(z)}{{K}_\mathrm{H}+H(z)}\right)\right\}=0\end{array}$$

and

21 $$\begin{array}{rcl}&&{D}_\mathrm{H}\frac{{\partial }^{2}\left[{34\atop}H(z)\right]}{\partial {z}^{2}}-w\left\{\frac{\partial \left[{34\atop}H(z)\right]}{\partial z}+{f}_\mathrm{G}\frac{\partial G(z)}{\partial z}\left(\frac{{34\atop}{\alpha }_{{{\rm{S}}}^{-{\rm{II}}}/{{\rm{SO}}}_{4}^{2-}}\times {34\atop}S(z)}{{34\atop}{\alpha }_{{{\rm{S}}}^{-{\rm{II}}}/{{\rm{SO}}}_{4}^{2-}}\times {34\atop}S(z)+{32\atop}S(z)}\right)\left(\frac{S(z)}{{K}_\mathrm{S}+S(z)}\right)\right.\\ &&\left.-{f}_\mathrm{F}\frac{\partial F(z)}{\partial z}\left(\frac{{34\atop}H(z)}{{32\atop}H(z)+{34\atop}H(z)}\right)\left(\frac{H(z)}{{K}_\mathrm{H}+H(z)}\right)\right\}=0.\end{array}$$

The initial condition is rewritten as

22 $${32\atop}H(0)=\frac{1}{1+{34\atop}{{\rm{R}}}_{\mathrm{H}_{0}}}{H}_{0}$$

and

23 $${34\atop}H(0)=\frac{{34\atop}{{\rm{R}}}_{\mathrm{H}_{0}}}{1+{34\atop}{{\rm{R}}}_{\mathrm{H}_{0}}}{H}_{0},$$

where ${34\atop}{{\rm{R}}}_{\mathrm{H}_{0}}$ is the sulfur isotope ratio of seawater S^−II^ (only applicable when *H*_0_ > 0); the boundary condition applies equally for both isotopologues.

Finally, for pyrite, equation (14) is rewritten for each isotopologue as

24 $$\frac{\partial \left[{32\atop}P(z)\right]}{\partial z}=-\frac{{f}_\mathrm{F}}{{f}_\mathrm{P}}\frac{\partial F(z)}{\partial z}\left(\frac{{32\atop}H(z)}{{32\atop}H(z)+{34\atop}H(z)}\right)\left(\frac{H(z)}{{K}_\mathrm{H}+H(z)}\right)$$

and

25 $$\frac{\partial \left[{34\atop}P(z)\right]}{\partial z}=-\frac{{f}_\mathrm{F}}{{f}_\mathrm{P}}\frac{\partial F(z)}{\partial z}\left(\frac{{34\atop}H(z)}{{32\atop}H(z)+{34\atop}H(z)}\right)\left(\frac{H(z)}{{K}_\mathrm{H}+H(z)}\right).$$

### Non-dimensional model

After deriving our dimensional model, we non-dimensionalize all species concentrations/contents and derive the governing equations as functions of non-dimensional natural variables (Supplementary Discussion, section 3).

First, for organic carbon content, equation (6) is rewritten as

26 $$\Gamma (\zeta )={\Gamma }_{0}{\left(\frac{a}{a+{{\rm{Da}}}^{* }\zeta }\right)}^{a},$$

where *ζ* is the non-dimensional depth, defined as

27 $$\zeta =\sqrt{\frac{{k}_{\mathrm{G}_{0}}}{{D}_\mathrm{S}}}z,$$

*Γ*(*ζ*) is the non-dimensional OC content at depth *ζ*, defined as

28 $$\Gamma (\zeta )=\frac{{f}_\mathrm{G}G(\zeta )}{{S}_{0}},$$

and *Γ*_0_ and Da^*^ are defined in the main text (all non-dimensional variables defined in Extended Data Table 2; Supplementary Discussion, section 3.1, provides derivation).

Second, for sulfate concentration, equation (7) is rewritten as

29 $${{\rm{Da}}}^{* }\frac{{\partial }^{2}\Sigma (\zeta )}{\partial {\zeta }^{2}}-\frac{\partial \Sigma (\zeta )}{\partial \zeta }+\frac{\partial \Gamma (\zeta )}{\partial \zeta }\left(\frac{\Sigma (\zeta )}{{\kappa }_{\Sigma }+\Sigma (\zeta )}\right)=0,$$

where *Σ*(*ζ*) is the non-dimensional sulfate concentration at depth *ζ*, defined as

30 $$\Sigma (\zeta )=\frac{S(\zeta )}{{S}_{0}},$$

and *κ*_*Σ*_ is the non-dimensional sulfate Monod half-velocity constant, defined as

31 $${\kappa }_{\Sigma }=\frac{{K}_\mathrm{S}}{{S}_{0}}.$$

The initial condition is rewritten as *Σ*_0_ ≡ 1, and the boundary condition applies as before (Supplementary Discussion, section 3.2).

Third, for reactive iron content, equation (10) is rewritten as

32 $$\Psi (\zeta )={\Psi }_{0}{\left(\frac{b}{b+\chi {{\rm{Da}}}^{* }\zeta }\right)}^{b},$$

where *Ψ*(*ζ*) is the non-dimensional reactive iron content at depth *ζ*, defined as

33 $$\Psi (\zeta )=\frac{{f}_\mathrm{F}F(\zeta )}{{S}_{0}},$$

*χ* is the ratio of reactive iron to OC initial reactivities, defined as

34 $$\chi =\frac{{k}_{\mathrm{F}_{0}}}{{k}_{\mathrm{G}_{0}}},$$

and *Ψ*_0_ is defined in the main text (Supplementary Discussion, section 3.3).

Fourth, for sulfide concentration, equation (11) is rewritten as

35 $$\Delta {{\rm{Da}}}^{* }\frac{{\partial }^{2}\eta (\zeta )}{\partial {\zeta }^{2}}-\frac{\partial \eta (\zeta )}{\partial \zeta }-\frac{\partial \Gamma (\zeta )}{\partial \zeta }\left(\frac{\Sigma (\zeta )}{{\kappa }_{\Sigma }+\Sigma (\zeta )}\right)+\frac{\partial \Psi (\zeta )}{\partial \zeta }\left(\frac{\eta (\zeta )}{{\kappa }_{\eta }+\eta (\zeta )}\right)=0,$$

where *Δ* is the ratio of diffusivities, defined as

36 $$\Delta =\frac{{D}_\mathrm{H}}{{D}_\mathrm{S}},$$

*η*(*ζ*) is the non-dimensional sulfide concentration at depth *ζ*, defined as

37 $$\eta (\zeta )=\frac{H(\zeta )}{{S}_{0}},$$

and *κ*_*η*_ is the non-dimensional sulfide Monod half-velocity constant, defined as

38 $${\kappa }_{\eta }=\frac{{K}_\mathrm{H}}{{S}_{0}}.$$

The initial condition is rewritten as *η*(0) = *η*_0_ (*η*_0_ = 0 in the fully oxygenated modern ocean), and the boundary condition applies as before (Supplementary Discussion, section 3.4).

Finally, for pyrite content, equation (14) is rewritten as

39 $$\frac{\partial \Pi (\zeta )}{\partial \zeta }=-\frac{\partial \Psi (\zeta )}{\partial \zeta }\left(\frac{\eta (\zeta )}{{\kappa }_{\eta }+\eta (\zeta )}\right),$$

where *Π*(*ζ*) is the non-dimensional pyrite content at depth *ζ*, defined as

40 $$\Pi (\zeta )=\frac{{f}_\mathrm{P}P(\zeta )}{{S}_{0}},$$

and *f*_P_ is a factor to convert from wt% pyrite produced to moles S^−II^ consumed (Supplementary Discussion, section 3.5). Like for the dimensional model, we solve the non-dimensional governing equations for each isotopologue of each species individually, and we convert to delta values as functions of non-dimensional depth (Supplementary Discussion, section 3.6).

For non-dimensional sulfate, equation (29) is rewritten for each isotopologue as

41 $$\begin{array}{l}{{\rm{Da}}}^{* }\frac{{\partial }^{2}\left[{32\atop}\Sigma (\zeta )\right]}{\partial {\zeta }^{2}}-\frac{\partial \left[{32\atop}\Sigma (\zeta )\right]}{\partial \zeta }+\frac{\partial \Gamma (\zeta )}{\partial \zeta }\\\left(\frac{{32\atop}\Sigma (\zeta )}{{34\atop}{\alpha }_{{{\rm{S}}}^{-{\rm{II}}}/{{\rm{SO}}}_{4}^{2-}}\times {34\atop}\Sigma (\zeta )+{32\atop}\Sigma (\zeta )}\right)\left(\frac{\Sigma (\zeta )}{{\kappa }_{\Sigma }+\Sigma (\zeta )}\right)=0\end{array}$$

and

42 $$\begin{array}{l}{{\rm{Da}}}^{* }\frac{{\partial }^{2}\left[{34\atop}\Sigma (\zeta )\right]}{\partial {\zeta }^{2}}-\frac{\partial \left[{34\atop}\Sigma (\zeta )\right]}{\partial \zeta }+\frac{\partial \Gamma (\zeta )}{\partial \zeta }\\\left(\frac{{34\atop}{\alpha }_{{{\rm{S}}}^{-{\rm{II}}}/{{\rm{SO}}}_{4}^{2-}}\times {34\atop}\Sigma (\zeta )}{{34\atop}{\alpha }_{{{\rm{S}}}^{-{\rm{II}}}/{{\rm{SO}}}_{4}^{2-}}\times {34\atop}\Sigma (\zeta )+{32\atop}\Sigma (\zeta )}\right)\left(\frac{\Sigma (\zeta )}{{\kappa }_{\Sigma }+\Sigma (\zeta )}\right)=0.\end{array}$$

The initial condition is rewritten as

43 $${32\atop}\Sigma (0)=\frac{1}{1+{34\atop}{{\rm{R}}}_{{\Sigma }_{0}}}$$

and

44 $${34\atop}\Sigma (0)=\frac{{34\atop}{{\rm{R}}}_{{\Sigma }_{0}}}{1+{34\atop}{{\rm{R}}}_{{\Sigma }_{0}}},$$

where ${\,}^{34}{{\rm{R}}}_{{\Sigma }_{0}}$ is again the sulfur isotope ratio of seawater sulfate, and the same boundary condition applies equally for both isotopologues.

For non-dimensional sulfide, equation (35) is rewritten for each isotopologue as

45 $$\begin{array}{rcl}&&\Delta {{\rm{Da}}}^{* }\frac{{\partial }^{2}\left[{32\atop}\eta (\zeta )\right]}{\partial {\zeta }^{2}}-\frac{\partial \left[{32\atop}\eta (\zeta )\right]}{\partial \zeta }-\frac{\partial \Gamma (\zeta )}{\partial \zeta }\left(\frac{{32\atop}\Sigma (\zeta )}{{34\atop}{\alpha }_{{{\rm{S}}}^{-{\rm{II}}}/{{\rm{SO}}}_{4}^{2-}}\times {34\atop}\Sigma (\zeta )+{32\atop}\Sigma (\zeta )}\right)\left(\frac{\Sigma (\zeta )}{{\kappa }_{\Sigma }+\Sigma (\zeta )}\right)\\ &&+\frac{\partial \Psi (\zeta )}{\partial \zeta }\left(\frac{{32\atop}\eta (\zeta )}{{32\atop}\eta (\zeta )+{34\atop}\eta (\zeta )}\right)\left(\frac{\eta (\zeta )}{{\kappa }_{\eta }+\eta (\zeta )}\right)=0\end{array}$$

and

46 $$\begin{array}{rcl}&&\Delta {{\rm{Da}}}^{* }\frac{{\partial }^{2}\left[{34\atop}\eta (\zeta )\right]}{\partial {\zeta }^{2}}-\frac{\partial \left[{34\atop}\eta (\zeta )\right]}{\partial \zeta }-\frac{\partial \Gamma (\zeta )}{\partial \zeta }\left(\frac{{34\atop}{\alpha }_{{{\rm{S}}}^{-{\rm{II}}}/{{\rm{SO}}}_{4}^{2-}}\times {34\atop}\Sigma (\zeta )}{{34\atop}{\alpha }_{{{\rm{S}}}^{-{\rm{II}}}/{{\rm{SO}}}_{4}^{2-}}\times {34\atop}\Sigma (\zeta )+{32\atop}\Sigma (\zeta )}\right)\left(\frac{\Sigma (\zeta )}{{\kappa }_{\Sigma }+\Sigma (\zeta )}\right)\\ &&+\frac{\partial \Psi (\zeta )}{\partial \zeta }\left(\frac{{34\atop}\eta (\zeta )}{{32\atop}\eta (\zeta )+{34\atop}\eta (\zeta )}\right)\left(\frac{\eta (\zeta )}{{\kappa }_{\eta }+\eta (\zeta )}\right)=0.\end{array}$$

The initial condition is rewritten as

47 $${32\atop}\eta (0)=\frac{1}{1+{34\atop}{{\rm{R}}}_{{\eta }_{0}}}{\eta }_{0}$$

and

48 $${34\atop}\eta (0)=\frac{{34\atop}{{\rm{R}}}_{{\eta }_{0}}}{1+{34\atop}{{\rm{R}}}_{{\eta }_{0}}}{\eta }_{0},$$

where ${\,}^{34}{{\rm{R}}}_{{\eta }_{0}}$ is again the sulfur isotope ratio of seawater S^−II^ (only applicable when *η*_0_ > 0), and the same boundary as above condition applies for both isotopologues.

Finally, for non-dimensional pyrite, equation (39) is rewritten for each isotopologue as

49 $$\frac{\partial \left[{32\atop}\Pi (\zeta )\right]}{\partial \zeta }=-\frac{\partial \Psi (\zeta )}{\partial \zeta }\left(\frac{{32\atop}\eta (\zeta )}{{32\atop}\eta (\zeta )+{34\atop}\eta (\zeta )}\right)\left(\frac{\eta (\zeta )}{{\kappa }_{\eta }+\eta (\zeta )}\right)$$

and

50 $$\frac{\partial \left[{34\atop}\Pi (\zeta )\right]}{\partial \zeta }=-\frac{\partial \Psi (\zeta )}{\partial \zeta }\left(\frac{{34\atop}\eta (\zeta )}{{32\atop}\eta (\zeta )+{34\atop}\eta (\zeta )}\right)\left(\frac{\eta (\zeta )}{{\kappa }_{\eta }+\eta (\zeta )}\right).$$

### Model implementation

After validating the stability of our differential equation solvers and governing equations over the parameter-space region of interest (Supplementary Figs. 22–25 and Supplementary Discussion, section 6), our non-dimensional model results were then compared against measured data from a suite of 216 sedimentary profiles containing at least a subset of the sulfur-cycle content/concentration and isotopic composition data of interest (Supplementary Table 2, Supplementary Figs. 26 and 27 and Supplementary Discussion, section 7). Global signals were then determined by solving our non-dimensional model for each point in the modern ocean using the globally gridded boundary conditions as inputs; globally averaged metrics were calculated as the (weighted) average of all grid points (Supplementary Figs. 28–32 and Supplementary Discussion, section 8). Finally, model sensitivity was determined by resolving the global model after individually increasing or decreasing each boundary condition input value by an amount that corresponds to the measured vs predicted regression uncertainty (Extended Data Fig. 4).

#### Pyrite content in the sulfidic anoxic zone

To calculate integrated pyrite content within the formation zone, we must first define the maximum depth of pyrite formation, ${\zeta }_{\max }$. To do so, we calculate the first depth at which the derivative of pyrite formation decreases beyond a given threshold, *β*. That is, we let

51 $${\zeta }_{\max }=\mathop{\min }\limits_{x\in \zeta }\frac{\partial \Pi }{\partial x}\le \beta .$$

Resulting estimates of average pyrite isotopic composition and, particularly, integrated pyrite content are thus sensitive to our choice of threshold value *β*. Large thresholds may lead to underestimates of pyrite content, formation rate and isotopic composition and vice versa. We therefore test the influence of this threshold on integrated pyrite content within the formation zone, burial rate and isotopic composition. We do this for six different scenarios for high and low OC content in shelf, slope and abyssal settings, respectively. To choose representative sites, we divide pixels of our global OC estimates into ‘high’ and ‘low’ (that is > 1 and < 1 wt% OC). We then divide these into shelf, slope and abyssal batches based on water depth. For each of the three ‘high OC’ datasets, we use the 95th percentile value as a high-yet-representative measure of OC, while using average values for all other boundary conditions within the dataset. Similarly, for each of the three ‘low OC’ datasets, we use the 5th percentile value as a low-yet-representative measure of OC, along with average values for all other boundary conditions within each dataset. Although integrated content will continuously increase with decreasing *β* value by definition (that is, deeper integration always leads to higher total content), we find that pyrite burial flux and isotopic composition remain unchanged for threshold values below *β* = 5 × 10^−4^ (Supplementary Fig. 28). We thus choose this value when calculating all global estimates below.

We solve all relevant equations for a global grid to a maximum non-dimensional depth of *ζ* = 5. We then limit solutions to the pyrite formation zone defined by the threshold above (equation (51), where ${\zeta }_{\max } < 5$). Although the pyrite formation zone might theoretically extend beyond this maximum depth in some deep-sea settings (that is, ${\zeta }_{\max } > 5$; Supplementary Fig. 28c), this will exert minimal influence on our results because pyrite does not primarily form in these settings. Furthermore, median sediment thickness in these regions is *z*_tot_ = 243 m. Together with a median OC reactivity ${k}_{\mathrm{G}_{{\rm{SW}}}}=6.2\times 1{0}^{-7}\,{{\rm{yr}}}^{-1}$ and a median sulfate diffusivity of *D*_S_ = 103 cm^2^ yr^−1^, this yields a representative non-dimensional maximum sediment thickness of *ζ* = 1.8. Thus, although pyrite formation in these regions is not expected to cross *β* = 5 × 10^−4^ until *ζ* ≫ 5, such large non-dimensional depths are physically unrealistic. Our choice of maximum model solution depth either exceeds ${\zeta }_{\max }$ or exceeds the total sediment thickness for all regions of the oceans and is therefore robust.

To generate global maps of pyrite content, we calculate total moles of pyrite within the sulfidic anoxic zone by first converting non-dimensional pyrite content back to dimensional pyrite content. We then convert from wt% FeS_2_ at each depth in the sediment column to *n*_P_, the depth-integrated moles of pyrite per unit area (that is, mol FeS_2_cm^−2^), by solving the integral:

52 $${n}_\mathrm{P}=\frac{1}{100}\frac{{\rho }_{{\rm{sol}}}(1-\phi )A}{119.98\,{\rm{g}}\,{{\rm{FeS}}}_{2}\,{{\rm{mol}}}^{-1}}\mathop{\int}\nolimits_{0}^{{\zeta }_{\max }}P(\zeta )d\zeta ,$$

where *A* is the area of the gridded map pixel in cm^2^. By contrast, when generating heat maps, we integrate non-dimensional pyrite content over the sulfidic anoxic zone (that is, $\mathop{\int}\nolimits_{0}^{{\zeta }_{\max }}\Pi (\zeta )d\zeta$). Resulting *n*_P_ and ${\zeta }_{\max }$ maps are shown in Extended Data Fig. 5.

#### Pyrite burial flux

Because we assume that pyrite formation is in steady state (Assumption 3) and that detrital pyrite deposition to the seafloor is negligible, we treat the total amount of pyrite formed throughout the sediment column as equal to the flux of pyrite that leaves the sulfidic anoxic zone by mass balance (that is, formation = output). We therefore calculate *J*_P_, the pyrite formation flux (= burial flux; that is, mol FeS_2_ cm^−2^ yr^−1^), as

53 $${J}_\mathrm{P}=w\frac{1}{100}\frac{{\rho }_{{\rm{sol}}}(1-\phi )A}{119.98\,{\rm{g}}\,{{\rm{FeS}}}_{2}\,{{\rm{mol}}}^{-1}}P({\zeta }_{\max }).$$

#### Pyrite isotopic composition

Finally, we calculate global weighted-averaged pyrite isotopic compositions, *δ*^34^S_mean_. To do so, we assume an isotopic composition equal to that of buried pyrite; that is, we take the *δ*^34^S value of pyrite at the formation depth ${\zeta }_{\max }$. We then calculate the global weighted average as

54 $${\delta }^{34}{{\rm{S}}}_{{\rm{mean}}}=\frac{{\sum }_{i}{J}_\mathrm{P}^{i}\times {\delta }^{34}{{\rm{S}}}_{\Pi ({\zeta }_{\max })}^{i}}{{\sum }_{i}{J}_\mathrm{P}^{i}},$$

where *i* refers to each grid cell in our globally gridded dataset and *δ*^34^S${}_{\Pi ({\zeta }_{\max })}$ is the pyrite isotopic composition at depth ${\zeta }_{\max }$.

### Sensitivity tests

To validate the robustness of our model results, we perform a series of sensitivity tests (further details in Supplementary Discussion, section 8). We specifically tested pyrite formation rate and isotopic composition sensitivity to uncertainty in: (1) porosity, *ϕ*; (2) sedimentation rate, *w*; (3) OC content at the sediment–water interface, *G*_sw_; (4) reactive iron content at the sediment–water interface, *F*_sw_; (5) sulfate and sulfide diffusivities, *D*_S_ and *D*_H_; (6) depth of net sulfate reduction onset, that is, oxygen-penetration depth or mixed-layer depth (*z*_OPD_ vs *z*_MLD_); (7) relative reactivity of iron to organic carbon, *χ*; and OC reactivity at the sediment–water interface, ${k}_{\mathrm{G}_{{\rm{sw}}}}$. For test (1), we uniformly decreased *ϕ* by 7.7%, the maximum estimate of compaction within the sulfidic anoxic zone as predicted by ref. ^56^. For most other tests, we independently increase/decrease each boundary condition of interest using the RMSE of globally predicted vs locally measured values at our calibration sites (that is, regression RMSEs in Supplementary Figs. 6–16). Test (5) was done similarly by re-calculating temperature-dependent diffusivity using propagating globally predicted vs locally measured temperature RMSE (Supplementary Fig. 5). Finally, because several parameters are themselves calculated as functions of sedimentation rate (that is, *z*_OPD_, *z*_MLD_, ${k}_{\mathrm{G}_{{\rm{sw}}}}$, ${k}_{\mathrm{F}_{{\rm{sw}}}}$), we assessed the importance of this dependence by performing test (2) both by holding these parameters constant and by re-calculating all dependent parameters after propagating sedimentation-rate RMSE (Supplementary Fig. 31).

### Earth-history compilation

Data for the Phanerozoic pyrite isotopic compilation were taken from refs. ^7,83–87^. Seawater sulfate isotopic compositions were taken from ref. ^88^; values derived from carbonate-associated sulfate were excluded, as these are often diagenetically altered^89^. To visualize Earth-history trends across scattered data, sulfate and pyrite isotopic data were first smoothed using a locally estimated scatterplot smoothing function, which performs local polynomial regression. Smoothing factors of 0.2 and 0.5 were chosen for sulfate and pyrite records, respectively (that is, 20% and 50% of data points were used in each local regression). These specific smoothing factors were selected because they represent the optimal balance between reducing noise and preserving the underlying trends in the data. Smoothed seawater sulfate isotopic composition were then linearly interpolated to the timestamps of each pyrite isotopic composition measurement to calculate *Δ*_pyrite_ as a function of time (Fig. 4). Finally, confidence intervals were computed by performing 1,000 bootstrap resampling iterations. For each iteration, the time series was randomly resampled, smoothed, interpolated and *Δ*_pyrite_ was calculated; *Δ*_pyrite_ trends for all iterations were then used to calculate 5th to 95th percentile confidence intervals.

## Online content

Any methods, additional references, Nature Portfolio reporting summaries, source data, extended data, supplementary information, acknowledgements, peer review information; details of author contributions and competing interests; and statements of data and code availability are available at 10.1038/s41561-025-01855-5.

## Supplementary information

Supplementary Information Supplementary Discussion, Supplementary Tables 1 and 2, Supplementary Figs. 1–33 and Supplementary References 106–211 (continued numbering from main text and Methods).

Supplementary Data 1 Globally gridded pyrite model results for the ‘standard’ model runs (25 arcminute resolution), including: formation depth (*z*_max_, (cm)), burial flux (pyrite_burial_rate, (g cm^−2^ y^−1^)), depth-integrated total content (total_mol_pyrite, (mol cm^−2^)) and (weighted) depth-average isotopic composition (delta_pyrite, (‰)). All iron- and sulfur species input data (concentrations, contents, isotopic compositions) and sedimentological data, where available, for all sites compiled for model validation in this study.

## Figures and Tables

**Extended Data Table 1:**
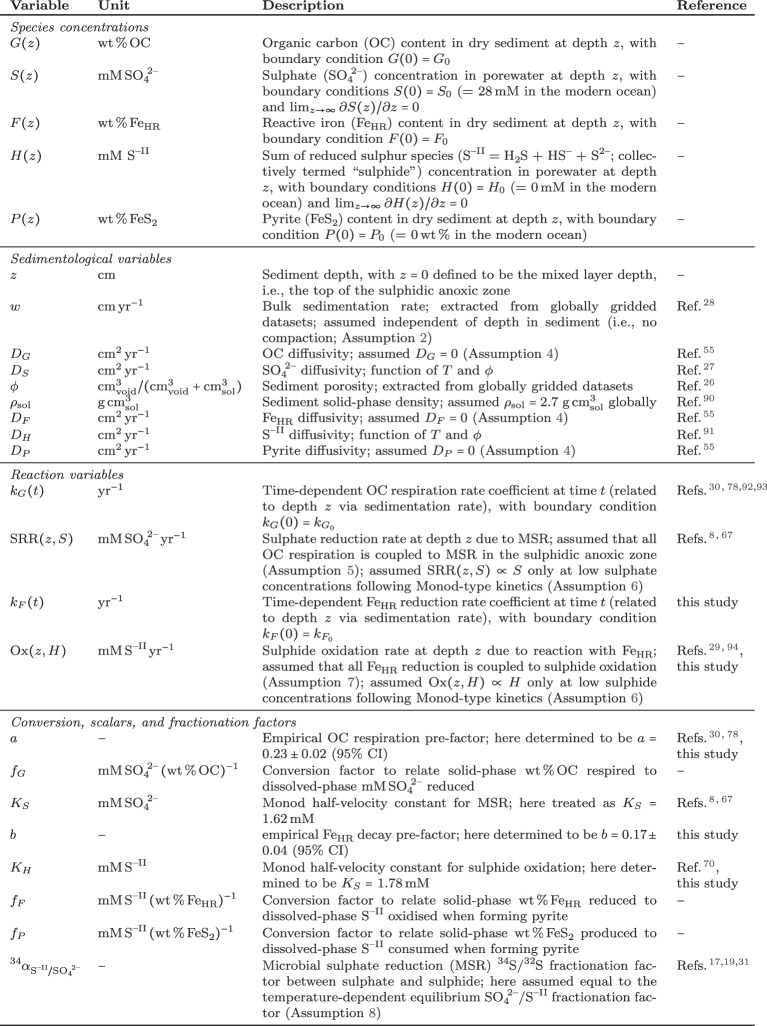
Dimensional variables

All variables used in the dimensional model, including units, descriptions, and references from which assumed or inputted values were extracted. See Supplementary Discussion, section 1 for details on assumptions.

**Extended Data Table 2:**
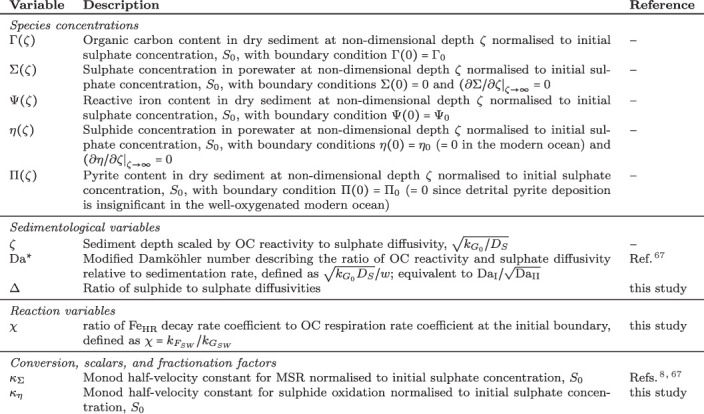
Non-dimensional variables

All variables used in the non-dimensional model, including descriptions and references from which assumed or inputted values were extracted.

**Extended Data Table 3:**
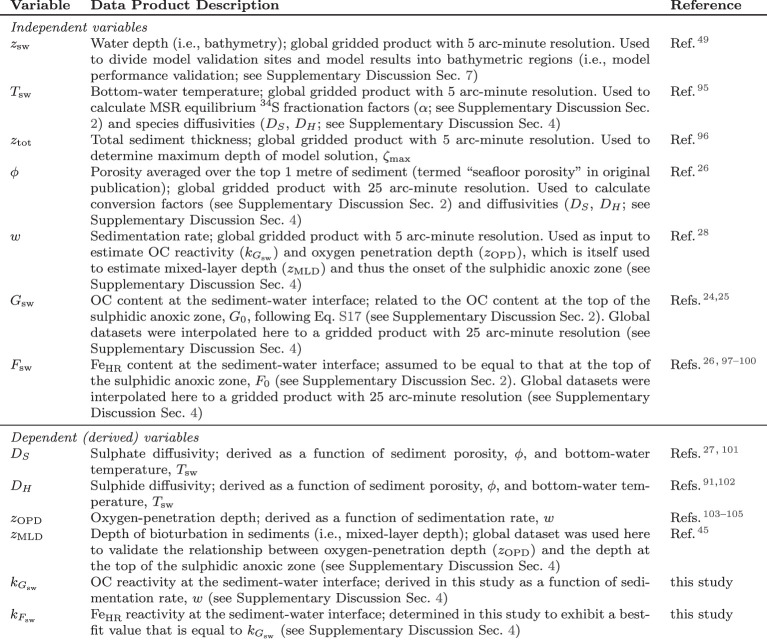
Literature sources of global boundary conditions

Sources of estimated global and gridded input variable data used for model validation and global maps generated in this study.

## Data Availability

The datasets used to validate our model, define boundary conditions and set parameters for this study are available via Zenodo at https://zenodo.org/records/16265000 (ref. ^90^).

## Code availability

All code used to process data, generate results and make figures (written in MATLAB) can be accessed via Zenodo at https://zenodo.org/records/14808279 (ref. ^91^).
