# Supplementary material for: Sedimentary conditions drive modern pyrite burial flux to exceed oxidation
Source: Nat Geosci. 2025 Dec 12;19(1):99–105. doi: 10.1038/s41561-025-01855-5 (PMC12791017; doi:10.1038/s41561-025-01855-5)
Supplement: Supplementary file 1 — Supplementary Discussion, Supplementary Tables 1 and 2, Supplementary Figs. 1–33 and Supplementary References 106–211 (continued numbering from main text and Methods). [file 41561_2025_1855_MOESM1_ESM.pdf]

nature geoscience

Supplementary information

https://doi.org/10.1038/s41561-025-01855-5

Sedimentary conditions drive modern pyrite burial flux to exceed oxidation

In the format provided by the authors and unedited

Supplementary Discussion

Table of contents:

1 General considerations and model assumptions . . . SI-2
2 Dimensional model derivation . . . SI-4
  2.1 Organic carbon content . . . SI-4
  2.2 Sulphate concentration . . . SI-6
  2.3 Reactive iron content . . . SI-8
  2.4 Sulphide concentration . . . SI-11
  2.5 Pyrite content . . . SI-13
  2.6 Isotopic compositions . . . SI-14
3 Non-dimensional model derivation . . . SI-18
  3.1 Organic carbon content . . . SI-19
  3.2 Sulphate concentration . . . SI-20
  3.3 Reactive iron content . . . SI-20
  3.4 Sulphide concentration . . . SI-21
  3.5 Pyrite content . . . SI-21
  3.6 Isotopic compositions . . . SI-21
4 Dimensional boundary conditions . . . SI-24
  4.1 Independent variables . . . SI-24
  4.2 Dependent (derived) variables . . . SI-27
5 Non-dimensional boundary conditions . . . SI-30
6 Solver verification . . . SI-31
  6.1 Shooting function . . . SI-32
  6.2 Manufactured solutions . . . SI-32
7 Model validation and performance . . . SI-36
  7.1 Comparison to global profile compilation . . . SI-36
  7.2 Measured vs. interpolated boundary conditions . . . SI-38
8 Model interpretation and sensitivity . . . SI-39
  8.1 Parameter space heat maps . . . SI-39
  8.2 Sensitivity tests . . . SI-41

1 General considerations and model assumptions

In setting up our model framework, we prioritise simplicity to facilitate extraction and interpretation of the local and global processes that are most important for driving pyrite formation rate and isotopic composition. In doing so, we make several assumptions. We introduce these here, including justification:

1. *All processes are described by one-dimensional reactions.* We ignore any lateral variability due to phenomena such as small-scale seafloor topography (e.g., migrating dunes and ripples)[57] and benthic faunal activity (e.g., discrete worm burrows)[108], and we describe all processes with one-dimensional diffusion-advection-reaction equations as functions of sediment depth. Although lateral variability can become important at the centi- to decimetre scale—particularly in response to redox gradients induced by faunal burrowing[108–110]—this is largely restricted to oxic and upper non-sulphidic anoxic sediments. A corollary of this assumption states that net sulphate reduction (i.e., observable decrease in porewater sulphate concentrations) only occurs below the non-sulphidic anoxic zone. This is validated by radiotracer-derived sulphate reduction rate (SRR) measurements[4, 52–54]. In particular, Ref.[54] demonstrated that gross SRR is highest in oxic and non-sulphidic anoxic surface sediments, but that it is quantitatively balanced by re-oxidation of dissolved sulphide species (H_2S, HS^-, S^{2-}, collectively S^{-II}) in this zone, leading to negligible net sulphate loss. We therefore implicitly treat any lateral variability as occurring in the upper oxic and non-sulphidic anoxic zone, above our region of interest, and we only consider reactions occurring below this zone (see also Assumption 4, below).

2. *Compaction is negligible relative to sedimentation and can be ignored.* We treat porosity, ϕ, as constant with depth within the sediment column. This assumption is not valid within the upper centi- to decimetres of sediment, where compaction can lead to large porosity decreases[55]. However, by using ϕ values averaged over the top metre of sediment (Ref.[26]; Extended Data Table 3), we implicitly account for compaction in this region. Below this zone, compaction has been shown to decrease as an exponential function of depth[56]. We therefore estimate the magnitude of bias due to this assumption by using the compaction equation from Ref.[56] (averaged over all lithologies) and maximum sulphate penetration depths of 30 m, 187 m, and 217 m for shelf (water depth ≤200 m), slope (200 m < water depth ≤ 2000 m), and abyssal (water depth >2000 m) sites included in our dataset (Supplementary Table 2; Supplementary Data). Depth-averaged ϕ within the sulphidic anoxic zone decreases by a maximum of 1.1 %, 6.7 %, and 7.7 % in these regions, respectively. Compaction thus exerts only a minor influence on estimated pyrite burial fluxes and isotopic compositions (see Supplementary Discussion Sec. 8).

3. *All sediment-column processes are in steady state over pyrite-formation timescales.* This assumption is necessary due to the fact that global boundary conditions are only available for the modern ocean. We thus assume all boundary conditions and parameters do not experience time-dependent variability over the timescales of pyrite formation (i.e., ~10^4 yr in continental shelves, ~10^7 yr in abyssal plains). Although sedimentological variability on glacial/interglacial (~10^5 yr) timescales has been shown to drive changes in pyrite isotopic compositions in shallow-water settings (≤350 m water depth), such variability has not been observed over shorter timescales at these sites nor over multi-million year timescales in nearby deep-water settings (≥2000 m)[13,14,16]. Such observations support our steady-state assumption at the global scale. However, this simplification might lead to inaccurate predictions at sites with large lateral sediment transport (e.g., Amazon mud belt)[57] or those with obvious turbidites or other short-term variations in sedimentary regime. Importantly, this differs fundamentally from the steady-state assumption of traditional box models, which treat all sulphur-cycle sources and sinks as constant over the timescale of sulphate residence in the ocean (i.e., ~ 10^6 to 10^7 yr).

4. *Bioturbation can be ignored in the sulphidic anoxic zone.* We only consider reactions in the anoxic sediment column, beneath the oxygenated regions where most biomixing and bioirrigation—collectively termed bioturbation—occur[58,59]. Anoxic sediment is further subdivided into the upper non-sulphidic (or "suboxic") and lower sulphidic regions[60,61], with bioturbation often extending into the non-sulphidic zone[38, 62]. Here, we treat this non-sulphidic anoxic zone part of the upper oxygenated region, assuming it is periodically oxygenated through bioturbation. This treatment is supported by several theoretical and field studies, which show that periodic oxygen exposure leads to sediment properties (e.g., respiration rates, nutrient retention, sulphur speciation, and iron oxide content) that are more similar to fully oxygenated, rather than fully anoxic, regions[58,59,63,64]. We therefore

equate the depth of maximum bioturbation[45]—which can become ~10-20× larger than the oxygen penetration depth at any given time[38, 58,62]—with the depth of sulphidic anoxic zone onset (see Supplementary Discussion Sec. 4). We term this the "mixed-layer depth." In this framework, bioturbation is negligible within the sulphidic anoxic zone by definition.

5. *Methanotrophic sulphate reduction negligibly impacts bulk pyrite isotopic compositions.* To quantify SRR at each depth, we assume that all organic carbon (OC) respiration within the sulphidic anoxic zone is driven by microbial sulphate reduction (MSR), and we ignore alternative metabolisms (see also Assumption 7, below). This treatment implicitly includes methanotrophic (i.e., CH_4-consuming) sulphate reduction[4,38] as a component of total SRR. That is, since CH_4 is itself produced by OC fermentation, methanotrophic MSR rate remains directly controlled by OC degradation rate, albeit via a CH_4 intermediate[52]. Nevertheless, since OC fermentation occurs below the sulphate-containing zone, oxidation of this upward-diffusing CH_4 can shift the locus of sulphate reduction deeper into the sediment column, toward the sulphate-methane transition (SMT) zone[50]. Such a vertical shift may impact resulting pyrite isotopic compositions. However, both radiotracer-based SRR estimates[38, 52,65,66] and recent sulphate porewater profile compilations[5] indicate that MSR within the SMT zone (i.e., methanotrophic MSR) contributes only ~1 to 10% of total sulphate reduction. We thus assume that any shift in the locus of MSR toward the SMT zone negligibly impacts pyrite isotopic compositions.

6. *Sulphate reduction and sulphide oxidation follow Monod kinetics.* Such treatment is commonly applied in diagenetic models. This follows from the observation the SRR and sulphide oxidation rates decrease as Monod-type functions of sulphate and sulphide concentrations, respectively, as these concentrations approach zero[8 67–70]. In contrast, MSR and sulphide oxidation rate become independent of sulphate and sulphide concentrations above a certain threshold—termed the Monod half-velocity constant—and instead follow first-order reactions as functions of OC and reactive iron decay, respectively[111–113] (see Supplementary Discussion Sec. 2 for derivation and validation).

7. *Intermediate sulphur species formation is minor.* In making this assumption, we ignore re-oxidation of sulphide to oxyanions (i.e., SO_3^{2-}, $S_2O_3^{2-}$, SO_4^{2-}) and solid-phase species that do not participate in further reactions but instead accumulate in sediment (i.e., elemental and organic sulphur). Although such reactions are well-documented to exist in nature[71,73,74], this treatment is justified here for two reasons: (i) *Sulphur mass-balance considerations*: Sulphide re-oxidation to sulphoxyanions has been extensively shown to be low or absent in the sulphidic anoxic zone of natural sediments; this process only becomes significant within the upper oxic and non-sulphidic anoxic (i.e., bioturbated) zones[58,72,74, 113–117]. Furthermore, measured pyrite contents are consistently ≥10-fold higher than those of solid-phase elemental sulphur in both experiments[54,72,73] and natural sediments, including those compiled for this study (Supplementary Table 2; Supplementary Data). Finally, although solid-phase organic sulphur content can approach ≈50% of co-located pyrite in some settings[73], relatively constant concentrations with sediment depth—combined with the observation of rapid organic carbon sulphurisation in sinking marine particles[118] and the fact that pyrite formation is generally kinetically favoured over organic matter sulphurisation in marine sediments[119]—suggests this is at least partially inherited from the water column rather than entirely produced in situ. Low observed sulphide re-oxidation rates, combined with low elemental sulphur and in-situ produced organic sulphur contents, imply that pyrite formation and upward diffusion represent the dominant pathways of sulphide loss from sulphidic anoxic sediments. (ii) *Isotopic considerations*: Although isotopically unique[31], sulphoxyanion concentrations are typically low relative to sulphide in the sulphidic anoxic zones of marine sediments[71,74]. Their formation is thus not expected to significantly impact the isotopic composition of the residual sulphide reservoir. Similarly, in addition to its relatively low contents, elemental sulphur in marine sediments has been shown to rapidly and abiotically undergo sulphur-isotope exchange with sulphide and polysulphides[75]. Thus, because this reservoir is isotopically "open" to sulphide, it does not impact sulphide isotopic compositions. Finally, although repeated redox cycling has been invoked to explain large isotopic offsets between sulphate and sulphide in sediments[120], an updated understanding of MSR fractionation factors has challenged this interpretation[17,19]. Thus, while inclusion of intermediate species may further improve model performance, both mass-balance and isotope considerations—in addition to the fact that such data are lacking in most marine porewater profiles—justifies their exclusion here.

8. *MSR is the only sulphur-fractionating process, and it always operates at thermodynamic equilibrium.* Pyrite isotopic compositions can be influenced by fractionation resulting from: (i) differential

diffusion of sulphate or sulphide isotopologues, (ii) sulphide oxidation to intermediate species, (iii) sulphide oxidation and transformation to pyrite, and (iv) MSR. While this interpretation may be complicated by the proposed importance of ^{34}S-enriched organic sulphur as a source of sulphide—but not pyrite—in some marine sediments[73], we do not explicitly consider this process here (see also Assumption 7). We do not explicitly consider isotopologue-specific diffusion coefficients, as these differences are shown to lead to negligible fractionation and can be effectively ignored. Furthermore, it is well-established that sulphide re-oxidation to pyrite and elemental sulphur negligibly fractionates sulphur isotopes[76,121]. This leaves MSR as the main fractionating reaction during pyrite formation. MSR sulphur-isotope fractionation is canonically interpreted to be a function of cell-specific SRR, with a maximum fractionation factor of ≈50 ‰ observed in slow-growing laboratory cultures[7,8,121] (see Ref.[4] for compilation of laboratory-derived fractionation factors). However, it has recently been recognised that sulphate-reducing bacteria in natural sediments typically operate at orders-of-magnitude slower cell-specific SRR than those grown in laboratory cultures[19]. Such slow and reversible sulphate reduction leads to the expression of a temperature-dependent, equilibrium sulphur isotope fractionation factor of ≈65 to 80 ‰[31]. Following this interpretation, recent models have concluded that MSR operates at this equilibrium limit in nearly all modern marine sediments[17]. We adopt this approach here.

In what follows, we first derive the governing equations for our dimensional pyrite formation model in light of our assumptions (Supplementary Discussion Sec. 2). Next, we utilise these governing equations to derive our non-dimensional model (Supplementary Discussion Sec. 3). We then describe and compile the necessary global dimensional (Supplementary Discussion Sec. 4) and non-dimensional (Supplementary Discussion Sec. 5) boundary conditions to solve our model at all points in the modern ocean. Finally, we detail the computational implementation and verification of our numerical solvers (Supplementary Discussion Sec. 6 and 7), and we assess resulting model performance—including boundary-condition sensitivity tests—when compared to a global dataset (Supplementary Discussion Secs. 7-8).

2 Dimensional model derivation

2.1 Organic carbon content

Following Assumption 1 (all assumptions referred to here are described in Methods), we describe organic carbon (OC) content at time t and depth z in a sediment column by the one-dimensional diffusion-advection-reaction equation[55]:

$$\frac{\partial G(t,z)}{\partial t} = D_G \frac{\partial^2 G(t,z)}{\partial z^2} - w\frac{\partial G(t,z)}{\partial z} - k_G(t)G(t,z), \tag{S1}$$

where $G(t,z)$ is OC content, D_G is OC diffusivity, w is sedimentation rate, and $k_G(t)$ is the time-dependent OC respiration rate coefficient[30] (see Extended Data Table 1 for definitions and units). Because we ignore compaction (Assumption 2), Eq. S1 treats D_G and w as independent of depth. We additionally treat depositional setting as constant through time (Assumption 3), such that

$$\frac{\partial G(t,z)}{\partial t} = 0. \tag{S2}$$

Finally, we ignore bioturbation within the sulphidic anoxic zone by setting $D_G = 0$ (Assumption 4). Equation. S1 then becomes

$$\frac{\partial G(z)}{\partial z} = -\frac{k_G(t)}{w}G(z), \tag{S3}$$

subject to the initial condition

$$G(0) = G_0, \tag{S4}$$

where $z = 0$ is defined as the mixed layer depth[45], i.e., the top of the sulphidic anoxic zone. To evaluate Eq. S3, we first constrain $k_G(t)$ at all t as a function of z. It has been shown that $k_G(t)$ decreases with t since the most reactive OC is respired first followed by less reactive material[30,78,92,93]. Regardless of the underlying mechanism driving this phenomenon (but see Refs.[122–124] for mechanistic discussion), Refs.[30,78]

empirically observed that OC respiration-rate coefficients in laboratory experiments and sediment cores decrease as

$$k_G(t) = at^{[-0.95,-0.985]} \approx \frac{a}{t}, \tag{S5}$$

where t is the time since OC formation, a is a regression constant, and square brackets indicate the range of power-law slopes observed when data were fit using ordinary least squares (OLS). To obviate the known OLS bias toward shallow slopes when both x and y variables contain measurement error, we re-analysed data from Ref.[78] using geometric mean (GM) regression[125] while forcing a slope of −1. This yields $a = 0.23 \pm 0.02$ (95% confidence interval, CI), which we utilise throughout our model (Extended Data Table 1).
To determine $k_G(t)$ as a function of z, which is defined relative to mixed-layer depth z_{MLD}, we first introduce absolute sedimentary depth starting at the top of the sediment column as

$$\tilde{z} = z + z_{\mathrm{MLD}}, \tag{S6}$$

where $\tilde{z} = 0$ at the sediment-water interface. This is related to sedimentation rate by

$$\tilde{z} = w\tilde{t}, \tag{S7}$$

and $\tilde{t}$ is the absolute sedimentary time starting at the sediment-water interface, defined as

$$\tilde{t} = t - t_{\mathrm{sw}}, \tag{S8}$$

where t_{sw} is the time that has passed since OC was formed but prior to deposition at the sediment-water interface ("apparent initial age")[30,78]. Rearranging Eqs. S7-S8 and substituting into Eq. S5 yields

$$\begin{aligned} k_G(t) \approx \frac{a}{t} &= \frac{a}{t_{\mathrm{sw}} + \tilde{t}} \\ &= \frac{a}{t_{\mathrm{sw}} + \frac{\tilde{z}}{w}}. \end{aligned} \tag{S9}$$

The respiration-rate coefficient at the sediment-water interface is then calculated from Eq. S9 as

$$k_G(t(\tilde{z} = 0)) = k_{G_{\mathrm{sw}}} = \frac{a}{t_{\mathrm{sw}}}. \tag{S10}$$

Rearranging Eq. S10 and substituting into Eq. S9 thus yields

$$k_G(t(\tilde{z})) = \frac{a}{\frac{a}{k_{G_{\mathrm{sw}}}} + \frac{\tilde{z}}{w}}. \tag{S11}$$

Substituting Eq. S11 into Eq. S3 and utilising the definition of $\tilde{z}$, we arrive at the first-order ordinary differential equation

$$\frac{\partial G(\tilde{z})}{\partial \tilde{z}} = \left(\frac{-a}{w\frac{a}{k_{G_{\mathrm{sw}}}} + \tilde{z}} \right) G(\tilde{z}), \tag{S12}$$

which has the analytical solution

$$G(\tilde{z}) = G_{\mathrm{sw}} \left(\frac{aw}{aw + k_{G_{\mathrm{sw}}}\tilde{z}} \right)^a, \tag{S13}$$

where G_{sw} is the OC content at the sediment-water interface.

Because we are interested in processes occurring in the sulphidic anoxic zone, we re-scale Eqs. S11-S13 to be defined relative to z rather than $\tilde{z}$. First, substituting Eq. S6 into S11 yields

$$k_G(t(z)) = \frac{a}{\frac{a}{k_{G_{\text{sw}}}} + \frac{z+z_{\text{MLD}}}{w}}. \tag{S14}$$

The respiration rate coefficient at the top of the sulphidic anoxic zone is then calculated from Eq. S14 as a function of $k_{G_{\text{sw}}}$, w, and z_{MLD} as

$$k_G(t(z=0)) = k_{G_0} = \frac{a}{\frac{a}{k_{G_{\text{sw}}}} + \frac{z_{\text{MLD}}}{w}}. \tag{S15}$$

Rearranging Eq. S15 for $k_{G_{\text{sw}}}$, substituting into Eq. S11, and utilising the definition of $\tilde{z}$, we arrive at

$$k_G(t(z)) = \frac{a}{\frac{a}{k_{G_0}} + \frac{z}{w}}, \tag{S16}$$

which defines the OC respiration rate in the sulphidic anoxic zone as a function of z. Next, we determine the OC content at the top of the sulphidic anoxic zone as a function of $k_{G_{\text{sw}}}$, G_{sw}, w, and z_{MLD} by substituting Eq. S6 into Eq. S13 and setting $z = 0$, which yields

$$G(z=0) = G_0 = G_{\text{sw}} \left(\frac{aw}{aw + k_{G_{\text{sw}}} z_{\text{MLD}}} \right)^a. \tag{S17}$$

Finally, we substitute Eqs. S15 into Eq. S12 and utilise the definition of $\tilde{z}$ to arrive at the first-order ordinary differential equation

$$\frac{\partial G(z)}{\partial z} = \left(\frac{-a}{w\frac{a}{k_{G_0}} + z} \right) G(z), \tag{S18}$$

which has the analytical solution

$$G(z) = G_0 \left(\frac{aw}{aw + k_{G_0} z} \right)^a. \tag{S19}$$

Equations S18-S19 define our dimensional OC respiration model. This allows us to describe OC respiration using only empirically constrained constants (a) and measured variables that can be interpolated using global datasets (w, z_{MLD}, $k_{G_{\text{sw}}}$, G_{sw}; see Supplementary Discussion Sec. 4) as inputs. Our model thus requires no free fitting parameters. Furthermore, because OC reactivity and content are often reported at the sediment-water interface rather than at z_{MLD} (i.e., "core-top" measurements), we directly calculate $k_{G_{\text{sw}}}$ and G_{sw} when compiling such global boundary conditions. We then utilise Eqs. S15 and S17, along with global w and z_{MLD} estimates, to rescale these to k_{G_0} and G_0, which are the parameters of interest for our model.

2.2 Sulphate concentration

Sulphate moves downward from the overlying water column into marine sediments via diffusion (i.e., diffusive gradient driven by decreasing concentration with depth) due to microbial sulphate reduction (MSR) and via sedimentation (i.e., burial in sediment porewaters). Similar to OC, sulphate concentration at time t and depth z in a sediment column can be described by the one-dimensional diffusion-advection-reaction equation (Assumption 1)[55]:

$$\frac{\partial S(t,z)}{\partial t} = D_S \frac{\partial^2 S(t,z)}{\partial z^2} - w\frac{\partial S(t,z)}{\partial z} - \text{SRR}(z,S), \tag{S20}$$

where $S(t,z)$ is sulphate concentration, D_S is sulphate diffusivity[27], $\text{SRR}(z,S)$ is the net sulphate reduction rate due to MSR at depth z and sulphate concentration S (Extended Data Table 1), and we again

ignore sediment compaction (Assumption 2). Then, following Assumption 3 such that

$$\frac{\partial S(t,z)}{\partial t} = 0, \tag{S21}$$

Eq. S20 simplifies to the second-order ordinary differential equation

$$D_S \frac{\partial^2 S(z)}{\partial z^2} - w \frac{\partial S(z)}{\partial z} - \mathrm{SRR}(z,S) = 0, \tag{S22}$$

subject to the initial condition

$$S(0) = S_0, \tag{S23}$$

where $S_0 = 28\,\mathrm{mM}$ in the modern ocean[7], and the boundary condition

$$\lim_{z\to\infty} \frac{\partial S(z)}{\partial z} = 0. \tag{S24}$$

Equation S24 states that, independent of the final sulphate concentration, $\partial S/\partial z \to 0$ below the sulphidic anoxic zone, i.e., where net reduction no longer occurs and diffusion gradients become insignificant. (Although "cryptic" reduction and re-oxidation may still occur below this zone, this process does not lead to net reduction and thus has no impact on sulphate diffusion gradients[53].)
Following Assumption 5, we treat all MSR as organoclastic such that net sulphate reduction rate is always proportional to OC respiration rate. This leads to

$$\mathrm{SRR}(z,S) \propto k_G(t)G(z), \tag{S25}$$

where the proportionality arises from the need for a scaling term to convert from solid-phase wt % OC in sediment particles to dissolved-phase $\mathrm{mM\,SO_4^{2-}}$ in interconnected sediment porewaters. This is achieved in three steps: first, we convert wt % OC to $\mathrm{mol\,OC\,(g\,sol)^{-1}}$, where g sol is the mass of total solids, using the factor

$$L_C = \frac{1}{100}\frac{1}{12}. \tag{S26}$$

Second, we convert $\mathrm{mol\,OC\,(g\,sol)^{-1}}$ to $\mathrm{mol\,OC\,(V_{void})^{-1}}$ using the porosity, defined as

$$\phi = \frac{V_{\mathrm{void}}}{V_{\mathrm{void}} + V_{\mathrm{sol}}}, \tag{S27}$$

where V_{void} is the volume of voids (i.e., porewaters) and V_{sol} is the volume of solids in a given volume of total sediment. Utilising the definition of density and substituting into Eq. S27 yields

$$\frac{m_{\mathrm{sol}}}{V_{\mathrm{void}}} = \rho_{\mathrm{sol}}\left(\frac{1-\phi}{\phi}\right), \tag{S28}$$

where m_{sol} is the mass and ρ_{sol} the density of the solid phase[126]. Third, to convert mol OC respired to $\mathrm{mol\,SO_4^{2-}}$ reduced, we use the MSR stoichiometry defined in Eq. 1 (Ref.[38]) and define the conversion factor

$$L_G = \frac{1}{1.7} \times 10^6, \tag{S29}$$

where the scalar factor of 10^6 is introduced to convert from $\mathrm{mol\,mL^{-1}}$ (in the definition of L_C) to $\mathrm{mmol\,L^{-1}}$ (i.e., standard units for reporting sulphate concentration). Combining Eqs. S26, S28, and S29 yields the final factor

$$f_G = \rho_{\mathrm{sol}}\left(\frac{1-\phi}{\phi}\right) L_C L_G, \tag{S30}$$

which converts wt % OC respired to mM SO_4^{2-} reduced. We can therefore rewrite the proportionality in Eq. S25 as the equality

$$\mathrm{SRR}(z, S) = -f_G k_G(t) G(z). \tag{S31}$$

Substituting Eq. S3 into Eq. S31 results in the derivative form

$$\mathrm{SRR}(z, S) = w f_G \frac{\partial G(z)}{\partial z}. \tag{S32}$$

Finally, while sulphate reduction rate in sulphate-rich porewaters depends only on OC content, sulphate concentration itself can become limiting in sulphate-poor porewaters[8, 67]. Following Assumption 6, we therefore define the Monod factor as

$$\mathrm{SRR}(z, S) = R_m(z) \left(\frac{S(z)}{K_S + S(z)} \right), \tag{S33}$$

where $R_m(z)$ is the maximum (i.e., sulphate concentration-independent) rate at depth z and K_S is the concentration at which $\mathrm{SRR}(z, S = K_S) = R_m(z)/2$ (i.e., the Monod half-velocity constant), which we take to be $K_S = 1.62$ mM SO_4^{2-} following Ref.[67] (Extended Data Table 1). Using Eq. S32 as $R_m(z)$ and substituting into Eq. S33 yields

$$\mathrm{SRR}(z, S) = w f_G \frac{\partial G(z)}{\partial z} \left(\frac{S(z)}{K_S + S(z)} \right). \tag{S34}$$

Substituting Eq. S34 into Eq. S22, we finally arrive at the second-order ordinary differential equation

$$D_S \frac{\partial^2 S(z)}{\partial z^2} - w \left[\frac{\partial S(z)}{\partial z} + f_G \frac{\partial G(z)}{\partial z} \left(\frac{S(z)}{K_S + S(z)} \right) \right] = 0. \tag{S35}$$

Equation S35 defines our dimensional sulphate concentration model. This allows us to describe MSR using only $\partial G(z)/\partial z$, known or empirically constrained constants (S_0, D_S, K_S, ρ_{sol}), and measured variables that can be interpolated using global datasets (w, ϕ; see Supplementary Discussion Sec. 4) as inputs. Our model requires no free fitting parameters.

2.3 Reactive iron content

Solid-phase reactive iron (Fe_{HR}) in sediments reacts with dissolved-phase sulphide produced by MSR to form FeS and eventually pyrite[70,79]. Similar to the approach used for OC, we describe Fe_{HR} content at time t and depth z in a sediment column by the one-dimensional diffusion-advection-reaction equation (Assumption 1)[55]:

$$\frac{\partial F(t, z)}{\partial t} = D_F \frac{\partial^2 F(t, z)}{\partial z^2} - w \frac{\partial F(t, z)}{\partial z} - R_F(t), \tag{S36}$$

where $F(t, z)$ is Fe_{HR} content, D_F is Fe_{HR} diffusivity, and $R_F(t)$ is the time-dependent Fe_{HR} reduction rate (Extended Data Table 1). We again ignore sediment compaction (Assumption 2), and assume steady state (Assumption 3) such that

$$\frac{\partial F(t, z)}{\partial t} = 0, \tag{S37}$$

and neglect Fe_{HR} diffusion due to bioturbation within the sulphidic anoxic zone by setting $D_F = 0$ (Assumption 4). Equation S36 thus becomes

$$\frac{\partial F(z)}{\partial z} = -\frac{R_F(t)}{w}, \tag{S38}$$

subject to the initial condition

$$F(0) = F_0. \tag{S39}$$

We now quantify $R_F(t)$, the rate at which Fe(III)-bearing Fe_{HR} is reduced to Fe(II) by oxidation of sulphide. In contrast to OC respiration, time-dependent Fe_{HR} reduction rate laws in natural sediments have received little attention and remain largely unconstrained. Still, some studies have experimentally determined reduction rate laws and coefficients for several individual Fe_{HR} species in well-controlled laboratory settings[29, 94, 111,127]. For example, Ref.[111] found that the rate of Fe_{HR} dissolution for each species follows the reaction

$$R_{F_i}(t) = k'_{F_i}\, H^{0.5}\, F_i(t), \tag{S40}$$

where i is the Fe_{HR} species of interest [e.g., hydrous ferric oxide, HFO; lepidocrocite, $\gamma-$FeO(OH); goethite, $\alpha-$FeO(OH); haematite, Fe_2O_3; magnetite, Fe_3O_4], H is the initial sulphide concentration, $F_i(t)$ is the concentration of Fe_{HR} species i at time t, k'_{F_i} is the rate constant for Fe_{HR} species i [with units $\mathrm{yr}^{-1}\,(\mathrm{mM\,S^{-II}})^{-0.5}$; i.e., half-order with respect to H and first-order with respect to $F_i(t)$], and we use the superscript "′" to indicate rate constants for reduction relationships that depend explicitly on sulphide concentration. Analogous to OC degradation, there exist several iron minerals that are reactive towards sulphide, each dissolving at its own rate[94, 111]. We therefore consider a continuum of reactive Fe_{HR} species[29, 93], and we write the total Fe_{HR} reduction rate for natural sediments as the sum of those for individual species:

$$R_F(t) = \sum_i R_{F_i}(t). \tag{S41}$$

Substituting Eq. S40 into Eq. S41 gives

$$R_F(t) = H^{0.5} \sum_i k'_{F_i}\, F_i(t). \tag{S42}$$

Because total Fe_{HR} reduction rate is first-order with respect to total Fe_{HR} concentration, we can write

$$R_F(t) = F(t)k'_F(t), \tag{S43}$$

where $k'_F(t)$ is the bulk first-order decay coefficient at time t. Utilising the definition of total Fe_{HR}, substituting Eq. S43 into Eq. S42, and rearranging yields

$$k'_F(t) = H^{0.5} \frac{\sum_i k'_{F_i}\, F_i(t)}{\sum_i F_i(t)}, \tag{S44}$$

which describes a parallel superposition of first-order reactions[93, 122].
Because Fe_{HR} dissolution in laboratory experiments is observed to exhibit only half-order dependency on sulphide (Eq. S40), following Ref.[29], we explore the simplifying assumption that this process can be treated as independent of electron donor present; i.e., independent of sulphide concentration. In this case, Eq. S44 simplifies to

$$k'_F(t) \approx k_F(t) = \frac{\sum_i k_{F_i}\, F_i(t)}{\sum_i F_i(t)}. \tag{S45}$$

We assess the accuracy of reaction rates derived from Eq. S45 compared to those from Eq. S44 using a hypothetical $k_F(t)$ profile and three sulphide concentration scenarios that span the range of observed values. As can be seen in Supplementary Fig. 1, predicted $k'_F(t)$ values agree with prescribed $k_F(t)$ to within 30% for all sulphide scenarios. Because this disagreement is significantly smaller than the orders-of-magnitude variability expected in marine sediments, we treat Fe_{HR} reduction as independent of sulphide concentration throughout our model.
Having established that Fe_{HR} reduction can be reasonably described as a parallel superposition of first-order reactions, we now constrain the power-law regression slope and constant for this process by

combining experimental constraints and observational Fe_{HR} profiles in natural settings. First, we utilise reaction rates from Ref.[111], combined with average species-specific Fe_{HR} contents from a set of well-characterised core-top materials (Supplementary Table 1), to estimate $k_F(t)$ evolution over laboratory timescales using Eq. S44. We consider two scenarios with varying sulphide concentrations (i.e., 0.1 mM and 10 mM), chosen to represent the lower- and upper-bounds in typical natural sediments. Absolute Fe_{HR} reduction rates depend on the initial time, t_0. Here, we choose $t_0 = 60$ s, i.e., the time of first observation in the experiments of Ref.[111]; however, resulting trends are insensitive to this choice.

Second, we compile all available sedimentary Fe_{HR} content profiles from the literature[73,94,128,129]. In doing so, we exclude portions of profiles that exhibit non-steady state behaviour, which we characterise as those displaying increasing Fe_{HR} contents with depth. We then smooth retained data using an $n/3$-point moving average, where n is the profile-specific number of extracted data measurements. This moving average was chosen to effectively dampen short-term variability—which is necessary to avoid physically unrealistic negative $k_F(t)$ values—while preserving overall trends. Although such smoothing impacts data scatter and thus power-law fit uncertainty, average $k_F(t)$ trends are largely insensitive to this choice.

Using these datasets, we estimate the $k_F(t)$ power-law regression slope and coefficient. Following the approach of Ref.[30], time-dependent decay rate can be calculated as

$$k_F(t) = \frac{1}{\Delta t} \ln \frac{F(t)}{F(t + \Delta t)}, \tag{S46}$$

where $k_F(t)$ is the expressed rate coefficient and $F(t)$ is the total Fe_{HR} content at time t. By applying Eq. S46, we observe a clear power-law decrease in $k_F(t)$ for both experiments and natural sediments (Supplementary Fig. 2). Using GM regression, we derive $k_F(t)$ rate laws in two ways: (i) using exclusively sedimentary core data, resulting in $k_F(t) = (0.58 \pm 0.53)t^{(-1.05 \pm 0.10)}$, and (ii) for all available data, yielding $k_F(t) = (0.16 \pm 0.01)t^{(-0.91 \pm 0.02)}$ (uncertainties as 95% CI). Given the regression slope is near -1 in both cases—as well as the observation that a slope of -1 results naturally from several mechanistic decay-rate models[122,124]—we prescribe this value here, analogous to OC decay (see Supplementary Discussion Sec. 2.1). We thus calculate a final, combined Fe_{HR} decay-rate coefficient relationship of

$$k_F(t) = (0.17 \pm 0.04)t^{-1}, \tag{S47}$$

which we apply to our model. Although we are interested in Fe_{HR} reduction only in the sulphidic anoxic zone, the parallel first-order reaction approach derived here implicitly allows Fe_{HR} decay to persist below this zone using a less energetically favourable electron donor such as CH_4 in the absence of S^{-II} (Refs.[130,131]). This result is analogous to OC respiration, whose rate coefficient has been shown to decrease following a continuous power-law function with depth regardless of the terminal electron acceptor used (e.g., SO_4^{2-}, CH_4, etc.)[50].

By analogy to Eqs. S6-S16, we utilise the definition of t and its relationship to sedimentation rate to rewrite Eq. S47 as

$$k_F(t) = \frac{b}{\frac{b}{k_{F_0}} + \frac{z}{w}}, \tag{S48}$$

where $k_{F_0} = k_F(t_0)$ is the Fe_{HR} decay rate coefficient at the top of the sulphidic anoxic zone and $b = 0.17 \pm 0.04$ is the power-law coefficient determined above (Extended Data Table 1). However, unlike for OC, which is degraded by oxic respiration in the bioturbated zone, here we assume that Fe_{HR} does not undergo net reductive dissolution until entering the sulphidic anoxic zone. This is supported by the observation that Fe_{HR} is present in near-constant concentrations above the mixed layer depth in many settings and only begins to decrease below this depth[38,132]. We therefore explicitly set $k_{F_0} = k_{F_{sw}}$, the decay-rate coefficient at the sediment-water interface.

Finally, substituting Eqs. S43 and S48 into Eq. S38, we arrive at the first-order ordinary differential equation

$$\frac{\partial F(z)}{\partial z} = \left(\frac{-b}{w\frac{b}{k_{F_0}} + z} \right) F(z) \tag{S49}$$

which has the analytical solution

$$F(z) = F_0 \left(\frac{bw}{bw + k_{F_0} z} \right)^b . \tag{S50}$$

Like for k_{F_0}, we explicitly set $F_0 = F_{sw}$, the Fe_{HR} content at the sediment-water interface. Equations S49-S50 define our dimensional Fe_{HR} decay model. Like for OC respiration, this allows us to describe Fe_{HR} decay using only empirically constrained constants (b) and measured variables that can be interpolated using global datasets (w, F_{sw}, $k_{F_{sw}}$; see Supplementary Discussion Sec. 4) as inputs. Our model requires no free fitting parameters.

2.4 Sulphide concentration

As for other species, we describe sulphide concentration at time t and depth z in a sediment column by the one-dimensional diffusion-advection-reaction equation[55] (Assumptions 1-2):

$$\frac{\partial H(t,z)}{\partial t} = D_H \frac{\partial^2 H(t,z)}{\partial z^2} - w \frac{\partial H(t,z)}{\partial z} + \mathrm{SRR}(z,S) - \mathrm{Ox}(z,H), \tag{S51}$$

where $H(t,z)$ is the sum of dissolved sulphide species (H_2S, HS^-, S^{2-}, collectively S^{-II}) concentrations, D_H is sulphide diffusivity[95], and $\mathrm{Ox}(z,H)$ is the rate of net sulphide oxidation by Fe_{HR} to form pyrite at depth z and sulphide concentration H (Extended Data Table 1). As before, we assume steady state (Assumption 3) such that

$$\frac{\partial H(t,z)}{\partial t} = 0. \tag{S52}$$

Equation S51 then becomes

$$D_H \frac{\partial^2 H(z)}{\partial z^2} - w \frac{\partial H(z)}{\partial z} + \mathrm{SRR}(z,S) - \mathrm{Ox}(z,H) = 0, \tag{S53}$$

subject to the initial condition

$$H(0) = H_0, \tag{S54}$$

where $H_0 = 0$ mM in the fully oxygenated modern ocean (but see Supplementary Discussion Sec. 8 for discussion on model performance in euxinic ocean conditions), and the boundary condition

$$\lim_{z \to \infty} \frac{\partial H(z)}{\partial z} = 0. \tag{S55}$$

Like for sulphate, Eq. S55 states that, independent of the final sulphide concentration, $\partial H / \partial z \to 0$ far below the sulphate reduction zone, i.e., where production and consumption no longer occur and diffusive gradients become insignificant.
To quantify $\mathrm{Ox}(z,H)$, we assume that all Fe_{HR} reduction is coupled to S^{-II} oxidation following the generalised stoichiometry[81]

$$\mathrm{FeO(OH)} + 2\,\mathrm{H_2S} \longrightarrow \mathrm{FeS_2} + \frac{1}{2}\,\mathrm{H_2} + 2\,\mathrm{H_2O}. \tag{S56}$$

Following Assumption 7 leads to

$$\mathrm{Ox}(z,H) \propto k_F(t) F(z) \tag{S57}$$

where the proportionality arises from the need for a scaling term to convert from solid-phase wt % Fe_{HR} in sediment particles to dissolved-phase mM S^{-II} in porewaters. Like for f_G, this is achieved in three steps:

first, we convert wt % Fe_{HR} to mol Fe_{HR} $(g\,sol)^{-1}$ using the factor

$$L_I = \frac{1}{100}\frac{1}{55.49}, \tag{S58}$$

where 55.49 $g\,mol^{-1}$ is the molar mass of iron. Second, we convert mol Fe_{HR} $(g\,sol)^{-1}$ to mol Fe_{HR} $(V_{void})^{-1}$ using Eq. S28. Third, to convert mol Fe_{HR} reduced to mol S^{-II} oxidised, we use the stoichiometry defined in Eq. S56 and define the conversion factor

$$L_F = 2 \times 10^6, \tag{S59}$$

where the scalar factor 10^6 is again introduced to convert from mol mL^{-1} in the definition of L_I to mmol L^{-1} (i.e., standard units for reporting sulphide concentrations). Combining Eqs. S28, S58, and S59 yields the final factor

$$f_F = \rho_{sol}\left(\frac{1-\phi}{\phi}\right) L_I L_F, \tag{S60}$$

which converts wt % Fe_{HR} reduced to mM S^{-II} oxidised (Extended Data Table 1). We can thus rewrite the proportionality in Eq. S57 as the equality

$$\mathrm{Ox}(z, H) = f_F k_F(t) F(z). \tag{S61}$$

Substituting Eq. S38 into Eq. S61 results in the derivative form

$$\mathrm{Ox}(z, H) = -w f_F \frac{\partial F(z)}{\partial z}. \tag{S62}$$

Equation S62 is consistent with data from Refs.[69,70], which suggests that pyrite formation rate—and therefore sulphide oxidation rate (Assumption 7)—is first-order with respect to Fe_{HR} concentration. Similar to SRR, Refs.[69,70] additionally show that pyrite formation rate follows Monod kinetics with respect to sulphide concentration. Following Assumption 6 and the generalised stoichiometry presented in Eq. S56, we therefore define the Monod factor for sulphide oxidation as

$$\mathrm{Ox}(z, H) = R_m(z)\left(\frac{H(z)}{K_H + H(z)}\right), \tag{S63}$$

where $R_m(z)$ is again the maximum (i.e., sulphide concentration-independent) rate at depth z and K_H is the Monod half-velocity constant. Following the approach of Ref.[67], here we determine the Monod half-velocity constant for sulphide oxidation using data from Ref.[70] by regressing the reciprocals of sulphide concentration and pyrite formation rate (Supplementary Fig. 3). In doing so, we estimate $K_H = 1.78 \pm 0.33$ (uncertainty as regression 95% CI), which we utilise in our model (Extended Data Table 1). Taking Eq. S62 as $R_m(z)$ and substituting into Eq. S63 yields

$$\mathrm{Ox}(z, H) = -w f_F \frac{\partial F(z)}{\partial z}\left(\frac{H(z)}{K_H + H(z)}\right). \tag{S64}$$

Substituting Eqs. S34 and S64 into Eq. S53, we finally arrive at the second-order ordinary differential equation

$$D_H \frac{\partial^2 H(z)}{\partial z^2} - w\left[\frac{\partial H(z)}{\partial z} + f_G \frac{\partial G(z)}{\partial z}\left(\frac{S(z)}{K_S + S(z)}\right) - f_F \frac{\partial F(z)}{\partial z}\left(\frac{H(z)}{K_H + H(z)}\right)\right] = 0. \tag{S65}$$

Equation S65 defines our dimensional sulphide concentration model. Like for sulphate, this allows us to describe S^{-II} oxidation as a function of sedimentation rate using only $\partial G(z)\partial z$, $\partial F(z)/\partial z$, known or empirically constrained constants (H_0, D_H, K_H, K_S, ρ_{sol}), and measured variables that can be interpolated using global datasets (w, ϕ; see Supplementary Discussion Sec. 4). Our model requires no free fitting parameters.

2.5 Pyrite content

The process of pyrite formation is complex, involving various sulphur intermediate species and several sequential reaction steps[76,79–81,111]. Because of this complexity, two mechanisms for pyrite formation have been proposed—the so-called "S^{-II}" and "polysulphide" pathways. Both pathways require aqueous FeS as an intermediate reactant, which is itself formed via a two-step reaction in which Fe_{HR} is reduced to dissolved Fe^{2+} by S^{-II} and Fe^{2+} subsequently reacts with S^{-II} to produce FeS. Following the S^{-II} pathway, FeS reacts with S^{-II} to generate FeS_2[81]. In contrast, the polysulphide pathway predicts that FeS reacts with polysulphides (e.g., greigite, Fe_3S_4) to generate FeS_2. Although most kinetic studies of pyrite formation to date have focused on the $Fe^{2+} + S^{-II}$ step specific to the S^{-II} pathway[80,81], some kinetic constraints for the $Fe_{HR} + S^{-II}$ step common to both pathways do exist[133,69,70]; we utilised such constraints above to estimate S^{-II} oxidation kinetics (see Supplementary Discussion Sec. 2.4). Here, we assume the $Fe_{HR} + S^{-II}$ reaction common to both pathways represents the rate-limiting step of pyrite formation[133,69,70]. The treatment implicitly states that all decayed Fe_{HR} contributes to pyrite formation when S^{-II} is available, a corollary of Assumptions 3 and 7, which preclude the accumulation of intermediate sulphur species. We therefore explicitly set the pyrite formation rate as equal to the sulphide oxidation rate, $\mathrm{Ox}(z, H)$. In making this assumption, our model treats pyrite formation rate as insensitive to formation pathway (i.e., S^{-II} or polysulphide pathway).

Similar to all other species, we describe pyrite content at time t and depth z in a sediment column by the one-dimensional diffusion-advection-reaction equation[55]:

$$\frac{\partial P(t,z)}{\partial t} = D_P \frac{\partial^2 P(t,z)}{\partial z^2} - w \frac{\partial P(t,z)}{\partial z} + \frac{1}{f_P} \mathrm{Ox}(z,H), \tag{S66}$$

where $P(t, z)$ is pyrite content, D_P is pyrite diffusivity, and f_P is a scaling factor to convert from wt % FeS_2 to mM S^{-II}. Like for f_F, first we convert wt % FeS_2 to mol FeS_2 $(\mathrm{g\,sol})^{-1}$ using the factor

$$L_P = \frac{1}{100} \frac{1}{119.98}, \tag{S67}$$

where $119.98\,\mathrm{g\,mol^{-1}}$ is the molar mass of pyrite. Second, we convert mol FeS_2 $(\mathrm{g\,sol})^{-1}$ to mol FeS_2 $(\mathrm{V_{void}})^{-1}$ using Eq. S28. Third, to convert mol FeS_2 produced to mol S^{-II} oxidised, we use the stoichiometry defined in Eq. S56 and define the conversion factor

$$L_S = 2 \times 10^6, \tag{S68}$$

where the scalar factor 10^6 is again introduced to convert from $\mathrm{mol\,mL^{-1}}$ in the definition of L_P to $\mathrm{mmol\,L^{-1}}$ (i.e., standard units for reporting sulphide concentrations). Combining Eqs. S28, S67, and S68 yields the final factor

$$f_P = \rho_{\mathrm{sol}} \left(\frac{1-\phi}{\phi} \right) L_P L_S, \tag{S69}$$

which converts wt % FeS_2 produced to mM S^{-II} oxidised (Extended Data Table 1). As above, we assume steady state such that

$$\frac{\partial P(t,z)}{\partial z} = 0, \tag{S70}$$

and we ignore sediment compaction. As for other solid-phase species, we ignore bioturbation within the sulphidic zone by setting $D_P = 0$, which yields the governing equation

$$\frac{\partial P(z)}{\partial z} = \frac{1}{w f_P} \mathrm{Ox}(z,H), \tag{S71}$$

subject to the initial condition

$$P(0) = P_0, \tag{S72}$$

where $P_0 = 0$ since pyrite deposition on the sediment-water interface is expected to be negligible in the well-oxygenated modern ocean (but see Supplementary Discussion Sec. 8 for discussion on model performance in euxinic ocean conditions). Substituting Eq. S62 into Eq. S71 yields

$$\frac{\partial P(z)}{\partial z} = -\frac{f_F}{f_P}\frac{\partial F(z)}{\partial z}\left(\frac{H(z)}{K_H + H(z)}\right), \tag{S73}$$

where $\partial F(z)/\partial z$ follows Eq. S49 and f_F/f_P describes the ratio of conversion factors between solid-phase Fe_{HR} and pyrite to dissolved S^{-II}. This reduces to the ratio of molar masses such that

$$\begin{aligned}\frac{f_F}{f_P} &= \frac{55.84\,\mathrm{g\,Fe\,mol^{-1}}}{119.98\,\mathrm{g\,FeS_2\,mol^{-1}}} \\ &= 0.47\,(\mathrm{g\,FeS_2})\,(\mathrm{g\,Fe})^{-1}.\end{aligned} \tag{S74}$$

Equation S73 defines our dimensional pyrite formation model. This allows us to describe pyrite production as a function of sedimentation rate using the same empirically constrained constants and measured variables interpolated using global datasets (Supplementary Discussion Sec. 4) as described for sulphide concentration, above. Our model requires no free fitting parameters.

2.6 Isotopic compositions

Nomenclature

Sulphur contains four stable isotopes—^{32}S, ^{33}S, ^{34}S, and ^{36}S—with ^{32}S and ^{34}S respectively comprising 95.02 % and 4.21 % of the total sulphur pool, S_{total}. We thus assume here for simplicity that

$$S_{total} = {}^{32}S + {}^{34}S. \tag{S75}$$

That is, we ignore minor isotopes. We additionally define the sulphur-isotope ratio for any compound A as

$${}^{34}R_A = \left(\frac{^{34}S}{^{32}S}\right)_A. \tag{S76}$$

Isotopic compositions are often written in "delta" notation as

$$\delta^{34}S_A = \frac{^{34}R_A}{^{34}R_{VCDT}} - 1, \tag{S77}$$

where VCDT is the Vienna Canyon Diablo Troilite international standard [82]; results are reported in units of "permil" by multiplying Eq. S77 by 1000 ‰. Furthermore, the sulphur-isotope fractionation factor for any kinetic or equilibrium chemical process between product compound A and reactant compound B is defined as

$${}^{34}\alpha_{A/B} = \frac{^{34}R_A}{^{34}R_B}. \tag{S78}$$

Finally, analogous to "delta" notation, fractionation factors can also be written in "epsilon" notation as

$${}^{34}\varepsilon_{A/B} = {}^{34}\alpha_{A/B} - 1, \tag{S79}$$

which is similarly reported in units of permil by multiplying Eq. S79 by 1000 ‰. Following the nomenclature defined above (Extended Data Table 1), we explicitly write the total concentration or content of each sulphur-containing species of interest as the sum of individual isotopologues:

$$S(z) = \left[{}^{32}SO_4^{2-}\right](z) + \left[{}^{34}SO_4^{2-}\right](z), \tag{S80}$$

$$H(z) = \left[{}^{32}S^{-}II\right](z) + \left[{}^{34}S^{-}II\right](z), \tag{S81}$$

and

$$P(z) = \left[\mathrm{Fe}^{32}\mathrm{S}^{32}\mathrm{S}\right](z) + \left[\mathrm{Fe}^{32}\mathrm{S}^{34}\mathrm{S}\right](z), \tag{S82}$$

where square brackets indicate concentrations or contents and we ignore the doubly-substituted FeS_2 isotopologue $\mathrm{Fe}^{34}\mathrm{S}^{34}\mathrm{S}$ since its content is negligible. We can rewrite Eqs. S80-S82 as

$$S(z) = {}^{32}S(z) + {}^{34}S(z), \tag{S83}$$

$$H(z) = {}^{32}H(z) + {}^{34}H(z), \tag{S84}$$

and

$$P(z) = {}^{32}P(z) + {}^{34}P(z), \tag{S85}$$

where right-hand terms represent isotopologue-specific concentrations or contents of each species of interest at depth z. We adopt the notation of Eqs. S83-S85 in subsequent isotopic composition derivations.

Sulphate isotopic composition

To determine the isotopic composition of sulphate at any depth z, we independently solve our dimensional sulphate concentration model, Eq. S35, for each isotopologue, ${}^{32}\mathrm{SO}_4^{2-}$ and ${}^{34}\mathrm{SO}_4^{2-}$. Following Assumption 8, we treat MSR as the only fractionating process and allow this to always operate at its temperature-dependent equilibrium fractionation factor [19], which is in the range

$${}^{34}\alpha_{\mathrm{S^{-II}}/\mathrm{SO_4^{2-}}} \in (0.926, 0.935) \tag{S86}$$

for Earth-surface temperatures of interest here (i.e., 0 to 25 °C) [31]. Thus, in order to solve Eq. S35 for each isotopologue, we must first determine isotopologue-specific sulphate reduction rates. These can be written as

$${}^{32}\mathrm{SRR}(z,S) = \left(\frac{{}^{32}S_r(z)}{S_r(z)}\right)\mathrm{SRR}(z,S) \tag{S87}$$

and

$${}^{34}\mathrm{SRR}(z,S) = \left(\frac{{}^{34}S_r(z)}{S_r(z)}\right)\mathrm{SRR}(z,S), \tag{S88}$$

where $\mathrm{SRR}(z,S)$ is defined in Eq. S34 and subscript "r" indicates only the portion of the total sulphate pool that is instantaneously being reduced (equivalent to the concentration of $\mathrm{S^{-II}}$ that is instantaneously generated) at depth z. Importantly, the sulphur-isotope ratios of $S(z)$ and $S_r(z)$ are not equal but are related by the MSR fractionation factor such that

$$\frac{{}^{34}S_r(z)}{{}^{32}S_r(z)} = {}^{34}\alpha_{\mathrm{S^{-II}}/\mathrm{SO_4^{2-}}} \times \frac{{}^{34}S(z)}{{}^{32}S(z)}. \tag{S89}$$

Substituting Eqs. S83 and S89 into Eqs. S87-S88 yields

$${}^{32}\mathrm{SRR}(z,S) = \left(\frac{{}^{32}S(z)}{{}^{34}\alpha_{\mathrm{S^{-II}}/\mathrm{SO_4^{2-}}} \times {}^{34}S(z) + {}^{32}S(z)}\right)\mathrm{SRR}(z,S) \tag{S90}$$

and

$${}^{34}\mathrm{SRR}(z,S) = \left(\frac{{}^{34}\alpha_{\mathrm{S^{-II}}/\mathrm{SO_4^{2-}}} \times {}^{34}S(z)}{{}^{34}\alpha_{\mathrm{S^{-II}}/\mathrm{SO_4^{2-}}} \times {}^{34}S(z) + {}^{32}S(z)}\right)\mathrm{SRR}(z,S). \tag{S91}$$

We then utilise Eqs. S34, S90, and S91 to rewrite our dimensional sulphate concentration model, Eq. S35, for each isotopologue as

$$D_S \frac{\partial^2 \left[{}^{32}S(z)\right]}{\partial z^2} - w \left\{ \frac{\partial \left[{}^{32}S(z)\right]}{\partial z} - f_G \frac{\partial G(z)}{\partial z} \left(\frac{{}^{32}S(z)}{{}^{34}\alpha_{\mathrm{S^{-II}/SO_4^{2-}}} \times {}^{34}S(z) + {}^{32}S(z)} \right) \left(\frac{S(z)}{K_S + S(z)} \right) \right\} = 0 \quad \text{(S92)}$$

and

$$D_S \frac{\partial^2 \left[{}^{34}S(z)\right]}{\partial z^2} - w \left\{ \frac{\partial \left[{}^{34}S(z)\right]}{\partial z} - f_G \frac{\partial G(z)}{\partial z} \left(\frac{{}^{34}\alpha_{\mathrm{S^{-II}/SO_4^{2-}}} \times {}^{34}S(z)}{{}^{34}\alpha_{\mathrm{S^{-II}/SO_4^{2-}}} \times {}^{34}S(z) + {}^{32}S(z)} \right) \left(\frac{S(z)}{K_S + S(z)} \right) \right\} = 0, \quad \text{(S93)}$$

subject to the initial conditions

$${}^{32}S(0) = \frac{1}{1 + {}^{34}\mathrm{R}_{S_0}} S_0 \quad \text{(S94)}$$

and

$${}^{34}S(0) = \frac{{}^{34}\mathrm{R}_{S_0}}{1 + {}^{34}\mathrm{R}_{S_0}} S_0, \quad \text{(S95)}$$

where ${}^{34}\mathrm{R}_{S_0}$ is the sulphur-isotope ratio of seawater sulphate, and the boundary conditions

$$\lim_{z \to \infty} \frac{\partial \left[{}^{32}S(z)\right]}{\partial z} = 0 \quad \text{(S96)}$$

and

$$\lim_{z \to \infty} \frac{\partial \left[{}^{34}S(z)\right]}{\partial z} = 0. \quad \text{(S97)}$$

Equations S83, S92, and S93 define our isotopologue-specific dimensional sulphate model. Finally, we convert resulting ${}^{32}S(z)$ and ${}^{34}S(z)$ values to delta notation following

$$\delta^{34}\mathrm{S}_{S(z)} = \frac{{}^{34}S(z)}{{}^{32}S(z)} \left(\frac{1}{{}^{34}\mathrm{R}_{\mathrm{VCDT}}} \right) - 1, \quad \text{(S98)}$$

where ${}^{34}\mathrm{R}_{\mathrm{VCDT}} = 0.044162$[82], and we have utilised the relationship

$${}^{34}\mathrm{R}_{S_0} = \left(\delta^{34}\mathrm{S}_{S_0} + 1\right) {}^{34}\mathrm{R}_{\mathrm{VCDT}}, \quad \text{(S99)}$$

where $\delta^{34}\mathrm{S}_{S_0} = 21$ ‰ in the modern ocean[7].

Sulphide isotopic composition

Similar to sulphate, we determine the isotopic composition of sulphide at any depth z by independently solving our dimensional sulphide concentration model, Eq. S65, for each isotopologue, ${}^{32}\mathrm{S}^{-\mathrm{II}}$ and ${}^{34}\mathrm{S}^{-\mathrm{II}}$. To do so, we must first determine isotopologue-specific sulphide oxidation rates. Following Assumption 8, we ignore any sulphur-isotope fractionation between $\mathrm{S}^{-\mathrm{II}}$ and $\mathrm{FeS_2}$ during the multi-step oxidation and pyrite formation process. Utilising the definition of $H(z)$ in Eq. S84, isotopologue-specific oxidation rates can thus be written as

$${}^{32}\mathrm{Ox}(z, H) = \left(\frac{{}^{32}H(z)}{{}^{32}H(z) + {}^{34}H(z)} \right) \mathrm{Ox}(z, H) \quad \text{(S100)}$$

and

$$ {}^{34}\mathrm{Ox}(z,H) = \left(\frac{{}^{34}H(z)}{{}^{32}H(z) + {}^{34}H(z)} \right) \mathrm{Ox}(z,H), \tag{S101} $$

where $\mathrm{Ox}(z,H)$ is defined in Eq. S64. We then utilise Eqs. S64, S90, S91, S100, and S101 to rewrite our dimensional sulphide concentration model, Eq. S65, for each isotopologue as

$$ \begin{aligned} D_H \frac{\partial^2 \left[{}^{32}H(z)\right]}{\partial z^2} - w \Bigg\{ \frac{\partial \left[{}^{32}H(z)\right]}{\partial z} + f_G \frac{\partial G(z)}{\partial z} \left(\frac{{}^{32}S(z)}{{}^{34}\alpha_{\mathrm{S^{-II}/SO_4^{2-}}} \times {}^{34}S(z) + {}^{32}S(z)} \right) \left(\frac{S(z)}{K_S + S(z)} \right) \\ - f_F \frac{\partial F(z)}{\partial z} \left(\frac{{}^{32}H(z)}{{}^{32}H(z) + {}^{34}H(z)} \right) \left(\frac{H(z)}{K_H + H(z)} \right) \Bigg\} = 0 \end{aligned} \tag{S102} $$

and

$$ \begin{aligned} D_H \frac{\partial^2 \left[{}^{34}H(z)\right]}{\partial z^2} - w \Bigg\{ \frac{\partial \left[{}^{34}H(z)\right]}{\partial z} + f_G \frac{\partial G(z)}{\partial z} \left(\frac{{}^{34}\alpha_{\mathrm{S^{-II}/SO_4^{2-}}} \times {}^{34}S(z)}{{}^{34}\alpha_{\mathrm{S^{-II}/SO_4^{2-}}} \times {}^{34}S(z) + {}^{32}S(z)} \right) \left(\frac{S(z)}{K_S + S(z)} \right) \\ - f_F \frac{\partial F(z)}{\partial z} \left(\frac{{}^{34}H(z)}{{}^{32}H(z) + {}^{34}H(z)} \right) \left(\frac{H(z)}{K_H + H(z)} \right) \Bigg\} = 0, \end{aligned} \tag{S103} $$

subject to the initial conditions

$$ {}^{32}H(0) = \frac{1}{1 + {}^{34}\mathrm{R}_{H_0}} H_0 \tag{S104} $$

and

$$ {}^{34}H(0) = \frac{{}^{34}\mathrm{R}_{H_0}}{1 + {}^{34}\mathrm{R}_{H_0}} H_0, \tag{S105} $$

where ${}^{34}\mathrm{R}_{H_0}$ is the sulphur-isotope ratio of seawater $\mathrm{S^{-II}}$ (only applicable when $H_0 > 0$; see Supplementary Discussion Sec. 8), and the boundary conditions

$$ \lim_{z \to \infty} \frac{\partial \left[{}^{32}H(z)\right]}{\partial z} = 0 \tag{S106} $$

and

$$ \lim_{z \to \infty} \frac{\partial \left[{}^{34}H(z)\right]}{\partial z} = 0. \tag{S107} $$

Equations S83, S84, S102, and S103 define our isotopologue-specific dimensional sulphide model. Finally, like for sulphate, we convert resulting ${}^{32}H(z)$ and ${}^{34}H(z)$ values to delta notation following

$$ \delta^{34}\mathrm{S}_{H(z)} = \frac{{}^{34}H(z)}{{}^{32}H(z)} \left(\frac{1}{{}^{34}\mathrm{R_{VCDT}}} \right) - 1. \tag{S108} $$

Pyrite isotopic composition

Similar to sulphate and sulphide, we determine the isotopic composition of product pyrite at any depth z by independently solving our dimensional pyrite content model for each major isotopologue, $\mathrm{Fe^{32}S^{32}S}$ and $\mathrm{Fe^{32}S^{34}S}$. Because we ignore any sulphur-isotope fractionation between $\mathrm{S^{-II}}$ and $\mathrm{FeS_2}$ during pyrite formation (Assumption 8), we achieve this by substituting Eqs. S64, S100, and S101 into Eq. S73 for each

isotopologue as

$$\frac{\partial\left[{}^{32}P(z)\right]}{\partial z} = -\frac{f_F}{f_P}\frac{\partial F(z)}{\partial z}\left(\frac{{}^{32}H(z)}{{}^{32}H(z)+{}^{34}H(z)}\right)\left(\frac{H(z)}{K_H+H(z)}\right) \tag{S109}$$

and

$$\frac{\partial\left[{}^{34}P(z)\right]}{\partial z} = -\frac{f_F}{f_P}\frac{\partial F(z)}{\partial z}\left(\frac{{}^{34}H(z)}{{}^{32}H(z)+{}^{34}H(z)}\right)\left(\frac{H(z)}{K_H+H(z)}\right), \tag{S110}$$

subject to the initial conditions

$$^{32}P(0) = \frac{1}{1+{}^{34}\mathrm{R}_{P_0}}P_0 \tag{S111}$$

and

$$^{34}P(0) = \frac{{}^{34}\mathrm{R}_{P_0}}{1+{}^{34}\mathrm{R}_{P_0}}P_0, \tag{S112}$$

where ${}^{34}\mathrm{R}_{P_0}$ is the sulphur-isotope ratio of pyrite deposited on the sediment-water interface. Because deposition is expected to be negligible in the well-oxygenated modern ocean, we set ${}^{32}P(0) = {}^{34}P(0) = 0$ for most model solutions (but see Supplementary Discussion Sec. 8 for discussion on model performance in euxinic ocean conditions).
Equations S109-S110 define our isotopologue-specific dimensional pyrite model. Finally, like for previous species, we convert resulting ${}^{32}P(z)$ and ${}^{34}P(z)$ values to delta notation following

$$\delta^{34}\mathrm{S}_{P(z)} = \frac{{}^{34}P(z)}{{}^{32}P(z)}\left(\frac{1}{{}^{34}\mathrm{R}_{\mathrm{VCDT}}}\right) - 1, \tag{S113}$$

which describes the final output of our dimensional model.

3 Non-dimensional model derivation

It is reasonable to suggest that sulphate reduction and subsequent pyrite formation do not depend directly on observable properties but rather on non-dimensional property ratios, here called "natural variables" [67]. For example, increasing OC content while proportionally decreasing its reactivity would lead to no change in MSR and sulphate concentration profiles. Similarly, increasing sedimentation rate alone would decrease the potential for sulphate resupply (i.e., closed-system conditions), but simultaneously increasing sulphate diffusivity would counter this effect and restore the original concentration profile. Following this logic, we extract the natural variables that control concentrations/contents and isotopic compositions of all species considered here by non-dimensionalising our governing equations. This approach expands upon that introduced by Ref. [67], which itself is derived from "open-system" vs. "closed-system" concepts described in Refs. [134,135].
First, we re-scale depth to be a function of sulphate diffusivity and OC reactivity as

$$\zeta = \sqrt{\frac{k_{G_0}}{D_S}}z. \tag{S114}$$

Equation S114 defines the degree to which sulphate in a parcel of sediment at a given depth is connected to the overlying water, often conceptualised in terms of "open-system" vs. "closed-system" behaviour [135]. That is, if ζ is small for a given z, then sulphate resupply by diffusion dominates over sulphate consumption by MSR, and porewater can be considered "open" to exchange with overlying water. In contrast, if ζ is large for a given z, then downward-diffusing sulphate is largely consumed by MSR prior to reaching depth z, and porewater can be considered "closed" to exchange with overlying water. Importantly, in all natural sediments considered here, OC reactivity never exceeds diffusivity (i.e., $D_S \gg k_{G_0}$ always). Put

differently, non-dimensional depth ζ is always smaller than the corresponding dimensional depth z by an amount that is proportional to the relative importance of consumption by MSR vs. resupply by diffusion. Next, we define a modified version of a Damköhler number, a family of non-dimensional numbers that relate the relative importance of advection, diffusion, and reaction in controlling chemical concentrations[67]. Specifically, we let

$$\mathrm{Da}^* = \frac{\sqrt{k_{G_0} D_S}}{w}, \tag{S115}$$

where the superscript "*" indicates that this is a modified, rather than traditional, Damköhler number. It can nevertheless be expressed in terms of traditional Damköhler numbers as

$$\mathrm{Da}^* = \frac{\left(\frac{k_{G_0} L}{w}\right)}{\sqrt{\frac{k_{G_0} L^2}{D_S}}} = \frac{\mathrm{Da_I}}{\sqrt{\mathrm{Da_{II}}}}, \tag{S116}$$

where L is any characteristic length scale and $\mathrm{Da_I}$ and $\mathrm{Da_{II}}$ are traditional Damköhler numbers that describe the relative importance of reaction vs. advection and reaction vs. diffusion, respectively[136] (note that Da^* defined here is identical to α defined in Ref.[67]). Furthermore, comparing Eqs. S114 and S116, it can be seen that ζ is equal to $\mathrm{Da_{II}}$ for the characteristic length scale $L = z$. However, unlike for ζ, Assumption 2 allows us to describe each profile with a single, characteristic Da^* value. Conceptually, Eq. S115 states that sulphate transport in sediment profiles with small Da^* values is dominated by downward advection by sedimentation whereas transport in profiles with large Da^* values is dominated by consumption and diffusive resupply. That is, in the limit of $\mathrm{Da}^* \to 0$, sulphate reduction is negligible, leading to no diffusive gradient and thus purely passive transport of all sulphate to depth by sedimentation. In contrast, in the limit of $\mathrm{Da}^* \to \infty$, sedimentation is negligible, and the only downward transport of sulphate occurs by diffusion with a gradient that depends on OC respiration rate—and thus sulphate reduction rate. With these two natural variables, we proceed to non-dimensionalise concentrations/contents for all species of interest (see Extended Data Table 2 for definitions and descriptions of all non-dimensional variables).

3.1 Organic carbon content

First, we define non-dimensional OC content as

$$\Gamma = \frac{f_G G}{S_0}. \tag{S117}$$

Substituting Eqs. S114-S115 and S117 into Eq. S18 yields

$$\frac{\partial \Gamma(\zeta)}{\partial \zeta} = \left(\frac{-\mathrm{Da}^* a}{a + \mathrm{Da}^* \zeta}\right) \Gamma(\zeta), \tag{S118}$$

where we have additionally utilised the change-of-variables relationship

$$\frac{\partial \zeta}{\partial z} = \sqrt{\frac{k_{G_0}}{D_S}}, \tag{S119}$$

derived as the derivative of Eq. S114. Equation S118 is subject to the initial condition

$$\Gamma(0) = \Gamma_0, \tag{S120}$$

and has the analytical solution

$$\Gamma(\zeta) = \Gamma_0 \left(\frac{a}{a + \mathrm{Da}^* \zeta}\right)^a. \tag{S121}$$

Equations S118 and S121 define our non-dimensional OC respiration model.

3.2 Sulphate concentration

We then define non-dimensional sulphate concentration as

$$\Sigma = \frac{S}{S_0}, \tag{S122}$$

and we similarly non-dimensionalise the sulphate Monod coefficient as

$$\kappa_\Sigma = \frac{K_S}{S_0}. \tag{S123}$$

Substituting Eqs. S114-S115, S117, S119, and S122-S123 into Eq. S35 yields

$$\text{Da}^* \frac{\partial^2 \Sigma(\zeta)}{\partial \zeta^2} - \frac{\partial \Sigma(\zeta)}{\partial \zeta} + \frac{\partial \Gamma(\zeta)}{\partial \zeta} \left(\frac{\Sigma(\zeta)}{\kappa_\Sigma + \Sigma(\zeta)} \right) = 0, \tag{S124}$$

subject to the initial condition

$$\Sigma_0 = 1 \tag{S125}$$

and the boundary condition

$$\lim_{\zeta \to \infty} \frac{\partial \Sigma(\zeta)}{\partial \zeta} = 0. \tag{S126}$$

Equation S124 defines our non-dimensional sulphate concentration model.

3.3 Reactive iron content

Similar to OC content, we first define non-dimensional Fe_{HR} content as

$$\Psi = \frac{f_F F}{S_0}, \tag{S127}$$

and we additionally define the ratio of initial OC and Fe_{HR} reactivities as

$$\chi = \frac{k_{F_0}}{k_{G_0}}. \tag{S128}$$

Substituting S114-S115, S119, and S127-S128 into Eq. S49 thus yields

$$\frac{\partial \Psi(\zeta)}{\partial \zeta} = \left(\frac{-\chi \text{Da}^* b}{b + \chi \text{Da}^* \zeta} \right) \Psi(\zeta), \tag{S129}$$

subject to the initial condition

$$\Psi(0) = \Psi_0, \tag{S130}$$

which has the analytical solution

$$\Psi(\zeta) = \Psi_0 \left(\frac{b}{b + \chi \text{Da}^* \zeta} \right)^b. \tag{S131}$$

Equations S129 and S131 define our non-dimensional Fe_{HR} decay model.

3.4 Sulphide concentration

Like for sulphate, we define non-dimensional sulphide concentration as

$$\eta = \frac{H}{S_0} \tag{S132}$$

and we non-dimensionalise the sulphide Monod coefficient as

$$\kappa_\eta = \frac{K_H}{S_0}. \tag{S133}$$

We further define the ratio of sulphide and sulphate diffusivities as

$$\Delta = \frac{D_H}{D_S}. \tag{S134}$$

Substituting Eqs. S114-S115, S117, S119, S122-S123, S127, and S132-S134 into Eq. S65 thus yields

$$\Delta \mathrm{Da}^* \frac{\partial^2 \eta(\zeta)}{\partial \zeta^2} - \frac{\partial \eta(\zeta)}{\partial \zeta} - \frac{\partial \Gamma(\zeta)}{\partial \zeta}\left(\frac{\Sigma(\zeta)}{\kappa_\Sigma + \Sigma(\zeta)}\right) + \frac{\partial \Psi(\zeta)}{\partial \zeta}\left(\frac{\eta(\zeta)}{\kappa_\eta + \eta(\zeta)}\right) = 0, \tag{S135}$$

subject to the initial condition

$$\eta(0) = \eta_0, \tag{S136}$$

where $\eta_0 = 0$ in the fully oxygenated modern ocean (but see Supplementary Discussion Sec. 8 for discussion on model performance in euxinic ocean conditions), and the boundary condition

$$\lim_{\zeta \to \infty} \frac{\partial \eta(\zeta)}{\partial \zeta} = 0. \tag{S137}$$

Equation S135 defines our non-dimensional sulphide concentration model.

3.5 Pyrite content

Similar to OC and $\mathrm{Fe_{HR}}$ contents, we first define non-dimensional pyrite content as

$$\Pi = \frac{f_p P}{S_0}. \tag{S138}$$

Substituting Eqs. S119, S132-S133, S127, and S138 into Eq. S73 yields

$$\frac{\partial \Pi(\zeta)}{\partial \zeta} = -\frac{\partial \Psi(\zeta)}{\partial \zeta}\left(\frac{\eta(\zeta)}{\kappa_\eta + \eta(\zeta)}\right), \tag{S139}$$

subject to the initial condition

$$\Pi(0) = \Pi_0, \tag{S140}$$

where $\Pi_0 = 0$ since pyrite deposition on the sediment-water interface is expected to be negligible in the well-oxygenated modern ocean (but see Supplementary Discussion Sec. 8 for discussion on model performance in euxinic ocean conditions). Equation S139 defines our non-dimensional pyrite content model.

3.6 Isotopic compositions

Like for dimensional concentrations or contents, we can write the total concentration or content of each non-dimensional sulphur-containing species as the sum of individual isotopologues by re-scaling Eqs.

S83-S85 as

$$\Sigma(\zeta) = {}^{32}\Sigma(\zeta) + {}^{34}\Sigma(\zeta), \tag{S141}$$
$$\eta(\zeta) = {}^{32}\eta(\zeta) + {}^{34}\eta(\zeta), \tag{S142}$$

and

$$\Pi(\zeta) = {}^{32}\Pi(\zeta) + {}^{34}\Pi(\zeta), \tag{S143}$$

where right-hand terms represent isotopologue-specific non-dimensional concentrations or contents at non-dimensional depth ζ. We adopt the notation of Eqs. S141-S143 in subsequent isotopic composition derivations.

Sulphate isotopic composition

Substituting Eqs. S114-S115, S117, S119, and S122-S123 into Eqs. S92-S93 yields

$$\mathrm{Da}^{*}\frac{\partial^2\left[{}^{32}\Sigma(\zeta)\right]}{\partial\zeta^2} - \frac{\partial\left[{}^{32}\Sigma(\zeta)\right]}{\partial\zeta} + \frac{\partial\Gamma(\zeta)}{\partial\zeta}\left(\frac{{}^{32}\Sigma(\zeta)}{{}^{34}\alpha_{\mathrm{S^{-II}/SO_4^{2-}}} \times {}^{34}\Sigma(\zeta) + {}^{32}\Sigma(\zeta)}\right)\left(\frac{\Sigma(\zeta)}{\kappa_\Sigma + \Sigma(\zeta)}\right) = 0 \tag{S144}$$

and

$$\mathrm{Da}^{*}\frac{\partial^2\left[{}^{34}\Sigma(\zeta)\right]}{\partial\zeta^2} - \frac{\partial\left[{}^{34}\Sigma(\zeta)\right]}{\partial\zeta} + \frac{\partial\Gamma(\zeta)}{\partial\zeta}\left(\frac{{}^{34}\alpha_{\mathrm{S^{-II}/SO_4^{2-}}} \times {}^{34}\Sigma(\zeta)}{{}^{34}\alpha_{\mathrm{S^{-II}/SO_4^{2-}}} \times {}^{34}\Sigma(\zeta) + {}^{32}\Sigma(\zeta)}\right)\left(\frac{\Sigma(\zeta)}{\kappa_\Sigma + \Sigma(\zeta)}\right) = 0, \tag{S145}$$

subject to the initial conditions

$${}^{32}\Sigma(0) = \frac{1}{1 + {}^{34}\mathrm{R}_{\Sigma_0}} \tag{S146}$$

and

$${}^{34}\Sigma(0) = \frac{{}^{34}\mathrm{R}_{\Sigma_0}}{1 + {}^{34}\mathrm{R}_{\Sigma_0}}, \tag{S147}$$

where ${}^{34}R_{\Sigma_0}$ is the sulphur-isotope ratio of seawater sulphate, and the boundary conditions

$$\lim_{\zeta\to\infty}\frac{\partial\left[{}^{32}\Sigma(\zeta)\right]}{\partial\zeta} = 0 \tag{S148}$$

and

$$\lim_{\zeta\to\infty}\frac{\partial\left[{}^{34}\Sigma(\zeta)\right]}{\partial\zeta} = 0. \tag{S149}$$

Equations S141, S144, and S145 define our isotopologue-specific non-dimensional sulphate model. Finally, like for our dimensional model, we convert resulting ${}^{32}\Sigma(\zeta)$ and ${}^{34}\Sigma(\zeta)$ values to delta notation following

$$\delta^{34}\mathrm{S}_{\Sigma(\zeta)} = \frac{{}^{34}\Sigma(\zeta)}{{}^{32}\Sigma(\zeta)}\left(\frac{1}{{}^{34}\mathrm{R}_{\mathrm{VCDT}}}\right) - 1. \tag{S150}$$

Sulphide isotopic composition

Substituting Eqs. S114-S115, S117, S119, S122-S123, S127, and S132-S134 into Eqs. S102-S103 yields

$$\Delta\mathrm{Da}^{*}\frac{\partial^2\left[{}^{32}\eta(\zeta)\right]}{\partial\zeta^2}-\frac{\partial\left[{}^{32}\eta(\zeta)\right]}{\partial\zeta}-\frac{\partial\Gamma(\zeta)}{\partial\zeta}\left(\frac{{}^{32}\Sigma(\zeta)}{{}^{34}\alpha_{\mathrm{S^{-II}/SO_4^{2-}}}\times{}^{34}\Sigma(\zeta)+{}^{32}\Sigma(\zeta)}\right)\left(\frac{\Sigma(\zeta)}{\kappa_\Sigma+\Sigma(\zeta)}\right)+\frac{\partial\Psi(\zeta)}{\partial\zeta}\left(\frac{{}^{32}\eta(\zeta)}{{}^{32}\eta(\zeta)+{}^{34}\eta(\zeta)}\right)\left(\frac{\eta(\zeta)}{\kappa_\eta+\eta(\zeta)}\right)=0 \tag{S151}$$

and

$$\Delta\mathrm{Da}^{*}\frac{\partial^2\left[{}^{34}\eta(\zeta)\right]}{\partial\zeta^2}-\frac{\partial\left[{}^{34}\eta(\zeta)\right]}{\partial\zeta}-\frac{\partial\Gamma(\zeta)}{\partial\zeta}\left(\frac{{}^{34}\alpha_{\mathrm{S^{-II}/SO_4^{2-}}}\times{}^{34}\Sigma(\zeta)}{{}^{34}\alpha_{\mathrm{S^{-II}/SO_4^{2-}}}\times{}^{34}\Sigma(\zeta)+{}^{32}\Sigma(\zeta)}\right)\left(\frac{\Sigma(\zeta)}{\kappa_\Sigma+\Sigma(\zeta)}\right)+\frac{\partial\Psi(\zeta)}{\partial\zeta}\left(\frac{{}^{34}\eta(\zeta)}{{}^{32}\eta(\zeta)+{}^{34}\eta(\zeta)}\right)\left(\frac{\eta(\zeta)}{\kappa_\eta+\eta(\zeta)}\right)=0 \tag{S152}$$

subject to the initial conditions

$$ {}^{32}\eta(0)=\frac{1}{1+{}^{34}\mathrm{R}_{\eta_0}}\eta_0 \tag{S153}$$

and

$$ {}^{34}\eta(0)=\frac{{}^{34}\mathrm{R}_{\eta_0}}{1+{}^{34}\mathrm{R}_{\eta_0}}\eta_0, \tag{S154}$$

where we again utilise the sulphur-isotope ratio of seawater $\mathrm{S}^{-\mathrm{II}}$ when defining ${}^{34}\mathrm{R}_{\eta_0}$ (only applicable when $\eta_0>0$; see Supplementary Discussion Sec. 8), and the boundary conditions

$$\lim_{\zeta\to\infty}\frac{\partial\left[{}^{32}\eta(\zeta)\right]}{\partial\zeta}=0 \tag{S155}$$

and

$$\lim_{\zeta\to\infty}\frac{\partial\left[{}^{34}\eta(\zeta)\right]}{\partial\zeta}=0. \tag{S156}$$

Equations S141, S142, and S151-S152 define our isotopologue-specific non-dimensional sulphide model. Finally, like for our dimensional model, we convert resulting ${}^{32}\eta(\zeta)$ and ${}^{34}\eta(\zeta)$ values to delta notation following

$$\delta^{34}\mathrm{S}_{\eta(\zeta)}=\frac{{}^{34}\eta(\zeta)}{{}^{32}\eta(\zeta)}\left(\frac{1}{{}^{34}\mathrm{R}_{\mathrm{VCDT}}}\right)-1. \tag{S157}$$

Pyrite isotopic composition

Substituting Eqs. S119, S127, and S132-S134 into Eqs. S109-S110 yields

$$\frac{\partial\left[{}^{32}\Pi(\zeta)\right]}{\partial\zeta}=-\frac{\partial\Psi(\zeta)}{\partial\zeta}\left(\frac{{}^{32}\eta(\zeta)}{{}^{32}\eta(\zeta)+{}^{34}\eta(\zeta)}\right)\left(\frac{\eta(\zeta)}{\kappa_\eta+\eta(\zeta)}\right) \tag{S158}$$

and

$$\frac{\partial\left[{}^{34}\Pi(\zeta)\right]}{\partial\zeta}=-\frac{\partial\Psi(\zeta)}{\partial\zeta}\left(\frac{{}^{34}\eta(\zeta)}{{}^{32}\eta(\zeta)+{}^{34}\eta(\zeta)}\right)\left(\frac{\eta(\zeta)}{\kappa_\eta+\eta(\zeta)}\right), \tag{S159}$$

subject to the initial conditions

$$^{32}\Pi(0) = \frac{1}{1 + {}^{34}\mathrm{R}_{\Pi_0}}\Pi_0 \tag{S160}$$

and

$$^{34}\Pi(0) = \frac{{}^{34}\mathrm{R}_{\Pi_0}}{1 + {}^{34}\mathrm{R}_{\Pi_0}}\Pi_0, \tag{S161}$$

where we again utilise the sulphur-isotope ratio of pyrite deposited on the sediment-water interface when defining ${}^{34}\mathrm{R}_{\Pi_0}$ (only applicable when $\Pi_0 > 0$; see Supplementary Discussion Sec. 8). Equations S143 and S158-S159 define our isotopologue-specific non-dimensional pyrite model. Finally, like for all previous species, we convert resulting ${}^{32}\Pi(\zeta)$ and ${}^{34}\Pi(\zeta)$ values to delta notation following

$$\delta^{34}\mathrm{S}_{\Pi(\zeta)} = \frac{{}^{34}\Pi(\zeta)}{{}^{32}\Pi(\zeta)}\left(\frac{1}{{}^{34}\mathrm{R}_{\mathrm{VCDT}}}\right) - 1, \tag{S162}$$

which describes the final output of our non-dimensional model.

4 Dimensional boundary conditions

Boundary-condition estimates are required to (i) validate our model against a globally distributed set of measured profiles, and (ii) extrapolate these results to estimate global pyrite burial fluxes and isotope compositions. For model validation, we use measured boundary-condition values at each sampling site when available. For missing measurements, we first attempt to estimate these using measured literature values from nearby sites (see Supplementary Data for all inputted measured data). If no nearby measurements exist, we extract values at sampling locations from globally gridded data products. To predict global pyrite flux and isotope predictions, we then utilise globally gridded products for each boundary condition of interest—either taken from the literature or derived as part of this study. Below, we describe each global boundary value product—including their derivations where relevant—separated into independent variables (i.e., calculated directly) and dependent variables (i.e., derived from other gridded products). We additionally validate each globally gridded boundary value product by interpolating and comparing to available measured data at our validation sampling sites (see Extended Data Table 3 for summary).

4.1 Independent variables

Water depth, z_{sw}

We obtained a global bathymetric map with 5 arc-minute resolution from the NOAA National Centers for Environmental Information[49]. To assess offsets between global predictions and measured water depths, z_{sw}, at our validation sites, we linearly interpolated gridded pixel midpoints to the GPS coordinates of each coring location (Supplementary Data). This yields a measured vs. predicted GM regression slope of $b_1 = 1.01$, intercept of $b_0 = -6.64$, RMSE of 139 m and absolute mean deviation (MD) from the 1:1 line of 82 m (Supplementary Fig. 4). We interpret this as representing the uncertainty of our globally gridded water depth values.

Bottom-water temperature, T_{sw}

We similarly obtained global seafloor temperatures with 5 arc-minute resolution from the NOAA National Centers for Environmental Information[96]. Data are given at discrete depths; combined with globally gridded bathymetry described above, we extrapolated these vertically to predict T_{sw}, the temperature at the sediment-water interface. Like for water depth, we assessed the offsets between global predictions and measured bottom-water temperatures by interpolating gridded pixel midpoints to the GPS coordinates of each coring location (Supplementary Data). This yields a measured vs. predicted GM regression slope of $b_1 = 1.13$, intercept of $b_0 = 0.35$, RMSE of 2.29 °C and MD from the 1:1 line of 1.77 °C (Supplementary Fig. 5), which we interpret as representing the uncertainty of our globally gridded bottom-water temperature values. Such uncertainty may partially result from seasonal biases, particularly in shallow-water sites, since gridded global data represents an annual average whereas measured values represent the temperature at the time of sample collection.

Total sediment thickness, z_{tot}

Globally gridded predictions of total sediment thickness, z_{tot}, with 5 arc-minute resolution were taken from Ref.[97] without further modification. Although not used directly for our model derivation, z_{tot} provides a maximum possible depth of pyrite formation (i.e., sulphate reduction cannot extend beyond the sediment column). We therefore utilise z_{tot} to truncate the maximum sulphidic anoxic zone depth at all points in the global ocean.

Sediment porosity, ϕ

Global surface sediment porosity was estimated by Ref.[26] using a random forest machine learning, based on six geological predictor grids derived from 2028 sample points (Nash-Sutcliffe Efficiency of 0.51). As above, we assessed the performance of this 5-arc-minute gridded product by interpolating to the GPS coordinates of each coring location (Supplementary Data). Data from Ref.[26] uses averaged porosity values in the top metre of sediment; we compare these values to average porosity measurements at validation sites, assuming that compaction in sedimentation processes can be largely ignored (Assumption 2). This yields a measured vs. predicted GM regression slope of $b_1 = 0.98$ and intercept of $b_0 = 0.01$, with an RMSE of 0.07 and MD from the 1:1 line of 0.06 (Supplementary Fig. 6a). We interpret this as representing the uncertainty of our globally gridded porosity values. The final input into our model is at 25 arc-minute resolution (Supplementary Fig. 6b). Importantly, resulting RMSE and MD values calculated here are similar to the possible bias due to compaction over our sediment depths of interest[56]; we assess the importance of this bias as part of our sensitivity test procedure (Supplementary Discussion Sec. 8).

Sedimentation rate, w

Globally gridded sedimentation rates have been estimated by several authors using various regression and machine learning techniques[28,36,50]. We first evaluate the performance of each by comparing to measured sedimentation rate values at each of our model validation sites (Supplementary Data) before choosing which dataset to adopt here.

First, Ref.[36] empirically estimated sedimentation rate using multiple linear regression on the measured dataset of Ref.[137]. Because Ref.[36] do not provide a globally gridded sedimentation rate output dataset, we recalculate their results using their governing equation:

$$\log_{10}(w) = -2.236 + 0.838\log_{10}(\text{Chla}) + 0.090\log_{10}(\text{Chla} + z_{\text{sw}}), \tag{S163}$$

where Chla is satellite-derived sea surface chlorophyll a concentration in units of $\text{mg}\,\text{m}^{-3}$, z_{sw} is water depth in m, and w is sedimentation rate in units of $\text{cm}\,\text{yr}^{-1}$ (for consistency, this was converted from units of $\text{cm}\,\text{kyr}^{-1}$ as reported in the original publication; their regression: $R^2 = 0.93$, $n = 26$). Here, we adopt chlorophyll a concentrations (1997 to 2010 average) from Ref.[138] and water depth as described above, both with 5 arc-minute resolution. Like before, we assessed the offsets between this prediction and measured sedimentation rates by interpolating from calculated gridded data to the GPS coordinates of each coring location (Supplementary Data). This yields a measured vs. predicted GM regression slope for log-transformed data of $b_1 = 0.73$ and intercept of $b_0 = 4.67$, with an RMSE of 0.49 and MD from the 1:1 line of 1.00 log units (Supplementary Fig. 7a).

Second, Ref.[50] similarly empirically estimated sedimentation rate using multiple linear regression on a globally distributed set of training data, yielding the governing equation:

$$\log_{10}(w) = -0.98193 - 0.27933\log_{10}(z_{\text{sw}}) - 0.60214\log_{10}(D) + 0.61702\log_{10}(\text{Chla}), \tag{S164}$$

where D is distance to the nearest coast in km and all other variables are as described above (their regression: $R^2 = 0.60$, $n = 1704$). Because Ref.[50] similarly do not provide a globally gridded sedimentation rate output dataset, we recalculate their results to generate a 5 arc-minute gridded dataset using Eq. S164, distances to the nearest coastline from Ref.[139], and water depth and chlorophyll a as described above. We again assessed offsets between this prediction and measured sedimentation rates by interpolating gridded data to the GPS coordinates of each coring location (Supplementary Data). This yields a measured vs. predicted GM regression slope for log-transformed data of $b_1 = 0.83$ and intercept of $b_0 = -1.54$, with an RMSE of 0.59 and MD from the 1:1 line of 1.32 log units (Supplementary Fig. 7b).

Finally, Ref.[28] utilised a machine learning k-nearest neighbours approach to estimate global sedimentation rates, with $n = 1744$ globally distributed measurements used as training data and a resulting measured vs. predicted regression coefficient of $R^2 = 0.879$. Unlike other predictions, Ref.[28] do provide

a globally gridded sedimentation rate output data product. Like before, we assessed the offsets between this prediction and measured sedimentation rates by interpolating gridded data to the GPS coordinates of each coring location (Supplementary Data). This yields a measured vs. predicted GM regression slope for log-transformed data of $b_1 = 0.86$ and intercept of $b_0 = -0.22$, with an RMSE of 0.66 and MD from the 1:1 line of 0.78 log units (Supplementary Fig. 7c).

Despite the slightly higher regression RMSE value, only the predictions from Ref.[28] provide an unbiased sedimentation rate prediction for our validation sites (i.e., closest to the 1:1 line, as evidenced by lowest MD and an intercept nearest to zero). Based on these results, we utilise the globally gridded predictions with 5 arc-minute resolution from Ref.[28] for all calculations performed here (Supplementary Fig. 7d). We interpret resulting RMSE and MD values calculated here using the predictions of Ref.[28] as representing the uncertainty of our globally gridded sedimentation rate dataset; we will assess the importance of this uncertainty as part of our sensitivity test procedure (Supplementary Discussion Sec. 8).

Organic carbon content at the sediment-water interface, G_{sw}

Like for sedimentation rates, globally gridded organic carbon contents at the sediment-water interface, G_{sw}, have been estimated by several authors using various regression and machine learning techniques[140–142]. Several additional studies provide global compilations of G_{sw} at discrete sampling points but without generating globally gridded data products[24,25]. We again evaluate the performance of each product by comparing to measured G_{sw} at each of our model validation sites (Supplementary Data) before choosing which dataset to adopt here.

First, Ref.[140] empirically estimated OC content (as dry bulk wt %) in the top 5 cm of sediment for 33 globally distributed marine provinces using a combined kriging and semi-variogram analysis approach with $n = 5500$ measurements as training-data input. Like for sedimentation rates, we assessed the offsets between this prediction and measured organic carbon content at our validation sites by interpolating gridded data to the GPS coordinates of each coring location (Supplementary Data). This yields a measured vs. predicted GM regression slope for log-transformed data of $b_1 = 0.71$ and intercept of $b_0 = -0.08$, with an RMSE of 0.43 and MD from the 1:1 line of 0.43 log units (Supplementary Fig. 8a).

Second, Ref.[141] utilised a machine learning k-nearest neighbours approach to estimate global OC content (as dry bulk wt %) in the top 5 cm, using the dataset of Ref.[140] as training data and resulting in measured vs. predicted regression coefficient of $R^2 = 0.78$ (as described in the original publication). We again assessed the offsets between this prediction and measured organic carbon content by interpolating data to the GPS coordinates of each coring location (Supplementary Data). This yields a measured vs. predicted GM regression slope for log-transformed data of $b_1 = -1.79$ and intercept of $b_0 = 0.68$, with an RMSE of 0.90 and MD from the 1:1 line of 1.05 log units (Supplementary Fig. 8b).

Third, Ref.[142] empirically estimated OC stocks in the top 1 m of sediment (as $\mathrm{g\,OC\,m^{-2}}$ seafloor area) using a random forest machine learning approach and twelve physical/environmental predictor variables. For $n = 11578$ globally distributed measurements used as training data; their results showed a measured vs. predicted regression coefficient of $R^2 = 0.76$. We converted their OC stock output dataset to OC content (in dry bulk wt %) using sediment porosity as described above and assuming an average dry-bulk density of $\rho_{\mathrm{sol}} = 2.7\ \mathrm{g\,cm^3_{sol}}$ (Ref.[98]). We then compared the offsets between predicted and measured organic carbon content at our validation sites by interpolating gridded data to the GPS coordinates of each coring location (Supplementary Data). This yields a measured vs. predicted GM regression slope for log-transformed data of $b_1 = 0.56$ and intercept of $b_0 = 0.11$, with an RMSE of 0.27 and MD from the 1:1 line of 0.30 log units (Supplementary Fig. 8c).

Finally, in an attempt to improve model predictions at our validation coring sites, we linearly interpolated discrete G_{sw} values provided (as dry bulk wt %) by Ref.[25] ($n = 6453$), Ref.[24] ($n = 15798$), and a combination of these two datasets ($n = 17315$; Supplementary Fig. 8d-f). The data from Ref.[25] and Ref.[24] alone provide reasonable fits to our sampling sites (Ref.[25] measured vs. predicted GM regression of log-transformed data: slope, $b_1 = 0.78$; intercept, $b_0 = 0.03$; RMSE = 0.37; MD = 0.32 log units; Ref.[24] measured vs. predicted GM regression of log-transformed data: slope, $b_1 = 0.70$; intercept, $b_0 = 0.01$; RMSE = 0.37; MD = 0.35 log units). Nevertheless, we use the larger combined dataset for all calculations here due to its smaller RMSE value and greater number of sampling points (measured vs. predicted GM regression of log-transformed data: slope, $b_1 = 0.74$; intercept, $b_0 = 0.00$; RMSE, 0.35; MD = 0.30 log units). We therefore utilise the combined data to predict a map of organic carbon at 25 arc-minute resolution, which we use as input to our global model (Supplementary Fig. 8g). We interpret resulting RMSE and MD values calculated here using this combined dataset as representing the uncertainty of our globally gridded G_{sw} dataset; we will assess the importance of this uncertainty as part of our sensitivity test procedure (Supplementary Discussion Sec. 8). To assess the extent of oxic OC respiration above the

onset of the sulphidic anoxic zone, we additionally calculate the difference between G_{sw} and G_0. Following Eq. S17, this is calculated as

$$\begin{aligned}\Delta G &= G_{\text{sw}} - G_0 \\ &= G_{\text{sw}}\left[1 - \left(\frac{1}{1 + \frac{k_{G_{\text{sw}}} z_{\text{MLD}}}{aw}}\right)^a\right],\end{aligned} \tag{S165}$$

where we implicitly assume here that the top of the sulphidic anoxic zone corresponds to z_{MLD} (Assumption 4). Equation S165 states that $\Delta G \geq 0$ always by an amount proportional to $k_{G_{\text{sw}}}$, z_{MLD}, and $1/w$. Thus, for a given $k_{G_{\text{sw}}}$ value, we find the limits: $\lim_{z_{\text{MLD}}\to 0} G_0 = G_{\text{sw}}$, $\lim_{w\to\infty} G_0 = G_{\text{sw}}$, $\lim_{z_{\text{MLD}}\to\infty} G_0 = 0$, and $\lim_{w\to 0} G_0 = 0$, as expected. Globally gridded ΔG values range from $\sim 10^{-4}$ in the abyssal open ocean to $\sim 10^{0}$ in some regions on the continental shelves (Supplementary Fig. 9).

Reactive iron content at the sediment-water interface, F_{sw}

Unlike for OC content, we are aware of no previous compilations for F_{sw}, the reactive iron content at the sediment-water interface. We therefore estimate this property in three steps: First, we determine reactive iron content, Fe_{HR}, as a function of total iron content, Fe_{tot}, for available data from Refs.[128,129,143,144]. Using GM regression, this yields the relationship

$$\text{Fe}_{\text{HR}} = (0.29 \pm 0.09)\text{Fe}_{\text{Tot}}, \tag{S166}$$

where uncertainty is reported as regression 95 % CI ($n = 9$; $R^2 = 0.86$; RMSE = 0.33; Supplementary Fig. 10a). Regression intercept is statistically equal to zero (-0.0045 ± 3.02; p-value > 0.05) and is therefore omitted here. This result is similar to other global estimates for the fraction of total iron that is present as reactive iron minerals[145]. Second, we generate a global map of discrete Fe_{tot} values at the sediment-water interface provided by Ref.[25] ($n = 1210$), and we convert these to Fe_{HR} content by multiplying by 0.29 following Eq. S166. We additionally complement this derived Fe_{HR} dataset with directly measured Fe_{HR} values from Refs.[99–102]. Third, we generate a globally gridded estimate of reactive iron content at the sediment-water interface with 25 arc-minute resolution (as dry bulk wt %) using cubic spline interpolation of these data (Supplementary Fig. 10c).
Like for all other variables, we calculated the offsets between predicted and measured Fe_{HR} content at our validation sites by interpolating gridded data to the GPS coordinates of each coring location (Supplementary Data). This yields a measured vs. predicted GM regression slope of $b_1 = 0.25$ and intercept of $b_0 = 0.71$, with an RMSE of 0.15 wt % and MD from the 1:1 line of 0.51 wt % (Supplementary Fig. 10b), which we interpret as representing the uncertainty of our globally gridded Fe_{HR} values. We will again assess the importance of this uncertainty as part of our sensitivity test procedure (Supplementary Discussion Sec. 8).

4.2 Dependent (derived) variables

Sulphate diffusivity, D_S

Following Refs.[27], we calculate sulphate diffusivity as a function of sediment porosity (itself related to sediment tortuosity) and temperature as

$$D_S(T) = \frac{D_{S,0}(T)}{1 + n(1-\phi)}, \tag{S167}$$

where $D_{S,0}(T)$ is the temperature-dependent sulphate diffusivity in seawater solution[103] and n is an empirical constant. Here we let $n = 2.5$, representing an equal mixture of clay-silt ($n = 2$) and sandy ($n = 3$) sediments[27]. Following Ref.[103], we calculate $D_{S,0}(T)$ as a linear function of T as

$$D_{S,0}(T) = 7.1 \times T + 156.5, \tag{S168}$$

where T is in °C and $D_{S,0}(T)$ is in units of $\text{cm}^2\text{yr}^{-1}$. Equation S168 was empirically determined over a temperature range of 0 to 30 °C (Ref.[103]) and is thus appropriate for the temperatures of interest here. Finally, we determined sulphate diffusivity globally at 25 arc-minute resolution using Eqs. S167-S168 and globally gridded estimates of ϕ and T_{sw} described above (Supplementary Fig. 11). For the environmental

conditions of interest here, calculated error in ϕ (Supplementary Fig. 6) and T_{sw} (Supplementary Fig. 5), as well as uncertainty in our choice of n, propagates to ca. ±15 % relative error in resulting D_S values. We interpret this as representing the uncertainty of our globally gridded values, and we again assess the importance of this as part of our sensitivity test procedure (Supplementary Discussion Sec. 8).

Sulphide diffusivity, D_H

Like for sulphate, we calculate sulphide diffusivity as a function of sediment porosity and temperature as

$$D_H(T) = \frac{D_{H,0}(T)}{1 + n(1-\phi)}, \tag{S169}$$

where $D_{H,0}(T)$ is the temperature-dependent sulphide diffusivity in seawater solution and we let $n = 2.5$, as above. Based on data in Refs.[95,104], we calculate the temperature dependence of $D_{H,0}(T)$ as

$$D_{H,0}(T) = 12.1 \times T + 600.0, \tag{S170}$$

where T is in °C and $D_{H,0}(T)$ is in units of $\mathrm{cm^2 yr^{-1}}$, as above. Like for sulphate, we determined sulphide diffusivity globally at 25 arc-minute resolution using Eqs. S169-S170 and globally gridded estimates of ϕ and T_{sw} described above (Supplementary Fig. 12). For the environmental conditions of interest here, calculated error in ϕ (Supplementary Fig. 6) and T_{sw} (Supplementary Fig. 5), as well as uncertainty in our choice of n, again propagates to ca. ±15 % relative error in resulting D_H values. We interpret this as representing the uncertainty of our globally gridded values, and we again assess the importance of this as part of our sensitivity test procedure (Supplementary Discussion Sec. 8).

Oxygen-penetration depth, z_{OPD}

Like for sedimentation rates and OC contents, several authors have generated global predictions of oxygen-penetration depth, z_{OPD}, by regressing measured values against primary productivity and/or water depth[105–107]. Despite reasonable predictions in shelf areas, these models often underestimate z_{OPD} in slope and abyssal zones, and they fail to account for the known complete oxygen penetration to basement in some regions of the deep ocean[51]. We therefore attempt to improve global predictions here by re-calculating oxygen penetration depth as a power-law function of sedimentation rate, w, and total sediment thickness, z_{tot}, as proposed by Ref.[51].

Using an updated measured z_{OPD} dataset of Ref.[105] with w and z_{tot} estimates as described above, this yields the following multiple-linear regression correlation coefficients: intercept, $b_0 = 0.79$ (SE = 0.20, p-value = 1.0×10^{-4}); w slope, $b_1 = -0.41$ (SE = 0.032, p-value = 1.7×10^{-26}); z_{tot} slope, $b_1 = -0.08$ (SE = 0.06, p-value > 0.05). However, because z_{tot} not a statistically significant predictor of z_{OPD}, we exclude this input variable and instead calculate z_{OPD} as a power-law function of w only. Using GM regression, this yields

$$\log_{10}(z_{\mathrm{OPD}}) = (-0.69 \pm 0.07)\log_{10}(w) + (0.90 \pm 0.08), \tag{S171}$$

where z_{OPD} is in cm, w is in $\mathrm{cm\,yr^{-1}}$, and uncertainty is reported as regression 95 % CI ($n = 222$; $R^2 = 0.45$; RMSE = 0.44; Supplementary Fig. 13a). We use Eq. S171 with globally gridded sedimentation rate estimates described above (Supplementary Fig. 7d) to calculate a global map of z_{OPD} at 25 arc-minute resolution (Supplementary Fig. 13b).

Mixed-layer depth, z_{MLD}

Because bioturbation often extends ~10-20× deeper than instantaneously measured z_{OPD} (e.g., Refs.[38,58,62]), we treat the onset of the sulphidic anoxic zone as equal to the mixed-layer depth, z_{MLD} (Assumption 4). Several authors have compiled globally distributed datasets of discrete z_{MLD} values[45,146]. However, Ref.[146] do not provide their compiled dataset; we therefore use the updated z_{MLD} dataset of Ref.[45] ($n = 1780$) to generate global estimates. Still, because data compiled by Ref.[45] are largely limited to shelf and slope areas in the temperate North Atlantic ($n = 1312$, or 74 % of the total dataset), we do not attempt to directly interpolate discrete z_{MLD} values to generate a globally gridded map. Rather, we explore the relationship between compiled z_{MLD} and z_{OPD} estimates since the latter is a strong function of sedimentation rate (Eq. S171), for which globally distributed data exist[28].

First, we compare the distributions of measured z_{OPD} from Ref.[105] and measured z_{MLD} from Ref.[45] (Supplementary Fig. 14a). We find a median z_{OPD} value of 0.48 cm (mean, 4.00 cm; inter-quartile range,

0.27-3.78 cm) and a median z_{MLD} value of 3.80 cm (mean, 5.68 cm; inter-quartile range, 1.90-7.30 cm). Assuming both distributions accurately capture the global range of values, this result supports previous estimates made at individual localities[38, 58,62] and suggests that the relationship

$$z_{\text{MLD}} \sim 10 \times z_{\text{OPD}} \tag{S172}$$

is globally valid. However, this approach may be biased, particularly if these distributions do not capture the same range of environmental conditions.

Therefore, to directly compare both depth measurements at individual sites, we interpolate our globally gridded z_{OPD} map to the GPS coordinates of each z_{MLD} sampling location in Ref.[45]. This yields a z_{OPD} vs. z_{MLD} GM regression for log-transformed data of $b_1 = 0.61$ and intercept of $b_0 = 0.55$, with an RMSE of 0.39 (Supplementary Fig. 14b). A calculated slope of $b_1 < 1$ and intercept of $b_0 > 0$ implies that $z_{\text{OPD}} << z_{\text{MLD}}$ in quickly sedimenting continental shelf and slope regions, as expected, but that z_{OPD} approaches and even exceeds z_{MLD} in slowly sedimenting abyssal regions where benthic faunal activity is limited yet oxygenated waters penetrate deeply due to diffusion or advective pumping.

Because pyrite formation predominantly occurs in continental shelves and slopes, here we generate a global map of z_{MLD} by multiplying our globally gridded z_{OPD} map calculated above by ten (Eq. S172), consistent with the measured differences in these two metrics within these regions (Supplementary Fig. 14). However, we test the importance of this choice by additionally solving our global model with $z_{\text{MLD}} = z_{\text{OPD}}$ as part of our sensitivity test procedure, as this condition better represents that of the abyssal open ocean (Supplementary Discussion Sec. 8).

Organic carbon reactivity at the sediment-water interface, $k_{G_{sw}}$

Predicting global OC reactivity, k_G, from environmental variables is challenging[147–149]. The reactivity of OC is extremely variable on a global scale, and it is influenced by factors such as deposition rate, bioturbation, temperature, organic carbon composition, availability of electron acceptors, microbial communities, and physical protection[147,150]. Furthermore, the conceptual approach chosen to define initial OC reactivity plays a significant role in its correlation to environmental parameters. Studies so far have attempted to predict OC reactivity using water depth[30, 151], bottom water oxygen concentration or total oxygen uptake (TOU)[149,152–154], sedimentation rates[137,155,156], or OC content and burial flux[152,153,156–158]. The consensus so far is that, independent of the model used, single environmental variables are insufficient to predict OC reactivity[148,149]. However, this may be attributed to the fact that such variables are often not independently available at the sites where reactivity is determined; rather, they must be interpolated from a predominantly coarse global grid, thus introducing uncertainties so significant as to obscure any underlying relationship.

To find strong predictors of OC reactivity, we calculate $k_{G_{\text{sw}}}$ using profiles from various environmental settings across the globe by fitting Eq. S3 to observed data. To be included here, profiles must contain 3 or more data points as well as an independently determined sedimentation rate value. To exclude data not in steady state, we further omit OC profiles displaying increasing OC with depth. This results in 138 OC profiles to fit (derived from the MOSAIC database; Ref.[24]). We then correlate resulting $k_{G_{\text{sw}}}$ values to several environmental predictor variables, including: (i) sedimentation rate, w; (ii) total OC content at the sediment-water interface[24], G_{sw}; (iii) radiocarbon (^{14}C) age; (iv) water depth, z_{sw}; (v) temperature, T_{sw}; (vi) porosity, ϕ; (vii) TOU[105]; and (viii) sediment grain size[159]. Variables (i)–(iii) were directly measured at each site, whereas variables (iv)–(viii) are interpolated from globally gridded maps to the GPS coordinates of these sites. Because several environmental predictors are likely correlated, we determine the variance influence factor (VIF) for each; that is, we assess how much the variance is inflated due to collinearity. All calculated VIF values are between 1-2, suggesting low to moderate collinearity[160]. We therefore evaluate each predictor individually to identify those with significant contributions to the response variable, $k_{G_{\text{sw}}}$ (Supplementary Fig. 15). We find sedimentation rate as the single best predictor for $k_{G_{\text{sw}}}$, yielding the relationship

$$\log_{10} k_{G_{\text{sw}}} = \log_{10}(0.05 \pm 0.04) + (1.51 \pm 0.19)w, \tag{S173}$$

where w is sedimentation rate in cm yr^{-1} (n = 138, RMSE = 0.82, R^2 = 0.46, p-value = 8.23×10^{-19}). In contrast, all other variables display less significant or statistically insignificant relationships with $k_{G_{\text{sw}}}$ ($R^2 \leq 0.2$; p-value $\geq 8 \times 10^{-8}$, or $R^2 \leq 0.2$; p-value ≥ 0.03 when including sedimentation rate as a second predictor; Supplementary Fig. 16).

We therefore use Eq. S173 with globally gridded sedimentation rate estimates described above (Supplementary Fig. 7d) to calculate a global map of $k_{G_{\mathrm{sw}}}$ at 25 arc-minute resolution (Supplementary Fig. 13). Finally, we interpret the uncertainty of this relationship as representing the uncertainty of our globally gridded values, and we again assess the importance of this as part of our sensitivity test procedure (Supplementary Discussion Sec. 8).
Like for G_{sw}, we additionally determine the difference in k_G calculated at the sediment-water interface and at the top of the sulphidic anoxic zone. Following Eq. S15, this is defined as

$$\begin{aligned} \Delta k_G &= k_{G_{\mathrm{sw}}} - k_{G_0} \\ &= \frac{k_{G_{\mathrm{sw}}}}{\frac{aw}{k_{G_{\mathrm{sw}}} z_{\mathrm{MLD}}} + 1} \end{aligned} \tag{S174}$$

where we again implicitly assume that the top of the sulphidic anoxic zone corresponds to z_{MLD} (Assumption 4). Equation S174 states that $\Delta k_G \geq 0$ always by an amount proportional to z_{MLD} and $1/w$. Thus, for a given sedimentation rate and $k_{G_{\mathrm{sw}}}$ value, we find the limits: $\lim_{z_{\mathrm{MLD}} \to 0} k_{G_0} = k_{G_{\mathrm{sw}}}$ and $\lim_{z_{\mathrm{MLD}} \to \infty} k_{G_0} = 0$, as expected. Globally gridded Δk_G values range from $\sim 10^{-4}$ in the abyssal open ocean, where $k_{G_{\mathrm{sw}}}$ is low, to $\sim 10^{-2}$ in some regions on the continental shelves (Supplementary Fig. 17).

Reactive iron reactivity at the sediment-water interface, $k_{F_{sw}}$

Similar to OC reactivity, we estimate $k_{F_{\mathrm{sw}}}$ at all available sites where reactive iron profiles exist (Supplementary Table 2). Specifically, we fit Eq. S49 to observed data, allowing $k_{F_{\mathrm{sw}}}$ to be a free parameter and using either directly measured or interpolated globally gridded sedimentation rate values as inputs. Because measured $\mathrm{Fe_{HR}}$ profiles—and thus resulting $k_{F_{\mathrm{sw}}}$ estimates—are sparse, we relate these to the more densely populated $k_{G_{\mathrm{sw}}}$ values rather than directly extrapolating globally. For sites where $k_{G_{\mathrm{sw}}}$ and $k_{F_{\mathrm{sw}}}$ are both available, we utilise GM regression with a zero intercept to derive the relationship:

$$\log_{10} k_{F_{\mathrm{sw}}} = (0.94 \pm 0.80) \log_{10} k_{G_{\mathrm{sw}}} \tag{S175}$$

($n = 26$, RMSE $= 1.47$, $R^2 = 0.26$, p-value $= 7.2 \times 10^{-3}$; Supplementary Fig. 18). Given that this result is statistically identical to the 1:1 line (i.e., slope statistically equal to unity), we assume a linear relationship between $k_{F_{\mathrm{sw}}}$ and $k_{G_{\mathrm{sw}}}$ with a ratio of initial OC and $\mathrm{Fe_{HR}}$ reactivities of $\chi = 1$. We therefore interpret globally gridded $k_{F_{\mathrm{sw}}}$ to be equal to our globally gridded map of $k_{G_{\mathrm{sw}}}$. Finally, we interpret the uncertainty of this relationship as representing the uncertainty of our globally gridded values, and we again assess the importance of this as part of our sensitivity test procedure (Supplementary Discussion Sec. 8).

5 Non-dimensional boundary conditions

Using the dimensional boundary conditions described above as inputs, we estimate globally gridded values for all non-dimensional boundary conditions that are required to solve our model. Below, we describe the predicted ranges of each in the modern ocean.

Damköhler number, Da*

We first calculate a globally gridded map of the modified Damköhler number, Da^*, at 25 arc-minute resolution following Eq. S115 and using our globally gridded $k_{G_{\mathrm{sw}}}$, D_S, and w estimates as inputs (Supplementary Figs. 7, 11, kg0global). This yields Da^* values ranging from ~ 1 in continental shelves to ~ 20 in the abyssal open ocean (Supplementary Fig. 19a). Because $k_{G_{\mathrm{sw}}} \propto w^{1.5}$ (Eq. S173) and D_S is relatively invariant compared to the orders-of-magnitude variability in w (Supplementary Figs. 7, 11), Eq. S115 reduces to $\mathrm{Da}^* \propto 1/w^{0.25}$. That is, sedimentation rate exerts a primary control such that Da^* is lower in rapidly sedimenting regions and higher in slowly sedimenting regions.
We additionally determine the difference in Da^* calculated at the sediment-water interface and at the top of the sulphidic anoxic zone, defined as

$$\Delta \mathrm{Da}^* = \mathrm{Da}^*_{\mathrm{sw}} - \mathrm{Da}^*_0, \tag{S176}$$

where we implicitly assume here that the top of the sulphidic anoxic zone corresponds to z_{MLD} (Assumption 4). Because D_S and w remain constant with depth (ignoring compaction; Assumption 2), Eq. S176

can be rewritten as

$$\Delta \text{Da}^* = \frac{\sqrt{D_S \Delta k_G}}{w}, \tag{S177}$$

For a given sedimentation rate, is it always the case that $\Delta k_G > 0$ by an amount proportional to z_{MLD} (i.e., rate coefficient decreases with depth; Eq. S174). It follows that $\Delta \text{Da}^* > 0$, also by an amount proportional to z_{MLD}. Globally, ΔDa^* ranges from << 1 in rapidly sedimenting continental shelves, where z_{MLD} is shallow, to ~ 5 in the abyssal open ocean, where z_{MLD} is deep (Supplementary Fig. 19b). As described in the main text, we utilise ΔDa^* to estimate the impact of oxygen penetration due to bioturbation on resulting pyrite contents and isotope compositions.

Organic carbon content, Γ_{sw}

Next, we calculate globally gridded maps of non-dimensional OC content, Γ_{sw}, at 25 arc-minute resolution following Eq. S117 and using our globally gridded G_{sw} estimate (Supplementary Fig. 8), as well as the porosity-dependent scaling factor f_G (Eq. S30) and the modern seawater sulphate concentration of S_0 = 28 mM, as inputs. This yields Γ_{sw} values ranging from ~ 0.03 in OC-poor regions of the open ocean to ~ 500 on continental shelves and highly productive open-ocean regions (e.g., Eastern Tropical South Pacific; Supplementary Fig. 20a).

Like for Da^*, we additionally determine the difference in Γ (Supplementary Fig. 20b) calculated at the sediment-water interface and at the top of the sulphidic anoxic zone, defined as

$$\Delta\Gamma = \Gamma_{\text{sw}} - \Gamma_0, \tag{S178}$$

where we implicitly assume here that the top of the sulphidic anoxic zone corresponds to z_{MLD} (Assumption 4). Because f_G remains constant with depth (ignoring compaction; Assumption 2) and S_0 is constant above the net sulphate reduction zone by definition, Eq. S178 can be rewritten as

$$\Delta\Gamma = \frac{f_G}{S_0}\Delta G, \tag{S179}$$

Similar to OC reactivity, for a given sedimentation rate, is it always the case that $\Delta G \geq 0$ always by an amount proportional to $k_{G_{\text{sw}}}$, z_{MLD}, and $1/w$ (i.e., OC content decreases with depth; Eq. S165). It follows that $\Delta\Gamma \geq 0$, also by an amount proportional to $k_{G_{\text{sw}}}$, z_{MLD}, and $1/w$. Globally, $\Delta\Gamma$ ranges from ~ 10^1 in rapidly sedimenting continental shelves, where z_{MLD} is shallow but organic carbon reactivity is high, to ~ 10^{-3} in the abyssal open ocean, where z_{MLD} is deep but organic carbon reactivity is low (Supplementary Fig. 20b). Like for ΔDa^*, we utilise $\Delta\Gamma$ to estimate the impact of oxygen penetration due to bioturbation on resulting pyrite contents and isotope compositions.

Reactive iron content, Ψ_{sw}

Finally, we calculate globally gridded maps of non-dimensional Fe_{HR} content, Ψ_{sw}, at 25 arc-minute resolution following Eq. S127 and using our globally gridded F_{sw} estimate (Supplementary Fig. 10), as well as the constant scaling factor f_F (Eq. S60) and the modern seawater sulphate concentration of S_0 = 28 mM, as inputs. This yields Ψ_{sw} values ranging from ~ 10^{-3} in Fe_{HR}-poor regions of the abyssal open ocean to ~ 10^2 in Fe_{HR}-rich continental shelves and slopes (Supplementary Fig. 21). Here we assume that Fe_{HR} does not undergo reductive dissolution above the onset of the sulphidic anoxic zone[38, 132] (Supplementary Discussion Sec. 2). Thus, unlike for Da^* and Γ, we explicitly let $\Psi_{\text{sw}} = \Psi_0$, the non-dimensional reactive iron content at z_{MLD}.

6 Solver verification

Our aim is to utilise MATLAB's robust ode45 and bvp5c solvers to solve the above-derived ordinary differential equations (ODEs). While the bvp5c solver is an efficient choice, it is sensitive to the chosen initial guess. We therefore use the ode45 solver to either solve the above equations directly or to provide an initial guess to use as input for the bvp5c solver. Although first-order ODEs (i.e., for solid-phase species) require only initial contents as inputs, second-order ODEs (i.e., for dissolved species) require both initial concentrations and their derivatives as inputs when using the ode45 solver. Whereas initial concentrations/contents for most species are either known or can be estimated (Supplementary Discussion Sec. 4), their derivatives are inherently unknown. To circumvent this issue, we first develop a "shooting

algorithm" approach to estimate initial derivatives. We then validate the robustness of our solver using the method of manufactured solutions[34]. We describe each step in detail below.

6.1 Shooting function

We first establish a shooting algorithm with a bisection function to estimate initial derivatives. In addition to known initial concentrations (Supplementary Discussion Secs. 4-5), we prescribe the derivatives at infinite depth for all second-order ODEs to be equal to zero (i.e., Eqs. S126, S137, S148, S155). We must therefore choose an initial derivative that satisfies the derivative at depth. To do so, we establish the following shooting algorithm:

1. Arbitrarily guess the initial derivatives for each governing equation (i.e., Eqs. S124, S135, S141, S142).
2. Numerically solve each governing equation over the prescribed depth range, chosen to extend significantly deeper than the expected depth of sulphate reduction and pyrite formation.
3. Determine the predicted derivative at the base of the prescribed depth range as an estimate of that at infinite depth.
4. Iterate steps (1) through (3), adjusting the chosen initial derivative using a bisection function until predicted and prescribed derivatives at depth agree within a pre-determined threshold value, λ (here chosen to be 10^{-6}).

Before applying this algorithm to our species of interest, we first test it using the method of manufactured solutions described below with a constructed analytical solution, termed $U_{\text{exact}}(\zeta)$. As shown in Supplementary Fig. 22a-b, our shooting function generates a numerical solution, $U_{\text{h}}(\zeta)$, that approaches $U_{\text{exact}}(\zeta)$ until $\partial U_{\text{exact}}(\zeta = \infty)/\partial\zeta - \partial U_{\text{h}}(\zeta = \infty)/\partial\zeta < \lambda$. The number of iterations required to satisfy λ varies based on the equation being solved; we set an iteration limit of 50, which is generally sufficient to achieve $\lambda < 10^{-6}$.
We further compare analytical and predicted results for the entire profile. To do so, we calculate the error between analytical and numerical solutions in two ways. First, we define

$$L_2 = \frac{1}{\sqrt{n}}||\mathbf{U}_{\text{exact}} - \mathbf{U}_{\text{h}}||_2, \tag{S180}$$

where $||\mathbf{x}||_2$ denotes the Euclidean norm of $\mathbf{x}$ such that

$$||\mathbf{x}||_2 = \sqrt{\sum_i x_i^2}, \tag{S181}$$

and n is the number of grid points. Second, we define

$$L_\infty = ||\mathbf{U}_{\text{exact}} - \mathbf{U}_{\text{h}}||_\infty, \tag{S182}$$

where $||\mathbf{x}||_\infty$ denotes the infinity norm of the absolute value of $\mathbf{x}$ such that

$$||\mathbf{x}||_\infty = \max_i |x_i|. \tag{S183}$$

As expected, both error metrics, L_2 and L_∞, decrease with decreasing shooting tolerance error, λ, for a range of chosen ode45 solver error tolerances (Supplementary Fig. 22c-d). For the shooting error value of $\lambda = 10^{-6}$ chosen here, we expect L_2 and L_∞ errors of $\sim 10^{-6}$, which we interpret as the numerical error characteristic of our shooting algorithm.

6.2 Manufactured solutions

Below, we first describe the theory of manufactured solutions, then we apply this to all non-linear differential equations of species of interest here—including concentrations and isotopic compositions.

Theory

The method of manufactured solutions[34] tests the accuracy of numerical solvers and implemented equations, determining the parameter space over which acceptable solutions can be found. We implement this method using the following steps:

1. First, we write the governing equation that we wish to validate as a differential operator, L, of any arbitrary function of depth, $U(\zeta)$. Here using the governing equation for non-dimensional sulphate (Eq. S124) as an example, this yields

$$L(U(\zeta)) = \mathrm{Da}^* \frac{\partial^2 U(\zeta)}{\partial \zeta^2} - \frac{\partial U(\zeta)}{\partial \zeta} + \frac{\partial \Gamma(\zeta)}{\partial \zeta} \left(\frac{U(\zeta)}{\kappa_\Sigma + U(\zeta)} \right). \tag{S184}$$

   Our goal is thus to assess the accuracy of the solutions to $L(U(\zeta)) = 0$ as determined by the numerical solver (e.g., where $U(\zeta)$ is sulphate concentration profile for the above example).

2. Because there is likely no analytical form to the solution that satisfies $L(U(\zeta)) = 0$ with which we can compare numerical results, we instead construct an arbitrary, non-trivial analytical solution, $U_{\mathrm{exact}}(\zeta)$ (the so-called "manufactured solution"). We avoid overly simplistic solutions (e.g., linear) to ensure non-zero higher-order derivatives. For the non-dimensional sulphate example, we define

$$U_{\mathrm{exact}}(\zeta) = [0.1 \sin(10\zeta) + 2]\, e^{-\zeta}. \tag{S185}$$

3. Third, we apply the operator L to our manufactured solution. $U_{\mathrm{exact}}(\zeta)$ need not satisfy $L(U(\zeta)) = 0$ (i.e., it is not the sulphate concentration profile predicted by our model), but rather yields a non-zero "source term", $Q(\zeta)$. We therefore define

$$L(U_{\mathrm{exact}}(\zeta)) = Q(\zeta). \tag{S186}$$

   Following the example in Eq. S184 above, this is written as

$$Q(\zeta) = \mathrm{Da}^* \frac{\partial^2 U_{\mathrm{exact}}(\zeta)}{\partial \zeta^2} - \frac{\partial U_{\mathrm{exact}}(\zeta)}{\partial \zeta} + \frac{\partial \Gamma(\zeta)}{\partial \zeta} \left(\frac{U_{\mathrm{exact}}(\zeta)}{\kappa_\Sigma + U_{\mathrm{exact}}(\zeta)} \right), \tag{S187}$$

   which we can solve analytically since it is known continuous function with finite derivatives.

4. Finally, we use the numerical solver to find the solution that satisfies

$$L(U(\zeta)) - Q(\zeta) = 0, \tag{S188}$$

   which yields a numerical approximation, $U_{\mathrm{h}}(\zeta)$, to the exact solution $U_{\mathrm{exact}}(\zeta)$.

We utilise this approach to test the stability field of the MATLAB ode45 and bvp5c solvers and the correct implementation of our derived governing equations, including when testing our shooting algorithm above. The agreement between the numerical approximation, $U_{\mathrm{h}}(\zeta)$, and the analytical solution, $U_{\mathrm{exact}}(\zeta)$, depends on three thresholds: (i) a user-defined error threshold for the shooting function, dictating convergence until the given boundary conditions are satisfied within this tolerance; (ii) an iteration limit for the shooting function; and (iii) relative and absolute error tolerances of the solver, which specify how accurately the numerical solution matches the solver's reference of the true solution. For all governing equations, we assess this agreement using L_2 as defined above (Eq. S180). We discuss each in detail below, and we assess solver stability for the given equations and parameter sets.

Sulphate concentration

We first assess the non-dimensional sulphate solution (Eq. S124). We choose $U_{\mathrm{exact}}(\zeta)$ that satisfies characteristics of the sulphate profile that are known *a priori*, specifically non-negative concentration values and negative derivatives. Following these constraints, we define $U_{\mathrm{exact}}(\zeta)$ to be the solution:

$$U_{\mathrm{exact}}(\zeta) = [0.1 \sin(10\zeta) + 2]\, e^{-\zeta}, \tag{S189}$$

which has the derivatives

$$\frac{\partial U_{\text{exact}}(\zeta)}{\partial \zeta} = [\cos(10\zeta) - 0.1\sin(10\zeta) - 2]\, e^{-\zeta}, \tag{S190}$$

and

$$\frac{\partial^2 U_{\text{exact}}(\zeta)}{\partial \zeta^2} = [-2\cos(10\zeta) - 9.9\sin(10\zeta) + 2]\, e^{-\zeta}. \tag{S191}$$

We then calculate the source term as

$$\begin{aligned} Q(\zeta) = \{&\text{Da}^* [-2\cos(10\zeta) - 9.9\sin(10\zeta) + 2] - [\cos(10\zeta) - 0.1\sin(10\zeta) - 2] \\ &+ \frac{\partial \Gamma(\zeta)}{\partial \zeta} \left(\frac{[0.1\sin(10\zeta) + 2]}{\kappa_\Sigma + [0.1\sin(10\zeta) + 2]\, e^{-\zeta}} \right) \Bigg\} e^{-\zeta}, \end{aligned} \tag{S192}$$

and we find the solution $U_{\text{h}}(\zeta)$ that satisfies $L(U(\zeta)) - Q(\zeta) = 0$. For this manufactured solution, we tested the stable parameter space for parameters Da^*, ζ, and Γ_0 (Supplementary Fig. 23). Resulting solver stability is largely determined by the chosen Γ_0 and maximum ζ. Thus, for extreme scenarios such as very small Da^* but very large Γ_0, the solution can become extremely stiff; this stiffness presents a challenge for most numerical solvers, leading to large L_2 values. Nevertheless, $L_2 < 1$ always within the parameter space considered here, suggesting that results can be confidently interpreted.

Sulphide concentration

Given that $\text{S}^{-\text{II}}$ is derived from sulphate reduction, its concentration profile will be described by a positive initial first derivative that does not exceed the absolute value of initial sulphate loss by MSR (i.e., the first derivative of the sulphate concentration profile). We therefore construct a manufactured solution, $U_{\text{exact}}(\zeta)$, which ensures non-negative concentrations and a positive initial derivative. This approach allows us to constrain the solution and initial guesses for the shooting function, analogous to the solution of non-dimensional $\text{S}^{-\text{II}}$ concentrations. Specifically, we define

$$U_{\text{exact}}(\zeta) = 1 - \cos(\zeta) e^{-\zeta}. \tag{S193}$$

This function has the derivatives

$$\frac{\partial U_{\text{exact}}(\zeta)}{\partial \zeta} = [\cos(\zeta) + \sin(\zeta)]\, e^{-\zeta} \tag{S194}$$

and

$$\frac{\partial^2 U_{\text{exact}}(\zeta)}{\partial \zeta^2} = -2\sin(\zeta) e^{-\zeta}. \tag{S195}$$

Analogous to before, we rewrite Eq. S135 as an operator L that we operate on $U_{\text{exact}}(\zeta)$ to determine the source term:

$$\begin{aligned} Q(\zeta) = \Bigg\{&(2\Delta\text{Da}^* + 1)\sin(\zeta) - \cos(\zeta) - \frac{\partial \Gamma(\zeta)}{\partial \zeta} \left(\frac{\Sigma(\zeta)}{\kappa_\Sigma + \Sigma(\zeta)} \right) \\ &+ \frac{\partial \Psi(\zeta)}{\partial \zeta} \left(\frac{\cos(\zeta) - e^{\zeta}}{\kappa_\eta - \cos(\zeta) e^{-\zeta} + 1} \right) \Bigg\} e^{-\zeta}. \end{aligned} \tag{S196}$$

We then find the solution $U_{\text{h}}(\zeta)$ that satisfies $L(U(\zeta)) - Q(\zeta) = 0$ and compare this to $U_{\text{exact}}(\zeta)$ to test solver stability as a function of Da^*, ζ, Γ_0, and Ψ_0 (Supplementary Fig. 24). Since $L_2 < 1$ across the entire parameter space, we can be confident in the accuracy of the non-dimensional sulphide concentration solutions throughout the tested parameter range.

Sulphate isotopes

Because isotopologue-specific ODEs are coupled (i.e., calculated isotope ratio depends on both ^{32}S and ^{32}S isotopologues), we construct coupled manufactured solutions to assess the error on calculated isotopic ratios. To reflect the difference in isotopic abundances, we specifically choose a manufactured solution for ^{34}S that is three orders of magnitude smaller than for ^{32}S. First, we define the manufactured solution

$$^{32}U_{\text{exact}}(\zeta) = 5e^{-\zeta/4}, \tag{S197}$$

where ^{32}U indicates that this applies to the ^{32}S isotopologue. We then determine the derivatives

$$\frac{\partial\left[^{32}U_{\text{exact}}(\zeta)\right]}{\partial\zeta} = -\frac{5}{4}e^{-\zeta/4} \tag{S198}$$

and

$$\frac{\partial^2\left[^{32}U_{\text{exact}}(\zeta)\right]}{\partial\zeta^2} = \frac{5}{16}e^{-\zeta/4}. \tag{S199}$$

Analogous to before, we rewrite Eq. S144 as an operator L that we operate on $^{32}U_{\text{exact}}(\zeta)$ to determine the source term:

$$^{32}Q(\zeta) = \left\{-\frac{1}{16}\text{Da}^* - \frac{1}{4} + \frac{\partial\Gamma(\zeta)}{\partial\zeta}\left(\frac{1}{5e^{-\zeta/4} + {}^{34}\alpha_{\text{S}^{-\text{II}}/\text{SO}_4^{2-}} \times {}^{34}U_{\text{exact}}(\zeta)}\right)\left(\frac{\Sigma(\zeta)}{\kappa_\Sigma + \Sigma(\zeta)}\right)\right\}5e^{-\zeta/4}, \tag{S200}$$

where $^{34}U_{\text{exact}}(\zeta)$ is the manufactured solution for the ^{34}S isotopologue. We define this to be

$$^{34}U_{\text{exact}}(\zeta) = \frac{1}{1000}e^{-\zeta/5}, \tag{S201}$$

which has the derivatives:

$$\frac{\partial\left[^{34}U_{\text{exact}}(\zeta)\right]}{\partial\zeta} = -\frac{1}{5000}e^{-\zeta/5}, \tag{S202}$$

and

$$\frac{\partial^2\left[^{34}U_{\text{exact}}(\zeta)\right]}{\partial\zeta^2} = \frac{1}{25000}e^{-\zeta/5}. \tag{S203}$$

We similarly rewrite Eq. S145 as an operator L that we operate on $^{34}U_{\text{exact}}(\zeta)$ to determine the source term:

$$\begin{aligned}^{34}Q(\zeta) = \Bigg\{-\frac{1}{25}\text{Da}^* - \frac{1}{5} + \frac{\partial\Gamma(\zeta)}{\partial\zeta}&\left(\frac{^{34}\alpha_{\text{S}^{-\text{II}}/\text{SO}_4^{2-}}}{^{32}U_{\text{exact}}(\zeta) + 0.001\,{}^{34}\alpha_{\text{S}^{-\text{II}}/\text{SO}_4^{2-}} \times e^{-\zeta/5}}\right)\\ &\left(\frac{\Sigma(\zeta)}{\kappa_\Sigma + \Sigma(\zeta)}\right)\Bigg\}\frac{1}{1000}e^{-\zeta/5}.\end{aligned} \tag{S204}$$

We then find the numerical solutions $^{32}U_{\text{h}}(\zeta)$ and $^{34}U_{\text{h}}(\zeta)$ that satisfy the coupled system

$$\begin{cases} L(^{32}U(\zeta)) - {}^{32}Q(\zeta) = 0 \\ L(^{34}U(\zeta)) - {}^{34}Q(\zeta) = 0. \end{cases} \tag{S205}$$

When solving Eq. S205, we use MATLAB's bvp5c solver with our initial guess of total sulphate concentration determined above as input. Finally, we calculate an isotopic ratio for our numerical solutions

as

$$\delta^{34}U_{\mathrm{h}} = \frac{\left(\frac{^{34}U_{\mathrm{h}}(\zeta)}{^{32}U_{\mathrm{h}}(\zeta)}\right)}{^{34}\mathrm{R}_{\mathrm{VCDT}}} - 1, \tag{S206}$$

which we can compare to the exact manufactured isotopic ratio,

$$\delta^{34}U_{\mathrm{exact}} = \frac{\left(\frac{^{34}U_{\mathrm{exact}}(\zeta)}{^{32}U_{\mathrm{exact}}(\zeta)}\right)}{^{34}\mathrm{R}_{\mathrm{VCDT}}} - 1. \tag{S207}$$

The resulting isotopic stability field in Da^* vs. ζ space for several choices of Γ_0 (Supplementary Fig. 25a) is different than for sulphate concentrations (Supplementary Fig. 23). Specifically, the isotopic composition solver is stable (i.e., $L_2 < 1$) at non-dimensional depth $\zeta < 10$ for all Da^*. However, the solver's efficiency significantly decreases at larger ζ values. We therefore define the ζ at which sulphate concentrations become smaller than 10×10^{-9} mM as the maximum depth at which we solve for isotopic compositions. This choice is justified because pyrite isotopic composition is effectively constant below this depth (see Supplementary Discussion Sec. 7, below). Additionally, large ζ values lead to large deviations from the exact solution (i.e., $L_2 > 1$), indicating reduced confidence in results. We therefore limit our solutions to a maximum depth of $\zeta = 10$, which is well beyond pyrite formation depth for natural environmental settings.

Sulphide isotopes

As there are no additional constraints to consider when solving for sulphide isotopic compositions, we apply the same manufactured solutions used for sulphate isotopic compositions above (i.e., Eqs.S197 and S201). Similar to before, we rewrite Eq. S151 as an operator L, which we operate on $^{32}U_{\mathrm{exact}}(\zeta)$ to determine the source term for the ^{32}S isotopologue:

$$\begin{aligned} ^{32}Q(\zeta) = \Bigg\{ -\frac{1}{16}\Delta\mathrm{Da}^* - \frac{1}{4} - \frac{\partial\Gamma(\zeta)}{\partial\zeta}\left(\frac{^{32}\Sigma(\zeta)}{^{32}\Sigma(\zeta) + {^{34}\alpha_{\mathrm{S^{-II}/SO_4^{2-}}}} \times {^{34}\Sigma(\zeta)}}\right)\left(\frac{\Sigma(\zeta)}{\kappa_\Sigma + \Sigma(\zeta)}\right) \\ + \frac{\partial\Psi(\zeta)}{\partial\zeta}\left(\frac{1}{5e^{-\zeta/4} + {^{34}U_{\mathrm{exact}}(\zeta)}}\right)\left(\frac{\eta(\zeta)}{\kappa_\eta + \eta(\zeta)}\right)\Bigg\} 5e^{-\zeta/4}. \end{aligned} \tag{S208}$$

Similarly, we rewrite Eq. S152 as an operator L, which we operate on $^{34}U_{\mathrm{exact}}(\zeta)$ to determine the source term for the ^{34}S isotopologue:

$$\begin{aligned} ^{34}Q(\zeta) = \Bigg\{ -\frac{1}{16}\Delta\mathrm{Da}^* - \frac{1}{4} - \frac{\partial\Gamma(\zeta)}{\partial\zeta}\left(\frac{{^{34}\alpha_{\mathrm{S^{-II}/SO_4^{2-}}}} \times {^{34}\Sigma(\zeta)}}{^{32}\Sigma(\zeta) + {^{34}\alpha_{\mathrm{S^{-II}/SO_4^{2-}}}} \times {^{34}\Sigma(\zeta)}}\right)\left(\frac{\Sigma(\zeta)}{\kappa_\Sigma + \Sigma(\zeta)}\right) \\ + \frac{\partial\Psi(\zeta)}{\partial\zeta}\left(\frac{1}{0.001e^{-\zeta/5} + ^{32}U_{\mathrm{exact}}(\zeta)}\right)\left(\frac{\eta(\zeta)}{\kappa_\eta + \eta(\zeta)}\right)\Bigg\} \frac{1}{1000}e^{-\zeta/5}. \end{aligned} \tag{S209}$$

Analogous to sulphate isotopic compositions, we then find the numerical solutions $^{32}U_{\mathrm{h}}(\zeta)$ and $^{34}U_{\mathrm{h}}(\zeta)$ that satisfy the coupled system in Eq. S205, and we calculate isotopic ratios for the numeric and exact manufactured solutions following Eqs. S206 and S207. Resulting sulphide isotopic composition solutions are stable for all parameter values in which sulphate isotopic composition sultions are also stable (i.e., $L_2 < 1$; Supplementary Fig. 25b), indicating that results can be interpreted with confidence.

7 Model validation and performance

7.1 Comparison to global profile compilation

To verify that our model accurately and precisely predicts pyrite contents and isotopic values across a range of depositional environments, we assess its performance by solving the governing concentration and isotopic composition equations using local boundary conditions for 216 globally distributed sedimentary records compiled from the literature (Supplementary Table 2; Supplementary Fig. 26). We then compare model results to measured values to assess the goodness of fit. This involves the following steps:

1. Extract independent dimensional boundary condition values at each coring site (z_{sw}, T_{sw}, ϕ, w, G_{sw}, F_{sw}), either directly using reported data where available (Supplementary Data) or indirectly by interpolating from our globally gridded datasets to the GPS points of interest (Supplementary Discussion Sec. 4).

2. Calculate dependent dimensional boundary condition values at each coring site (D_S, D_H, z_{MLD}, $k_{G_{sw}}$, $k_{F_{sw}}$; Eqs. S167-S173), again either directly using reported independent boundary condition values where available (Supplementary Data) or indirectly by interpolating from our globally gridded datasets to the GPS points of interest (Supplementary Discussion Sec. 4).

3. Non-dimensionalise boundary conditions to derive $\mathrm{Da}^{\star}$, Γ_0, and Ψ_0 values (Eqs. S115, S117, S127) at each coring site.

4. Solve the non-dimensional model to calculate profiles of species concentrations [$\Gamma(\zeta)$, $\Sigma(\zeta)$, $\Psi(\zeta)$, $\eta(\zeta)$, $\Pi(\zeta)$; Eqs. S118-S121, S124, S129-S131, S135, S139] and isotopic compositions [$\delta^{34}\mathrm{S}_{\Sigma(\zeta)}$, $\delta^{34}\mathrm{S}_{\eta(\zeta)}$, $\delta^{34}\mathrm{S}_{\Pi(\zeta)}$; Eqs. S150, S157, S162] across the sulphidic anoxic zone at each coring site. Maximum depth of model solutions at each site is chosen to be $2\times$ the maximum non-dimensional depth of measured data or $\zeta = 10$, whichever is smaller.

5. Re-dimensionalise depth (Eq. S114), OC and $\mathrm{Fe_{HR}}$ contents (Eqs. S117, S127), and sulphur-species concentration (Eqs. S122, S132, S138) predictions.

6. Compare measured (Supplementary Data) vs. re-dimensionalised predicted values for all variables of interest at all depths for which measured data exist at each site. Specifically, calculate measured vs. predicted root-mean square error (RMSE) as

$$\mathrm{RMSE}_i^x = \frac{1}{\sqrt{n}}||\mathbf{x}_i - \hat{\mathbf{x}}_i||_2, \tag{S210}$$

   where $\mathbf{x}_i$ and $\hat{\mathbf{x}}_i$ are the vectors of measured and predicted values of variable x [$= G(z)$, $S(z)$, $F(z)$, $H(z)$, $P(z)$, $\delta^{34}\mathrm{S}_{S(z)}$, $\delta^{34}\mathrm{S}_{H(z)}$, $\delta^{34}\mathrm{S}_{P(z)}$], respectively, and n is the number of measurements of variable x at coring location i. Each entry in $\mathbf{x}_i$ and $\hat{\mathbf{x}}_i$ correspond to values at the depths for which measured data exist. Additionally, calculate measured vs. predicted residuals ($= x_{i,j} - \hat{x}_{i,j}$ for depth j at coring location i) in order to assess model/data misfit data structure and distributions.

Measured species contents and concentrations are generally well-described by our model. For example, OC content exhibits a global median RMSE of 0.68 wt % (n = 63 profiles; Extended Data Fig. 5a). This result is largely independent of sedimentary environment, with no statistically significant difference in RMSE between continental shelf ($0 < z_{sw} \leq 200$ m), slope ($200 < z_{sw} \leq 2000$ m), and abyssal-ocean ($z_{sw} \geq 2000$ m) regions, nor between sites displaying shallow ($0 < z_{\mathrm{SMT}} \leq 1$ m), intermediate ($1 < z_{\mathrm{SMT}} \leq 10$ m), or deep ($z_{\mathrm{SMT}} \geq 10$ m) SMT depths. One possible exception is the Black Sea, which displays a single profile with the highest observed RMSE, likely due to anoxic bottom-water conditions. However, due to the limited data available, drawing statistically significant conclusions for model OC content performance in anoxic setting is challenging. Furthermore, measured vs. predicted residuals approach a normal distribution and display a median close to zero for all data (n = 1374 total measurements) and when separated by water and SMT depth (Extended Data Fig. 5b-c), indicating no model bias when predicting OC content.

Sulphate concentration is similarly well-described, with a global median RMSE of 6.97 mM (n = 125 profiles), again showing no statistically significant difference between profiles described by different water or SMT depths (Extended Data Fig. 5d). This result is comparable to the average RMSE of 4 mM determined by Ref.[36] for their model. However, importantly, Ref.[36] fit observed sulphate concentration profiles using an exponential decay function with a decay coefficient as a free fitting parameter. The observation that our median RMSE is within a factor of two to the average of Ref.[36] therefore suggests that our boundary-value based model with no free parameters does not introduce significant uncertainty beyond that of sampling noise. Furthermore, measured vs. predicted residuals again approach a normal distribution with a near-zero median value for all data (n = 1935 total measurements) and when separated by water and SMT depth (Extended Data Fig. 5e-f). One exception is sites with $z_{\mathrm{SMT}} \geq 10$ m, which display a slight positive bias. However, such sites are not globally significant in terms of pyrite formation rate (Supplementary Discussion Sec. 8).

Unlike all other variables, $\mathrm{Fe_{HR}}$ content data are sparse, making globally robust measured vs. predicted comparisons challenging. Nevertheless, our model displays a global median RMSE of 0.33 wt % (n = 25 profiles; Extended Data Fig. 6a), although nearly half of all profiles originate from the Black Sea.

Like for other variables, RMSE is not a strong function of water or SMT depth, and measured vs. predicted residuals approach a normal distribution and display a median close to zero for all environments (n = 496 total measurements). However, limited data prevent a robust analysis of residual distributions, particularly for sites displaying shallow SMT depth (Extended Data Fig. 6b-c).
Similar to other species, sulphide concentrations are well-predicted by our model, with a global median RMSE of 1.66 mM (n = 42 profiles) and no difference in RMSE range for profiles described by different water or SMT depths (Supplementary Fig. 6d). Additionally, measured vs. predicted residuals for the entire dataset approach a normal distribution and display a median value near zero for all data (n = 741 total measurements) and when separated by water and SMT depth (Extended Data Fig. 6e-f), again indicating no model bias when predicting sulphide concentrations.
Finally, like other solid-phase species, our model predicts pyrite content with a global median RMSE of 0.32 wt % (n = 74 profiles) and no statistically significant differences in RMSE range between profiles described by different water or SMT depths (Fig. 1a). Measured vs. predicted residuals similarly approach a normal distribution and display a median value close to zero for most water- and SMT-depth environments (n = 897 total measurements). Possible exceptions are slope environments, in which residuals are nearly uniformly distributed, and sites with $z_{SMT} \geq 10$ m, which display a slight negative bias, opposite of the bias predicted for sulphate concentrations described above (Fig. 1b-c).
Like for species concentrations, sulphur-bearing species isotopic compositions are generally unbiased and well-predicted by our model. Specifically, sulphate δ^{34}S displays a median RMSE of 9.8 ‰ (n = 77 profiles) with measured vs. predicted residuals that approach a normal distribution and display a median value near zero for most water- and SMT-depth environments (n = 782 total measurements; Extended Data Fig. 7a-c). However, ≈20 % of profiles display RMSE values ≥100 ‰. This is due to the fact that our model continues to simulate isotopic compositions until sulphate reaches concentrations ≪1 mM; elevated RMSE in these profiles is thus driven by large measured vs. predicted offsets at depth. This can result from: (i) slight inaccuracies in prescribed fractionation factor [31], which propagate into large offsets due to Rayleigh fractionation when sulphate is nearly quantitatively consumed; (ii) changes in fractionation factor due to low extracellular sulphate and/or sulphide concentrations [19]; and/or (iii) contamination by sulphate from overlying, higher concentration porewaters. In particular, Ref. [53] showed that "traditional" porewater sampling techniques can lead to an over-prediction of sulphate concentrations in sulphate-poor porewaters by up to ≈400 µM, or ≈ 40× the true value. Because sulphate inherently becomes ^{34}S-enriched with depth (i.e., further distilled by Rayleigh fractionation), such contamination by overlying porewaters will drive observed δ^{34}S to lower values. This manifests in our compilation as a slight positive bias in residuals, particularly for abyssal open-ocean profiles (Extended Data Fig. 7b).
Nevertheless, because sulphate δ^{34}S offsets occur at depth, where sulphate reduction and pyrite production are (nearly) negligible, this does not propagate into large residuals for sulphide or pyrite δ^{34}S values. In particular, sulphide δ^{34}S displays a median RMSE of 14.9 ‰ (Extended Data Fig. 7d). Although this value is higher than for sulphate, such elevated median RMSE value likely results from a lack of data, with only n = 20 profiles reporting sulphide isotopic compositions. Still, measured vs. predicted residuals approach a normal distribution and display a median value near zero all most water- and SMT-depth environments (n = 371 total measurements; Extended Data Fig. 7e-f), suggesting no bias in our model results.
Finally, pyrite δ^{34}S profiles display a median RMSE value of 16.6 ‰ (n = 82 profiles; Fig. 1d), slightly higher than sulphate and sulphide. Pyrite isotope RMSE may be driven at least in part by deviations from our steady-state assumption (Assumption 3). That is, whereas dissolved sulphur-bearing species can respond to transient perturbations in environmental conditions via diffusion [35], pyrite cannot. Non-steady-state conditions on timescales shorter than that of total sediment column deposition may lead to short-term variability in pyrite δ^{34}S values and thus increase RMSE. Nevertheless, measured vs. predicted residuals approach a normal distribution and display a near-zero median value for most water- and SMT-depth environments (n = 1072 total measurements), although there exists a slight positive bias for shelf and abyssal open-ocean regions (Fig. 1e-f). Still, model results display the lowest RMSE and residual values in continental shelfs and where $z_{SMT} < 1$ m, where pyrite formation rates are expected to be highest globally.

7.2 Measured vs. interpolated boundary conditions

Because we calculate global pyrite burial and isotopic composition maps, it is critical to constrain how boundary-condition error propagates into uncertainty for these sulphur-cycle metric predictions. To do so, we compare model performance for all 216 validation sites when using locally measured boundary

condition values, as described above, to that when only using boundary condition values extracted from our globally gridded maps (Supplementary Discussion Sec. 4). When using median RMSE as a performance metric, both model conditions performed nearly equally well for concentrations and isotopic compositions of most species of interest (Supplementary Fig. 27). Importantly, this is particularly true for OC, sulphate, and reactive iron concentrations/contents, as well as for sulphate and pyrite isotopic compositions. In contrast, extracted globally gridded boundary conditions lead to slightly elevated RMSE for pyrite content and significantly elevated RMSE for sulphide isotopic compositions, whereas sulphide concentrations display lower RMSE.

Higher RMSE for pyrite content likely results from interpolation error when generating global maps of OC and Fe_{HR} content at the sediment-water interface; this is particularly true for Fe_{HR} due to a lack of globally distributed measurements (Supplementary Figs. 8-10). This lack of boundary condition data at shallow water depths affects model accuracy: only six pyrite concentration validation profiles in shelf regions contain defined boundary conditions (others, e.g., fjords and coastal estuaries, are outside of our 25 arc-minute resolution global grid), compared to 17 profiles with locally defined conditions. Similarly, higher RMSE for sulphide isotopic compositions mainly propagates from error in OC estimates at the sediment-water interface. Again, fewer sites with defined boundary conditions are available in shelf areas relative to those with measured boundary conditions, thus contributing to higher RMSE values. Increasing the total number of well-predicted profiles with defined boundary conditions in this region would therefore mitigate increased RMSE, both for pyrite content and sulphide isotopic composition.

In contrast, the lower RMSE for sulphide concentrations results from a higher predicted than observed sedimentation rate for validation sites in the Gulf of Mexico[161], resulting in a shallower mixed layer depth and thus inclusion of shallower data points. Conversely, local boundary conditions at these sites predict lower sedimentation rates, excluding most profiles due to insufficient data below the mixed layer depth. As there are few sulphide concentration validation sites available globally, the additional data resulting from the inclusion of these Gulf of Mexico sites propagates to a measurable shift in global median RMSE for this metric. Therefore, the observed RMSE shifts for pyrite content and sulphide concentration likely result from data distribution; we predict that such changes would shrink with increasing data density, particularly in shelf regions.

8 Model interpretation and sensitivity

8.1 Parameter space heat maps

To calculate parameter-space heat maps, we solve our non-dimensional model over a pre-defined grid of boundary conditions. To highlight changes in the isotopic difference between sulphur in buried pyrite and in seawater sulphate, we report pyrite isotopic composition for all heat maps as

$$\Delta_{\text{pyrite}} = \delta^{34}\text{S}_{S_0} - \delta^{34}\text{S}_{\Pi(\zeta_{\max})}, \tag{S211}$$

where $\delta^{34}\text{S}_{S_0}$ is the isotopic composition of seawater sulphate. By reporting isotopic compositions in this way, heat maps are insensitive to the starting sulphate isotopic composition and can thus be used when reconstructing Earth-history records (see Materials and Methods). Below, we describe the major features of each parameter-space heat map considered here.

The effect of organic carbon content (Γ_0 vs. Da*)

We first assess how signals vary in Γ_0 vs. Da* space; that is, as a function of OC-to-sulphate ratio vs. reactivity/diffusion-to-sedimentation ratio. We specifically generate heat maps for two Ψ_0 conditions spanning the range of modern observations. For the high Ψ_0 case, we observe a region of high pyrite content ($\Pi \to 14$) under conditions of high Γ_0 and moderate Da* (~ 10; Fig. 3a). This result highlights a the balance required to drive elevated pyrite contents: sedimentation must be fast enough such that sulphide can accumulate rather than diffuse away, yet slow enough such that reduced sulphate can be resupplied by downward diffusion from the overlying water column. In contrast, for the low Ψ_0 case, Π remains low (≤ 2) regardless of Γ_0 and Da* value (Fig. 3a), indicating the importance of Fe_{HR} content in driving pyrite formation and burial.

For both Ψ_0 conditions, $\Delta_{\text{pyrite}} \to 0$‰ under conditions of high Γ_0 and low Da* (Fig. 3b,d). This represents the "closed system" end member, where high OC content drives sulphate reduction to completion and high sedimentation rate prevents upward diffusion of sulphide. In contrast, for both Ψ_0 conditions, $\Delta_{\text{pyrite}} \to$ 70‰ under conditions of low Γ_0 and high Da*. In this case, low OC content limits sulphate reduction extent and high diffusivity leads to strong communication with overlying seawater, thus driving "open

system" conditions and full expression of the microbial fractionation factor, $^{34}\alpha$. Unlike for pyrite content, the similarity between both Ψ_0 conditions highlights the insensitivity of pyrite isotopic composition to Fe_{HR} content.

The effect of reactive iron content (Ψ_0 vs. Da)*

Next we assess how signals vary as a function of Fe_{HR}-to-sulphate ratio, Ψ_0, vs. Da* space. We specifically generate heat maps for two Γ_0 conditions spanning the range of modern observations. For both Γ_0 scenarios, we observe a region of shallow-forming, high pyrite content when $\Psi_0 \gg 1$ (although absolute pyrite content is greater for the high-Γ_0 case; Supplementary Fig. 29a,b,d,e). In contrast, $\Pi \to 0$ under low Ψ_0 conditions regardless of the value of Da* and Γ_0. This finding supports the conclusion that Fe_{HR} content is a major driver of pyrite formation and burial flux. In contrast, Δ_{pyrite} is insensitive to Ψ_0 for all values of Da* when $\Psi_0 \leq 10$ (Supplementary Fig. 29c,f). This finding reinforces the interpretation that pyrite isotopic composition is largely independent of Fe_{HR} content under all modern marine conditions[17]. Only under conditions of low Γ_0 and extremely high Ψ_0 will Δ_{pyrite} increase due to efficient removal of sulphide from pore spaces, thus increasing formation of isotopically depleted pyrite near the top of the sulphidic anoxic zone.

The effect of iron reactivity (χ vs. Da)*

Similar to reactive iron concentration, we assess how signals vary in χ vs. Da* space. That is, we test the importance of Fe_{HR} reactivity vs. reactivity/diffusion-to-sedimentation ratio. We specifically generate heat maps for high-Γ_0, high-Ψ_0 and low-Γ_0, low-Ψ_0 conditions. In general, all parameters (i.e., pyrite formation depth, content, and Δ_{pyrite}) are largely insensitive to changing χ value (Supplementary Fig. 30). Still, we do observe an small decrease in formation depth coupled to a moderate increase in pyrite content and Δ_{pyrite} value for both Γ_0 and Ψ_0 conditions under low Da* values. This likely reflects the fact that, all else being equal, pyrite forms higher in the sediment column under conditions of high χ. Because this sulphide is less isotopically distilled and thus more depleted in ^{34}S relative to deeper in the sediment column, this leads to slightly higher Δ_{pyrite} values for high-χ conditions.

The effect of bottom-water euxinia (η_0 vs. Da)*

Finally, we consider the impact of bottom-water euxinia by assessing how signals vary as functions of bottom-water sulphide concentration, η_0, vs. Da*. Bottom-water euxinia implies both an absence of O_2 as well as the presence of S^{-II} in the water column, which will impact pyrite burial flux and isotopic composition in three ways: (i) First, anoxia will lead to the deposition of higher concentrations of more reactive OC (i.e., since less has been aerobically respired). Due to the competing effects of increasing OC content and reactivity on Γ_0 and Da*, changes to OC properties alone are not expected to significantly impact pyrite content or isotope composition (c.f., bioturbation, which moves in the opposite direction along the same trajectory; Fig. 3). (ii) Second, a lower oxidative sulphide sink above the sulphidic anoxic zone (i.e., at the sediment-water interface in the case of bottom-water anoxia) will decrease the upward diffusive gradient of S^{-II}, leading to so-called "sulphide pooling"[17]. By raising sulphide concentrations within porewaters, such pooling drives MSR closer to equilibrium[19] and leads to less isotopically distilled sulphide for a given sulphate reduction flux, thus increasing Δ_{pyrite} (i.e., more "open-system" conditions). (iii) Third, downward diffusion of (potentially isotopically unique) S^{-II} from the overlying water column can buffer any isotopic signal derived from MSR-produced sulphide in porewaters, thus leading to pyrite that reflects water-column—rather than *in situ* produced—sulphide isotopic compositions.

Although we do not explicitly consider the effect of bottom-water oxygen on sulphate and sulphide diffusive buffering, we can implicitly assess this by increasing S^{-II} and decreasing SO_4^{2-} concentrations at the sediment-water interface. To do so, we generate heat maps of η_0 vs. Da* for high-Γ_0, high-Ψ_0 and low-Γ_0, low-Ψ_0 conditions. Because $\eta_0 \neq 0$ in this test, we must assign an isotopic composition to water-column sulphide. We assume a value of −40 ‰ VCDT based on modern Black Sea water-column sulphide measurements[162]; the exact isotopic trends determined here therefore likely do not not apply to euxinic oceans in Earth's geologic past, which may have contained isotopically distinct water-column sulphide. Nevertheless, this approach is useful for assessing model performance for modern Black Sea profiles.

For both high- and low-Γ_0 and Ψ_0 conditions, we observe a shoaling of pyrite formation depth with increasing η_0, particularly at high Da* values (Supplementary Fig. 33a, d). This reflects the fact that, when sedimentation rate is low, downward sulphide diffusion will lead to increased pyrite formation near the sediment-water interface. This mechanism similarly drives an increase in pyrite content with increasing η_0, particularly at high Da* values (Supplementary Fig. 33b, e). However, Δ_{pyrite} is largely insensitive to

water-column sulphide concentration for both Γ_0 and Ψ_0 scenarios if $\eta_0 < 0.1$ (Supplementary Fig. 33c,f). At high Da* values, observed Δ_{pyrite} values result from the fact that final pyrite isotopic composition simply reflects that of downward diffusing sulphide (here, −40 ‰ VCDT). This buffers any variability due to in-situ formation by MSR and leads to high Δ_{pyrite} values independent of η_0. In contrast, at low Da* values, sedimentation is fast enough to out-pace downward diffusion, leading to (near-)quantitative sulphate consumption, pyrite that is dominated by isotopically distilled sulphide, and thus low Δ_{pyrite} values independent of η_0. Only when $\eta_0 > 0.1$ does downward diffusion begin to increase Δ_{pyrite} even for low Da* conditions.

For the modern Black Sea, bottom-water S^{-II} concentrations do not exceed 400 µM (Ref.[46]), which translates to a non-dimensional concentration of $\eta_0 = 0.02$. For this η_0 value and any Da* value observed in modern marine sediments, we expect no significant impact on pyrite content and isotopic composition relative to the oxic bottom-water case. Nevertheless, the presence of bottom-water sulphide in the Black Sea likely leads to a shoaling of the pyrite formation zone.

8.2 Sensitivity tests

To further validate the performance and validity of our model results—particularly important when comparing to independent biogeochemical flux estimates—we perform a series of sensitivity tests by independently altering each boundary condition estimate and re-calculating global pyrite formation rate and weighted-average isotopic composition. We describe each sensitivity test in detail below.

Porosity, ϕ

We first assess the degree to which our assumption of no compaction (Assumption 2)—as well as the general uncertainty in estimated ϕ values—impacts global estimates of pyrite burial and isotopic composition. To do so, we utilise the larger of two sources of uncertainty in ϕ: (i) that of our porosity validation regression (RMSE = 0.07, MD = 0.06; Supplementary Fig. 6), and (ii) the maximum estimated decrease due to compaction within the sulphidic anoxic zone[56]. The latter yields an average ϕ decrease of 1.1 %, 6.7 %, and 7.7 % (absolute) in shelf, slope and abyssal regions. As a 7.7 % absolute decrease represents the most conservative uncertainty estimate, we use this value to calculate the impact of porosity on global pyrite concentration and isotopic composition. Specifically, we re-solve our global model after reducing ϕ at all points by 7.7 %; this change is additionally propagated to dependent boundary conditions (i.e., D_S and D_H; Supplementary Discussion Sec. 4 and conversion factors f_G,f_F, and f_P; Supplementary Discussion Sec. 2). Decreasing porosity exhibits a minor impact on global predictions for both pyrite burial flux (absolute increase of 6.96×10^{11} mol S yr^{-1}, or ~10.0 % relative to "standard" conditions) and isotopic composition (absolute increase of 0.9 ‰ relative to "standard" conditions; Extended Data Fig. 8).

Sedimentation rate, w

Second, we assess the degree to which uncertainty in sedimentation rate impacts predicted global pyrite burial flux and isotopic composition. To do so, we utilise w uncertainty as determined by our global validation regression (RMSE = 0.66, MD = 0.78 log units; Supplementary Fig. 7) in two ways:

(i) First, to isolate the effect of sedimentation rate alone, we re-solve our global model after multiplying w at all points by a factor of 3 and 1/3 (i.e., the increase and decrease, respectively, predicted by our regression MD error) while holding all dependent boundary conditions constant; we denote these solutions as w^*. Although not realistic given the observed dependencies on w, this exercise serves to better understand model dynamics. Increasing w^* results in a substantial increase in global predictions of pyrite burial flux (absolute increase of 7.54×10^{12} mol S yr^{-1}, or ~108 % relative to "standard" conditions) and isotopic composition (absolute increase of 9.3 ‰ relative to "standard" conditions; Extended Data Fig. 8). Decreasing w^* similarly decreases predicted global pyrite burial flux (absolute decrease of 5.18×10^{12} mol S yr^{-1}, or ~74 % relative to "standard" conditions) and, particularly, isotopic composition (absolute decrease of 17.1 ‰ relative to "standard" conditions; Extended Data Fig. 8). The predicted shifts resulting from changing w^* represent the largest change in predicted isotopic composition for any sensitivity test performed here.

(i) Second, to realistically assess overall error, we re-solve our global model after multiplying w at all points by a factor of 3 and 1/3 as before, while additionally propagating this change to dependent boundary conditions (i.e., z_{OPD}, z_{MLD}, $k_{G_{\text{sw}}}$, and $k_{F_{\text{sw}}}$; Supplementary Discussion Sec. 4). Increasing w and its dependencies results in an even larger shift in predicted pyrite burial flux (absolute increase of 1.21×10^{13} mol S yr^{-1}, or ~172.8 % relative to "standard" conditions), although changes in pyrite isotopic composition are significantly muted relative to the w^* case (absolute increase of 3.0 ‰ relative to

"standard" conditions; Extended Data Fig. 8). Similarly, decreasing w and its dependencies leads to a significant decrease in pyrite burial flux (absolute decrease of 4.54×10^{12} mol S yr^{-1}, or ~65.1 % relative to "standard" conditions) and a moderate decrease in predicted isotopic composition (absolute decrease of 4.1 ‰ relative to "standard" conditions; Extended Data Fig. 8).

Excluding dependencies thus results in a similar (when decreasing) or slightly smaller (when increasing) sensitivity of pyrite burial flux to changes in sedimentation rates but a significantly larger sensitivity of pyrite isotopic composition. Conceptually, this results from the fact that excluding dependencies leads to a nearly vertical vector in Da* vs. Γ_0 parameter space whereas including dependencies leads to an angled trajectory in this space (i.e., since Γ_0 is also impacted due to a change in z_{MLD} and thus G_0; Supplementary Fig. 31). Because this angled trajectory is nearly parallel to a line of constant isotopic composition, resulting isotopic variability is dampened. In contrast, the nearly vertical trajectory for the w^* case crosses lines of constant isotopic composition, thus resulting in larger sensitivity.

Organic carbon content at the sediment-water interface, G_{sw}

Third, we estimate how uncertainty in OC content at the sediment-water interface, G_{sw}, propagates to uncertainty in pyrite burial flux and isotopic composition. To do so, we utilise G_{sw} uncertainty as determined by our global validation regression (RMSE = 0.35, MD = 0.29 log units; Supplementary Fig. 8); specifically, we re-solve our global model after multiplying G_{sw} at all points by a factor of 1.3 and 0.7 to represent the increase and decrease, respectively, predicted by our regression MD error. Increasing G_{sw} exhibits a low-to-moderate impact on global predictions for both pyrite burial flux (absolute increase of 7.95×10^{11} mol S yr^{-1}, or ~11.4 % relative to "standard" conditions) and isotopic composition (absolute increase of 3.0 ‰ relative to "standard" conditions; Extended Data Fig. 8). Although opposite in sign, decreasing G_{sw} exhibits a similarly low-to-moderate impact on globally averaged pyrite burial flux (absolute decrease of 9.19×10^{11} mol S yr^{-1}, or ~13 % relative to "standard" conditions) and isotopic composition (absolute decrease of 3.7 ‰ relative to "standard" conditions; Extended Data Fig. 8).

Reactive iron content at the sediment-water interface, F_{sw}

Fourth, we asses how the uncertainty in reactive iron at the sediment-water interface, F_{sw}, propagates to uncertainty in global pyrite burial flux and isotopic composition predictions. Specifically, we re-solve our model after multiplying F_{sw} at all points by a factor of 2 and 0.5 to represent the increase and decrease, respectively, predicted by our global regression uncertainty (RMSE = 0.15, MD = 0.51 log units; Supplementary Fig. 10). Results indicate that global pyrite burial flux estimates are sensitive to F_{sw}. In particular, both doubling (absolute increase of 2.55×10^{12} mol S yr^{-1}, or ~36.5 % relative to "standard" conditions) and halving (absolute decrease of 2.37×10^{12} mol S yr^{-1}, or ~34.0 % relative to "standard" conditions) F_{sw} exerts a moderately large impact on burial flux (Extended Data Fig. 8). In contrast, pyrite isotopic composition is less sensitive to changes in F_{sw} (doubling: absolute decrease of 3.2 ‰ relative to "standard" conditions; halving: absolute increase of 2.5 ‰ relative to "standard" conditions; Extended Data Fig. 8).

Sulphate and sulphide diffusivities, D_S and D_H

Fifth, we assess the impact of uncertainty in diffusivities. Uncertainty in ϕ, T_{sw}, and choice of n propagates to approximately ±15 % relative error for both D_S and D_H. We therefore re-solve our global model after increasing and decreasing D_S and D_H for each point by 15 % in unison, since uncertainty will impact both diffusivities in the same direction. Our model is largely insensitive to changes in diffusivity. Specifically, increasing D_S and D_H exhibits a minor impact on global predictions for both pyrite burial flux (absolute increase of 7.90×10^{10} mol S yr^{-1}, or ~1.1 % relative to "standard" conditions) and isotopic composition (absolute decrease of 0.8 ‰ relative to "standard" conditions; Extended Data Fig. 8). Decreasing D_S and D_H exhibits a similarly minor impact on burial flux (absolute decrease of 1.06×10^{11} mol S yr^{-1}, or ~1.5 % relative to "standard" conditions) and no resolvable change in isotopic composition (absolute decrease of 0.8 ‰ relative to "standard" conditions; Extended Data Fig. 8).

Onset of sulphidic anoxic zone (i.e., z_{OPD} vs. z_{MLD})

Sixth, we test the importance of our choice of sulphidic anoxic zone onset on resulting pyrite burial flux and isotopic composition estimates. To do so, we re-solve our global model using the oxygen penetration depth, rather than the mixed layer depth, as our starting condition (i.e., we implicitly set $z_{\mathrm{MLD}} = z_{\mathrm{OPD}}$). This choice is consistent with abyssal open-ocean settings, where oxygen penetration may meet or exceed the bioturbated mixed layer (Supplementary Fig. 14; Supplementary Discussion Sec. 4); this sensitivity

test therefore conservatively spans the entire possible range of sulphate reduction onset. Resulting pyrite burial flux and isotopic compositions are insensitive to our choice of z_{MLD}. Results indicate that decreasing z_{MLD} by a factor of 10 exhibits a negligible impact on both pyrite burial flux (absolute increase of 2.47×10^{11} mol S yr^{-1}, or ~4 % relative to "standard" conditions) and isotopic composition estimates (absolute decrease of 0.7 ‰ relative to "standard" conditions; Extended Data Fig. 8).

Reactive iron reactivity at the sediment-water interface, $k_{F_{sw}}$

Seventh, we asses how uncertainty in χ propagates to uncertainty in globally averaged pyrite burial flux and isotopic composition. To do so, we utilise the χ uncertainty as determined by our global validation regression (RMSE = 1.47 log units; Supplementary Fig. 18). Specifically, we re-solve our global model after multiplying χ at all points by 0.1 and 10 to represent an increase and decrease, respectively, in iron reactivity relative to carbon reactivity. Lowering χ has a relatively large impact on estimates of global pyrite burial flux (absolute decrease of 2.0×10^{12} mol S yr^{-1}, or ~28.8 % relative to "standard" conditions) and isotopic composition (absolute increase of 6.8 ‰ relative to "standard" conditions; Extended Data Fig. 8). Increasing χ, in contrast, exhibits a low impact on estimates of both global pyrite burial flux (absolute increase of 1.50×10^{11} mol S yr^{-1}, or ~2.2 % relative to "standard" conditions) and isotopic composition (absolute decrease of 2.9 ‰ relative to "standard" conditions; Extended Data Fig. 8).

Organic carbon reactivity at the sediment-water interface, $k_{G_{sw}}$

Finally, we asses how uncertainty in $k_{G_{\mathrm{sw}}}$ propagates to uncertainty in globally averaged pyrite burial flux and isotopic composition. To do so, we utilise the $k_{G_{\mathrm{sw}}}$ uncertainty as a function of w as determined by our global validation regression (RMSE = 0.87 log units; Supplementary Fig. 16). Specifically, we re-solve our global model using the maximum and minimum possible values of $k_{G_{\mathrm{sw}}}$ within uncertainty at all points. Lowering $k_{G_{\mathrm{sw}}}$ has a moderate impact on estimates of global pyrite burial flux (absolute decrease of 9.94×10^{11} mol S yr^{-1}, or ~14.3 % relative to "standard" conditions) and isotopic composition (absolute increase of 5.3 ‰ relative to "standard" conditions; Extended Data Fig. 8). Increasing $k_{G_{\mathrm{sw}}}$ similarly exhibits a low-to-moderate impact on estimates of both global pyrite burial flux (absolute increase of 1.10×10^{11} mol S yr^{-1}, or ~1.7 % relative to "standard" conditions) and isotopic composition (absolute decrease of 1.7 ‰ relative to "standard" conditions; Extended Data Fig. 8).

Supplementary Tables

Supplementary Table 1 |: Reactive iron content (wt % Fe_{HR}) of individual phases from sites used to calibrate our Fe_{HR} reduction model.

Site	HFO*	Lepidocrocite*	Goethite†	Haematite†	Magnetite	Reference
FOAM	0.13	0.13	0.10	0.10	0.05	Refs.[94, 163]
Sta 18	0.85	0.85	0.46	0.46	n.d.	Ref.[94]
Sta 19	0.94	0.94	0.54	0.54	n.d.	Ref.[94]
average ($\mu \pm 1\sigma$)	0.64 ± 0.44	0.64 ± 0.44	0.36 ± 0.23	0.36 ± 0.23	0.05	–

*Reported as ammonium oxalate-extractable iron; assumed equal contributions of HFO and lepidocrocite.
†Reported as citrate dithionite-extractable iron; assumed equal contributions of goethite and haematite.
n.d. = not detected.

Supplementary Table 2 |: Sites compiled from the literature used for model validation in this study. All site names refer to those described in the original publications. See Supplementary Data for all compiled data.

Reference	Site Name	Reference	Site Name	Reference	Site Name
Aharon and Fu [161]	GC-185-2646-	Böttcher et al. [164]	L201-1225	Michaud et al. [165]	M2020-AC
	GC-185-2647-1		L201-1226		M2020-J
	GC-185-2647-2		L201-1227		M2020-P
	GC-185-2647-3		L201-1228	Middelburg et al. [77]	K11
	GC-185-2647-4		L201-1229		K3
	GC-232-2635-1		L201-1231		K4
	GC-232-2635-2	Borowski et al. [166]	ODP994		K9
	GC-232-2635-3		ODP995	Mossmann et al. [167]	680
	GC-232-2639-2		PC11-8		686
	GC-232-2639-3	Brüchert and Pratt [168]	B1999-CB	Neretin et al. [169]	N2004-7
	GC-232-2639-4		B1999-WB	Peketi et al. [143]	MD161-13
	GC-232-2643-2	Brüchert et al. [170]	B2001-D	Pellerin et al. [53]	M1
	GC-232-2643-3		B2001-F		M24
Aller et al. [57]	GS-4221		B2001-G		M5
	KS00-16	Calvert et al. [171]	BS4-14GC	Raven et al. [73]	GC
	KS00-17	Chanton et al. [58]	C1987-Oct80		MC
	OST-2-Low	Crémière et al. [172]	C2017-C1	Riedinger and Brunner [173]	C0004
	OST-2-Rising		C2017-C2		C0006
	RMT-2	Dale et al. [174]	22680		C0007
	SJ96-22	Diaz et al. [175]	BCCF10–01		C0008
Antler et al. [176]	BA1		BCCF10–04	Riedinger et al. [177]	13824-1
	HU		BCCF10–09		13863-1
	ODP1052		BCCF10–15	Sweeney and Kaplan [178]	26
	ODP807	Donahue et al. [179]	ODP1002		27
	P130	Hardisty et al. [180]	FOAM-1		38G
	Y1	Henneke et al. [181]	ABC15		50G
	Y2		ABC45		7G
Böning et al. [182]	14MC	Hu et al. [183]	H2017-D-8		SanPedroMartir
	18MC		H2017-D-F	Turchyn et al. [184]	U1385
	29MC		H2017-D7	Wehrmann et al. [185]	AB
	33MC	Jørgensen et al. [186]	RSB4		AC
	45MC		RSB5		GK
Böttcher et al. [187]	964		RSB6		GN
	965		RSB7		HA
	966	Ku et al. [188]	CK		HB
	967		SK		IA
	968	Ku et al. [189]	PB1		J
	969		PB3		Q
	970		PB4A		T
	970B		PB5	Wehrmann et al. [190]	U1317
	970C		PB6	Wehrmann et al. [191]	KFIN
	970D		SB1		KFMID2
	971A		SB3		SBFIN
	971B		SB4		KFMID1
	971C		SB5		SBFMID
	971D	Lein [192]	AB2656		SBFOUT
	72		GB2618		VMFIN
	973		GB2622		VMFMID
	BAS3		GB2682		VMFOUT
	RP47		GR2601	Werne et al. [193]	1002
	RP63		SS2611	Wijsman et al. [144]	10
Böttcher et al. [194]	B974		SS2631		13
	975	Lein and Ivanov [195]	Vy20-3205		16
	976	Lin et al. [196]	HS373		19
	977	Lin et al. [197]	2A		2
	978		DHCL11-2016		22
	979		XHCL27A		24
Böttcher et al. [198]	B2000b-DT	Lin et al. [199]	HS148		6
Böttcher et al. [200]	ODP1119		HS217		9
	ODP1120	Lin et al. [201]	973-4-2016	Yücel et al. [202]	8-19
	ODP1121	Lin et al. [203]	DHCL11-2017		8-23
	ODP1122		HD109		8-30
	ODP1123	Lin et al. [204]	GMGS2-16		8-7
Böttcher et al. [205]	B2000-LD	Liu et al. [206]	973-4-2016	Zhu et al. [207]	DH5-1
	BIOTRANS1	Liu et al. [208]	BB01		DH7-1
	CAST1		BB02	Zindorf et al. [129]	U1417
	NAST1		BB05	Zopfi et al. [209]	4
	SAST1	Llobet-Brossa et al. [210]	LB2002-D		7
	WAST-T1	Masterson et al. [211]	M2018-AB		14
	WAST1	Mazumdar et al. [128]	SASU973		18
Böttcher et al. [212]	ODP1124	Meister et al. [213]	ODP1229		
	ODP1125				

Supplementary Figures

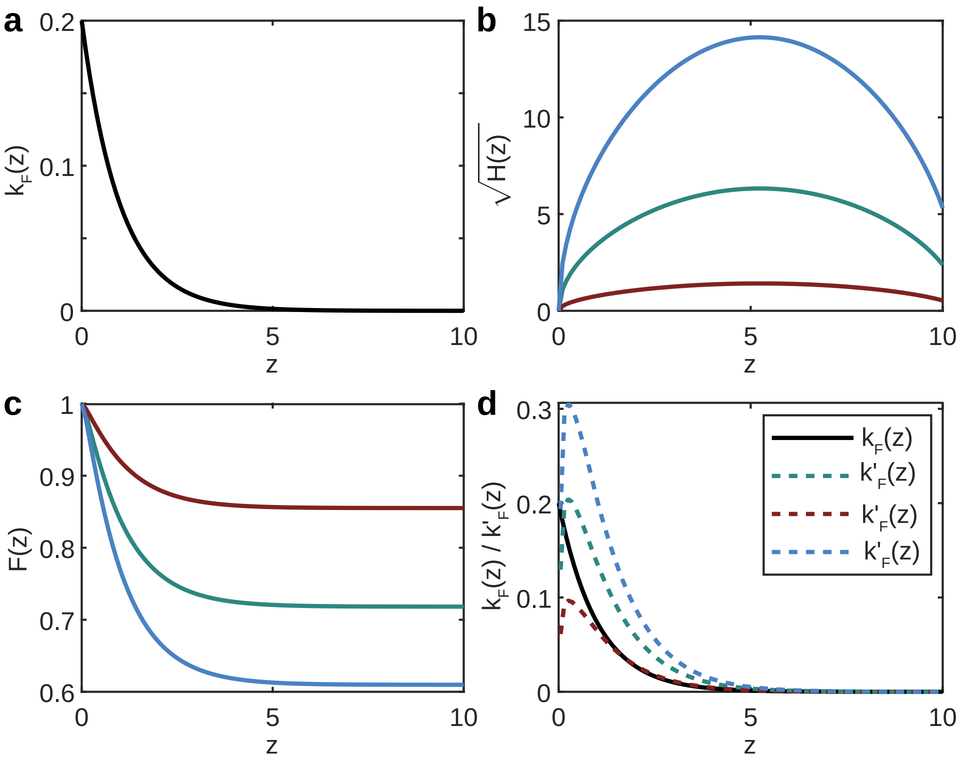

Supplementary Fig. 1 |: Assessing the sulphide dependency of Fe_{HR} decay rate coefficients. For this test, we first prescribe (**a**) a $k_F(t)$ profile (i.e., independent of sulphide concentration, $H(z)$; Eq. S45), and (**b**) three $H(z)$ profiles that span the typical range observed in marine sediments. We then calculate (**c**) Fe_{HR} profiles, $F(z)$, for each $H(z)$ profile using Eq. S44, and we (**d**) extract predicted $k'_F(t)$ profiles from $F(z)$ profiles according to Eq. S46. Resulting $k'_F(t)$ values agree with prescribed $k_F(t)$ to within 30% for all sulphide scenarios, indicating that our choice to ignore sulphide dependency does not introduce significant error in Fe_{HR} decay-rate predictions.

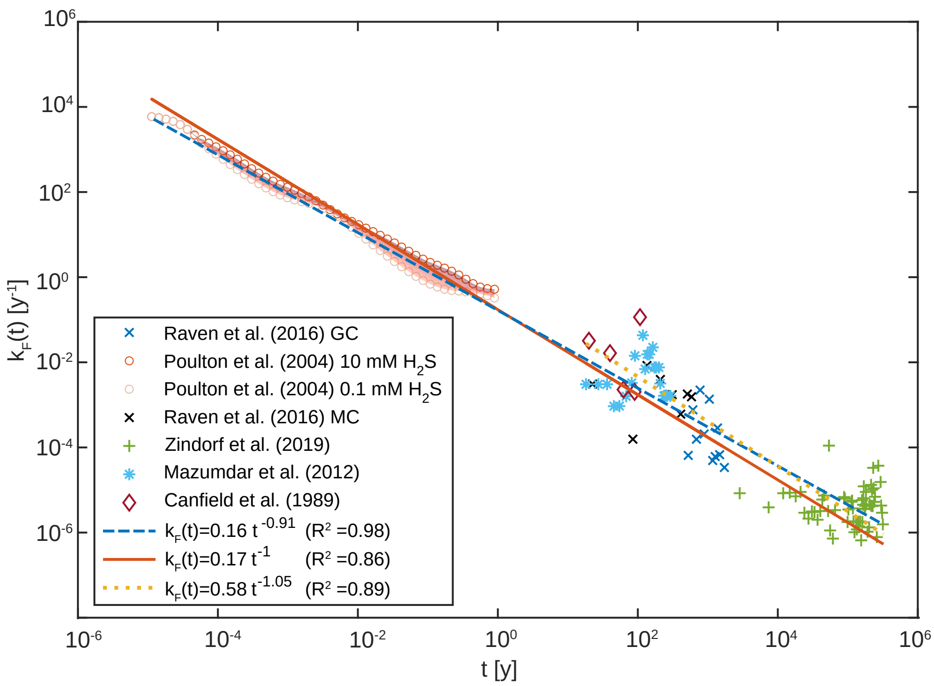

Supplementary Fig. 2 |: Extracting the time-dependent Fe_{HR} decay rate coefficient, $k_F(t)$. Experimental data are from Ref. [111], whereas sedimentary core data are from Refs. [73,94, 128,129]. As detailed in Supplementary Discussion Sec. 2, sulphide concentration-dependent experimental $k_F(t)$ vs. t profiles are calculated for two sulphide concentration scenarios (i.e., 0.1 mM and 10 mM) by assuming a distribution of Fe_{HR} species described in Supplementary Table 1 and an initial time point of t_0 = 60 s. Further, sedimentary sulphide concentration-independent $k_F(t)$ vs. t profiles are calculated using smoothed $F(t)$ observations (converted from z to t using reported sedimentation rates) after excluding core sections that display increasing Fe_{HR} content with depth. We then calculate regression equations in three ways (Eq. S46): (i) using all data with regression slope as a free parameter (blue stippled line; R^2 = 0.98), (ii) using all data while forcing regression slope to −1 (solid red line; R^2 = 0.86), and (iii) using sedimentary data only with regression slope as a free parameter (yellow dotted line; R^2 = 0.89). All model analyses are performed using the option (ii) regression equation (Extended Data Table 1). Abbreviations refer to: "MC" = Multi-corer; "GC" = Gravity-corer.

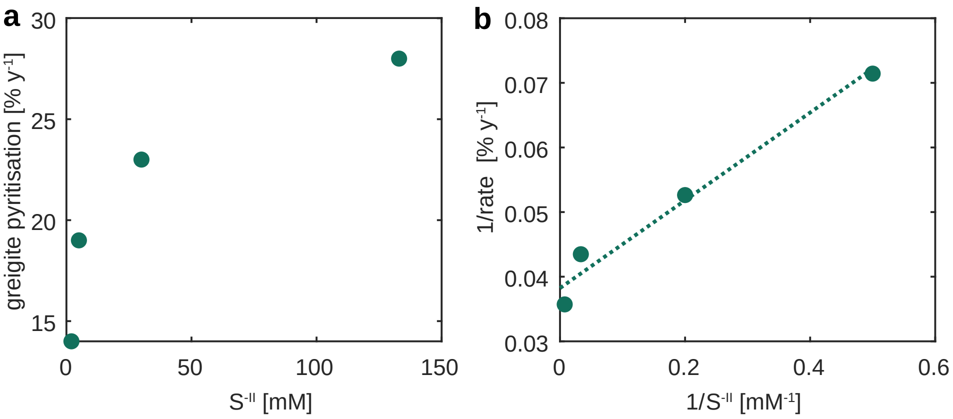

Supplementary Fig. 3 |: Determining the Monod half-velocity constant for sulphide during pyrite formation. (**a**) Sulphide concentration, [S^{-II}] vs. greigite pyritisation rate based on data from Ref.[70]. (**b**) The same data plotted as reciprocals (i.e., 1/[S^{-II}] vs. 1/rate), with geometric mean (GM) regression line (red line). Following the approach of Ref.[67], we use this GM regression to estimate K_H = 1.78 ± 0.33 mM S^{-II} (uncertainty as regression 95% CI). All model analyses were performed using this value of K_H (Extended Data Table 1).

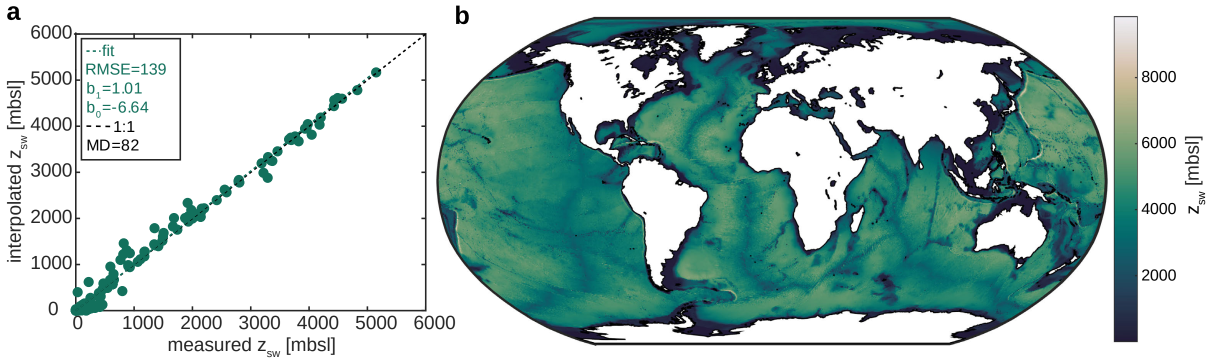

Supplementary Fig. 4 |: **Estimates of ocean water depth, z_{sw}.** (**a**) Comparison of measured vs. predicted[49] z_{sw} values for all available sites included in this study (Supplementary Table 2). Also shown in panel (a) is the 1:1 line (black dotted line) and the geometric mean (GM) regression, including intercept (b_0), slope (b_1), root-mean square error (RMSE), and mean deviation from the 1:1 line (MD) values (green dotted line). (**b**) Globally gridded map of z_{sw} at 25 arc-minute resolution. All model analyses were performed using this map of z_{sw} (Extended Data Table 3). The basemap and coastlines were created using the MATLAB Mapping Toolbox.

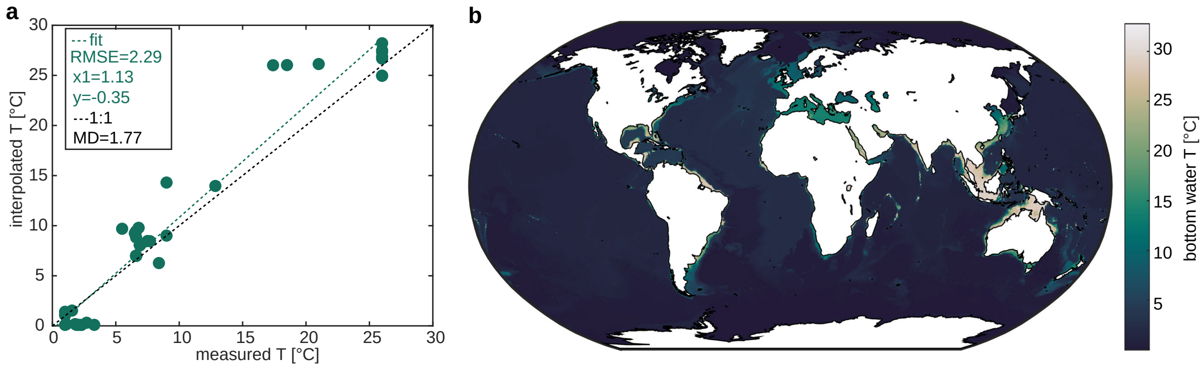

Supplementary Fig. 5 |: Estimates of ocean bottom-water temperature, $T_{\mathbf{sw}}$. (**a**) Comparison of measured vs. predicted[96] T_{sw} values for all available sites included in this study (Supplementary Table 2). Also shown in panel (a) is the 1:1 line (black dotted line) and the geometric mean (GM) regression, including intercept (b_0), slope (b_1), root-mean square error (RMSE), and mean deviation from the 1:1 line (MD) values (green dotted line). (**b**) Globally gridded map of T_{sw} at 25 arc-minute resolution. All model analyses were performed using this map of T_{sw} (Extended Data Table 3). The basemap and coastlines were created using the MATLAB Mapping Toolbox.

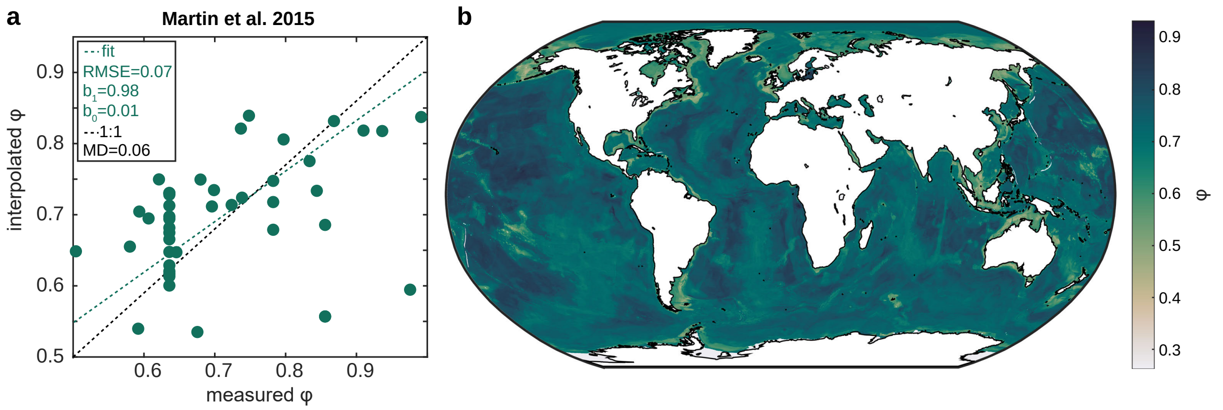

Supplementary Fig. 6 |: **Estimates of sediment porosity,** ϕ. (**a**) Comparison of measured vs. predicted[26] ϕ values for all available sites included in this study (Supplementary Table 2). Also shown in panel (a) is the 1:1 line (black dotted line) and the geometric mean (GM) regression, including intercept (b_0), slope (b_1), root-mean square error (RMSE), and mean deviation from the 1:1 line (MD) values (green dotted line). (**b**) Globally gridded map of ϕ at 25 arc-minute resolution. All model analyses were performed using this map of ϕ (Extended Data Table 3). The basemap and coastlines were created using the MATLAB Mapping Toolbox.

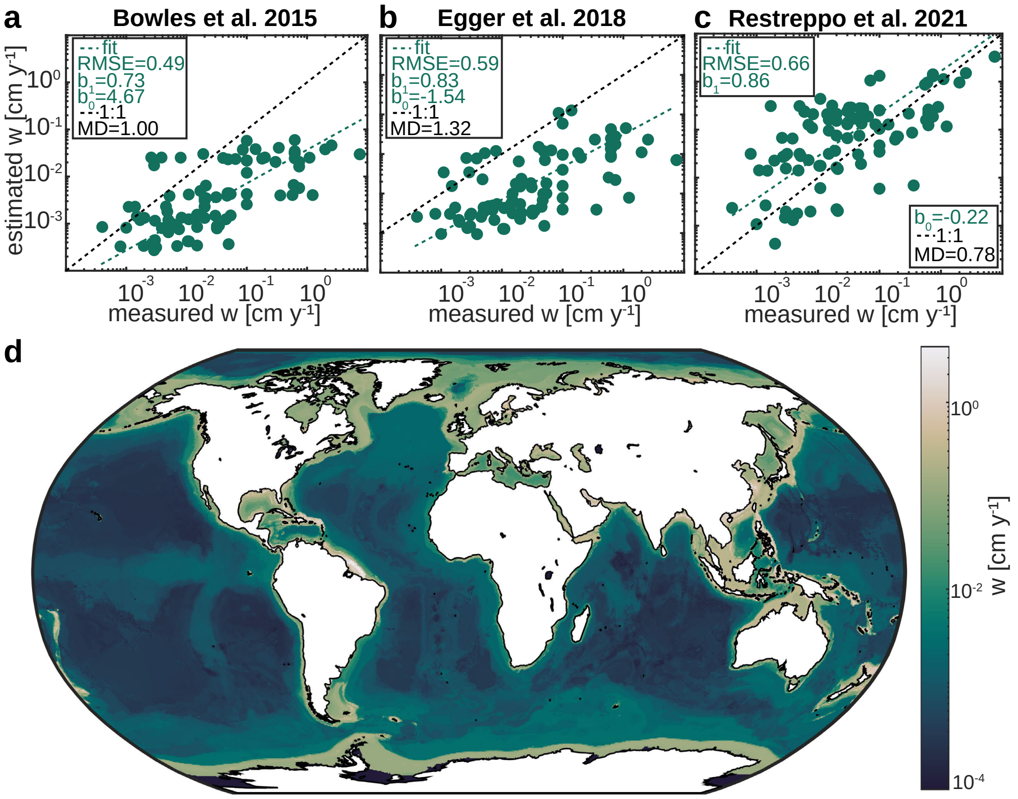

Supplementary Fig. 7 |: Estimates of sedimentation rate, w. Comparison of measured vs. predicted w values for all available sites included in this study (Supplementary Table 2) using predicted sedimentation rates from (**a**) Ref.[36], (**b**) Ref.[50], and (**c**) Ref.[28]. Also shown for each panel is the 1:1 line (black dotted line) and the geometric mean (GM) regression, including intercept (b_0), slope (b_1), root-mean square error (RMSE), and mean deviation from the 1:1 line (MD) values (green dotted line). (**d**) Globally gridded map of w at 25 arc-minute resolution using data from Ref.[28]. All model analyses were performed using this map of w (Extended Data Table 3). The basemap and coastlines were created using the MATLAB Mapping Toolbox.

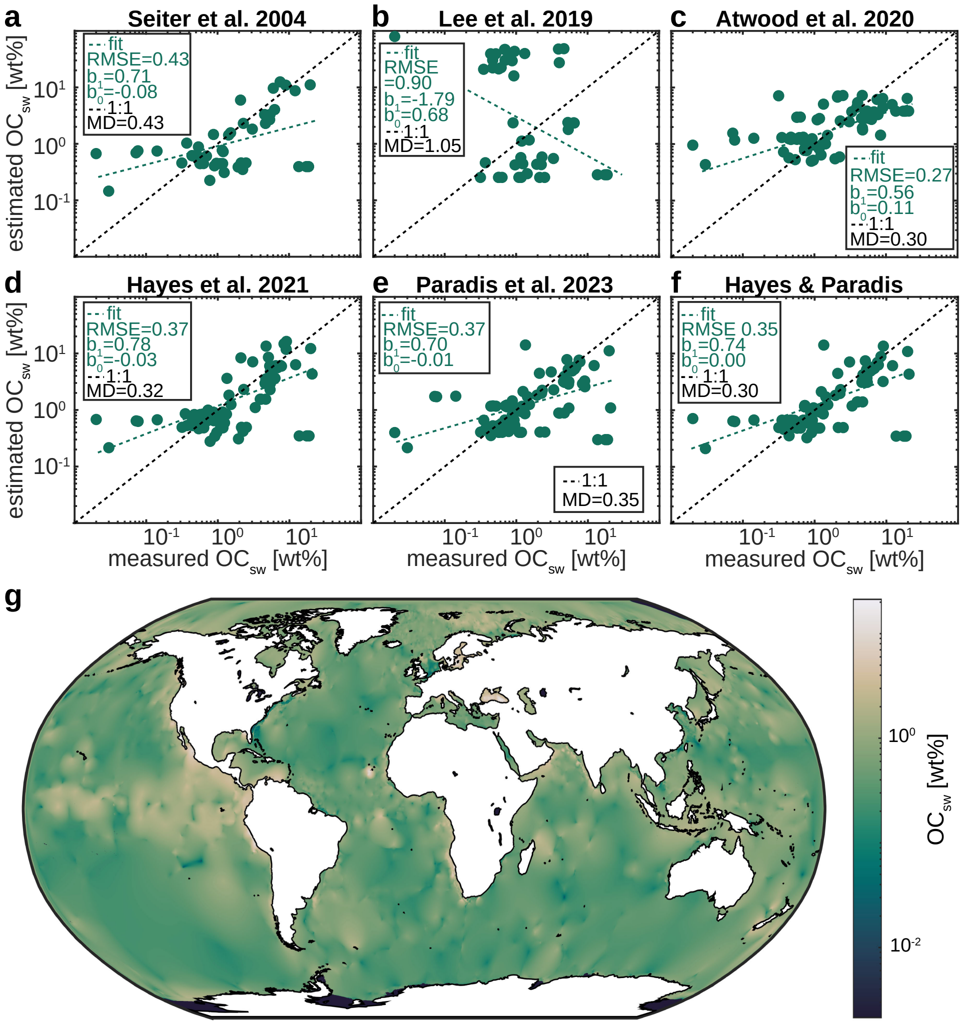

Supplementary Fig. 8 |: **Estimates of OC content at the sediment-water interface, G_{sw}.** Comparison of measured vs. predicted G_{sw} values for all available sites included in this study (Supplementary Table 2) using predicted OC contents from (**a**) Ref.[140], (**b**) Ref.[141], and (**c**) Ref.[142], as well as interpolated OC contents using the global datasets of (**d**) Ref.[25], (**e**) Ref.[24], (**f**) and a combination of Refs.[24,25]. Also shown for each panel is the 1:1 line (black dotted line) and the geometric mean (GM) regression, including intercept (b_0), slope (b_1), root-mean square error (RMSE), and mean deviation from the 1:1 line (MD) values (green dotted line). (**d**) Globally gridded map of G_{sw} at 25 arc-minute resolution using interpolated data from Refs.[24,25]. All model analyses were performed using this map of G_{sw} (Extended Data Table 3). The basemap and coastlines were created using the MATLAB Mapping Toolbox.

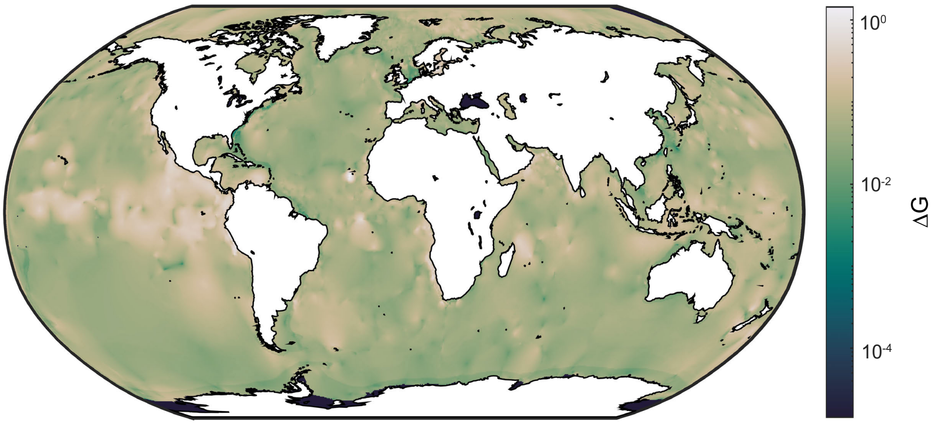

Supplementary Fig. 9 |: Difference in OC content at the sediment-water interface and at the start of the sulphate reduction zone, ΔG. Global map of $\Delta G = G_0 - G_{\mathrm{sw}}$ at 25 arc-minute resolution. G_0 is calculated by Eq. S17 using w, G_{sw}, z_{MLD}, and $k_{G_{\mathrm{sw}}}$ maps shown in Supplementary Figs. 7 and 8, and Supplementary Figs. 14 and 16. The basemap and coastlines were created using the MATLAB Mapping Toolbox.

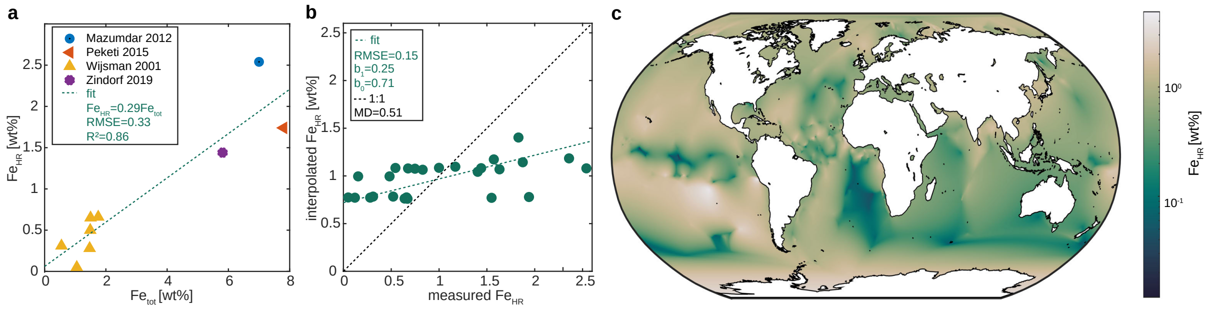

Supplementary Fig. 10 |: Estimates of $\mathrm{Fe_{HR}}$ content at the sediment-water interface, F_{sw}. (**a**) Measured $\mathrm{Fe_{HR}}$ content as a function of total iron content using available data from Refs.[128,129,143,144]. Also shown in panel (a) is the geometric mean (GM) regression, including root-mean square error (RMSE) and R^2 values (green solid line). (**b**) Comparison of measured vs. predicted F_{sw} values for all sites included in this study that report total iron contents[25, 99–102] (Supplementary Table 2), after correcting to $\mathrm{Fe_{HR}}$ using the correlation determined in panel (a). Also shown in panel (b) is the 1:1 line (black dotted line) and the geometric mean (GM) regression, including intercept (b_0), slope (b_1), root-mean square error (RMSE), and mean deviation from the 1:1 line (MD) values (green dotted line). (**c**) Globally gridded map of F_{sw} at 25 arc-minute resolution using interpolated data from Refs.[25, 99–102]. All model analyses were performed using this map of F_{sw} (Extended Data Table 3). The basemap and coastlines were created using the MATLAB Mapping Toolbox.

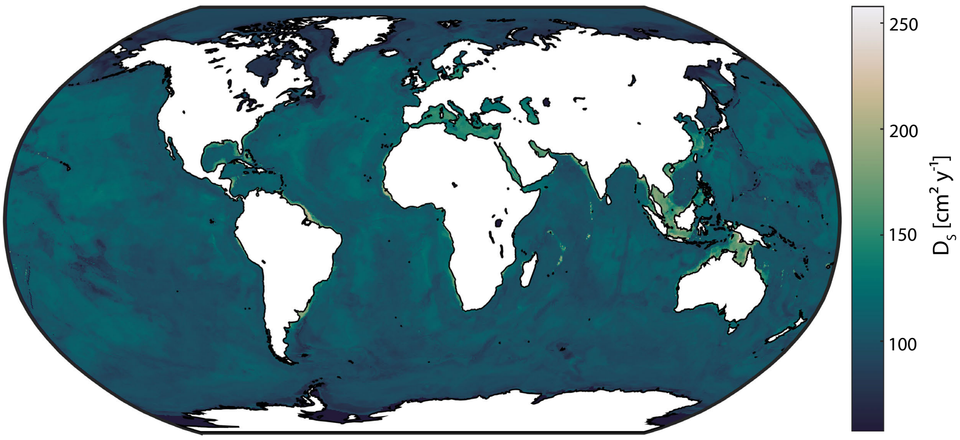

Supplementary Fig. 11 |: **Estimates of sulphate diffusivity,** D_S. Globally gridded map of D_S at 25 arc-minute resolution calculated using Eqs. S167-S168 and global T_{sw} and ϕ maps shown in Supplementary Figs. 5-6. All model analyses were performed using this map of D_S (Extended Data Table 3). The basemap and coastlines were created using the MATLAB Mapping Toolbox.

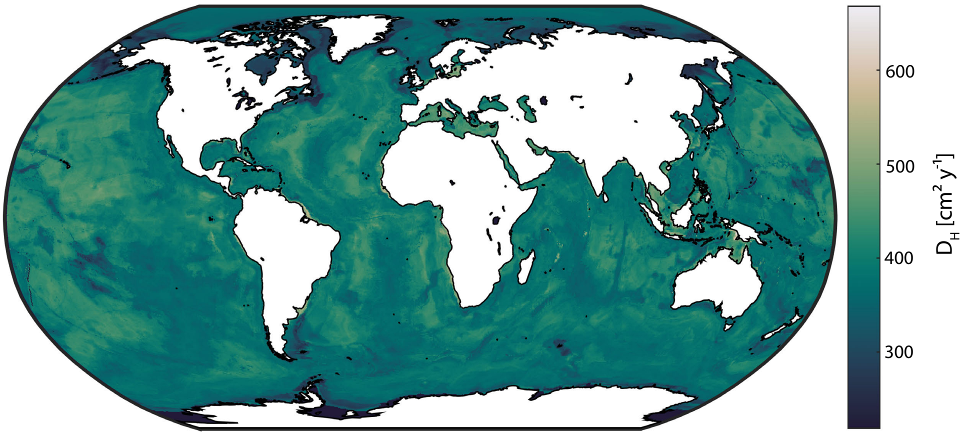

Supplementary Fig. 12 |: Estimates of sulphide diffusivity, D_H. Globally gridded map of D_H at 25 arc-minute resolution calculated using Eqs. S169-S170 and global T_{sw} and ϕ maps shown in Supplementary Figs. 5-6. All model analyses were performed using this map of D_H (Extended Data Table 3). The basemap and coastlines were created using the MATLAB Mapping Toolbox.

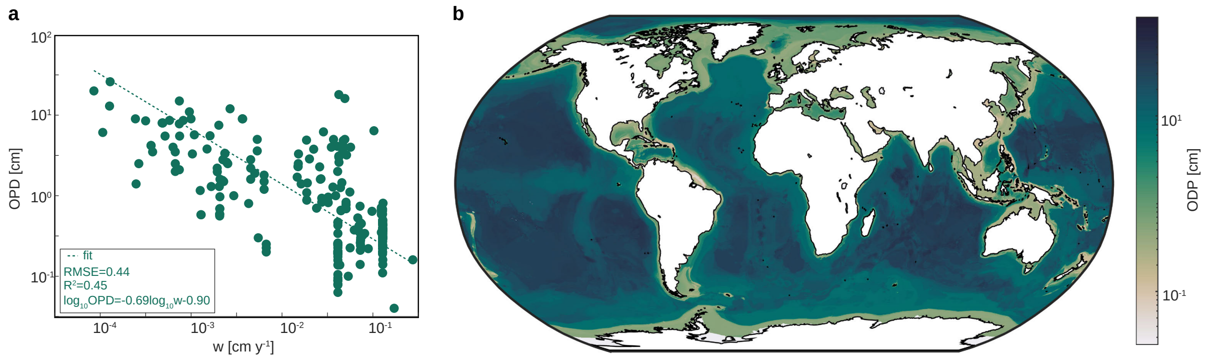

Supplementary Fig. 13 |: **Estimates of oxygen penetration depth,** $\mathbf{z_{OPD}}$. (**a**) Measured z_{OPD} as a function of sedimentation rate, w, using available data from Ref.[105]. Also shown in panel (a) is the geometric mean (GM) regression, including root-mean square error (RMSE) and R^2 values (green dotted line). (**b**) Globally gridded map of z_{OPD} at 25 arc-minute resolution using the regression relationship determined in panel (a) and global w map show in Supplementary Fig. 7. All model analyses were performed using this map of z_{OPD} (Extended Data Table 3). The basemap and coastlines were created using the MATLAB Mapping Toolbox.

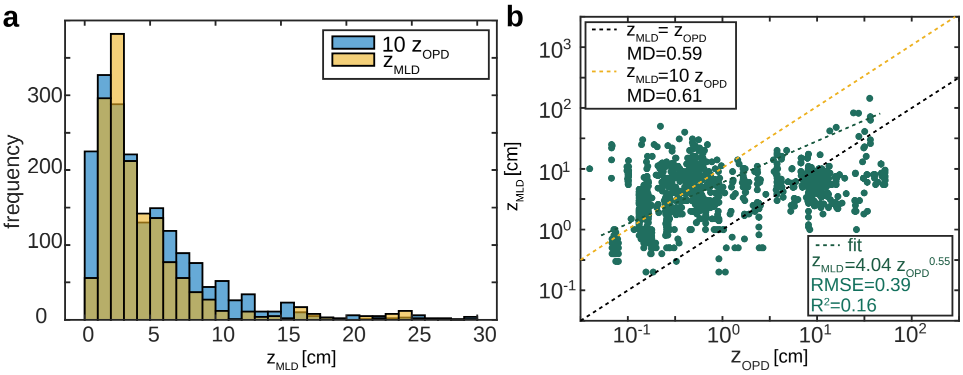

Supplementary Fig. 14 |: Estimates of mixed layer depth, z_{MLD}. (**a**) Histogram of z_{MLD} using data from Ref.[45]. Also shown in panel (a) is a histogram of z_{MLD} assuming $z_{\mathrm{MLD}} = 10 \times z_{\mathrm{OPD}}$ using the global z_{OPD} map shown in Supplementary Fig. 13. (**b**) Measured z_{MLD} as a function of z_{OPD} using available data from Ref.[45] and the global z_{OPD} map shown in Supplementary Fig. 13 (interpolated to each sampling location in Ref.[45]). Also shown in panel (b) is the geometric mean (GM) regression including root-mean square error (RMSE) and R^2 values (green dotted line), the $z_{\mathrm{MLD}} = z_{\mathrm{OPD}}$ line (i.e., 1:1; black dotted line), and the $z_{\mathrm{MLD}} = 10 \times z_{\mathrm{OPD}}$ line (yellow dotted line) including mean deviation of the data from each respective line (MD). In both panels, results support our estimate that $z_{\mathrm{MLD}} \sim 10 \times z_{\mathrm{OPD}}$, particularly for shallower z_{OPD} values representative of continental shelves and slopes where the majority of pyrite is formed globally. In contrast, z_{OPD} approaches and even exceeds z_{MLD} in the open abyssal ocean where pyrite formation is minimal.

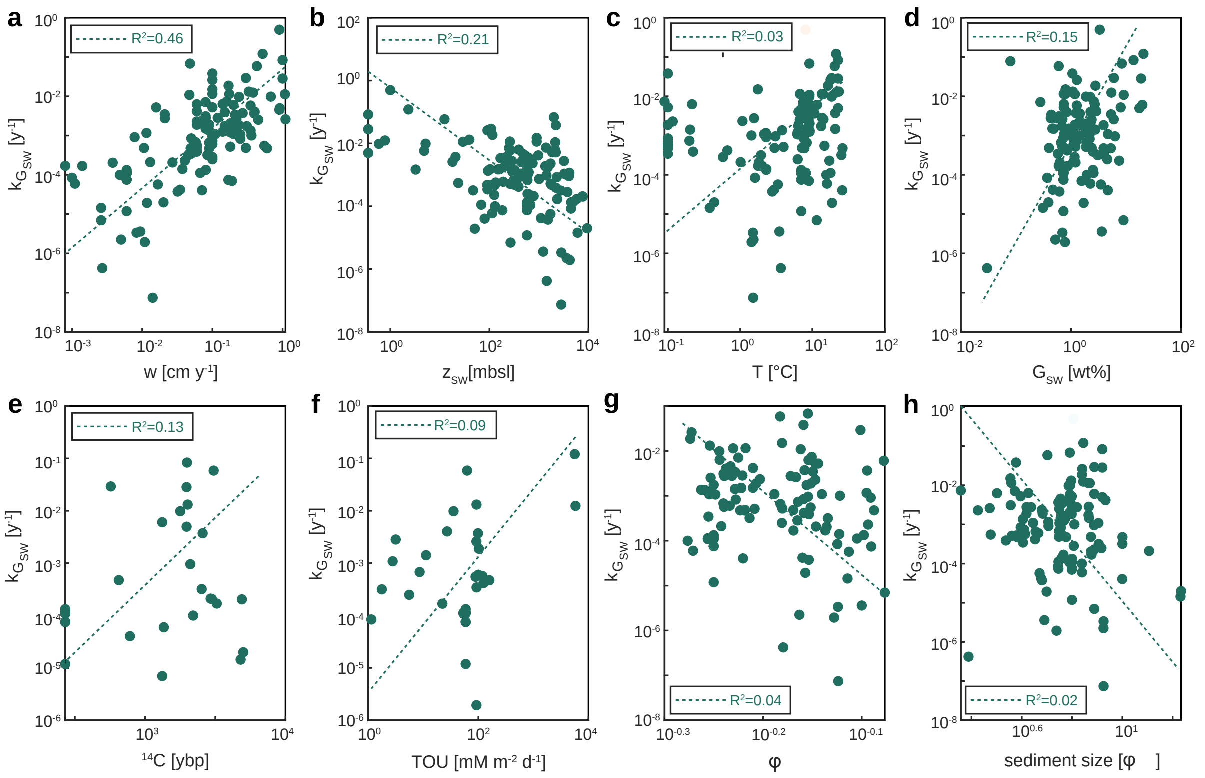

Supplementary Fig. 15 |: Predicting $k_{G_{\mathrm{sw}}}$ using various environmental variables. $k_{G_{\mathrm{sw}}}$ is calculated using OC profiles from diverse global environmental settings. Specifically, we fit Eq. S3 to 138 OC profiles from the MOSAIC database (Ref.[24]). We then regress $k_{G_{\mathrm{sw}}}$ using geometric mean (GM) regression against (**a**) sedimentation rate measured independently at each site (R^2 = 0.46; p-value = 8.2×10^{-19}); (**b**) water depth, z_{SW}, interpolated from a globally gridded map[49] to the GPS coordinates of the sites (R^2 = 0.21; p-value = 8.1×10^{-8}); (**c**) bottom-water temperature, z_{SW}, interpolated from a globally gridded map[96] to the GPS coordinates of the sites (R^2 = 0.03; p-value = 0.07); (**d**) OC content at the sediment-water interface, G_{sw}, measured at each site[24] (R^2 = 0.15; p-value = 6.6×10^{-6}); (**e**) radiocarbon (^{14}C) age measured at each site[24] (R^2 = 0.13; p-value = 0.04); (**f**) total oxygen uptake interpolated from a globally scattered dataset[105] to the GPS coordinates of the sites (R^2 = 0.09; p-value = 0.08); (**g**) porosity, ϕ, interpolated from a globally gridded map[26] to the GPS coordinates of the sites (R^2 = 0.04; p-value = 0.05); and (**h**) sediment particle size interpolated from a globally scattered dataset[159] to the GPS coordinates of the sites (R^2 = 0.02; p-value = 0.10). From regression statistics, sedimentation rate emerges as the strongest predictor. When including multiple predictors, sedimentation rate consistently remains the most significant statistical predictor ($R^2 \leq 0.2$; p-value ≥ 0.03 for every variable when including sedimentation rate as a second predictor). All p-values correspond to the t-statistic from two-sided hypothesis tests.

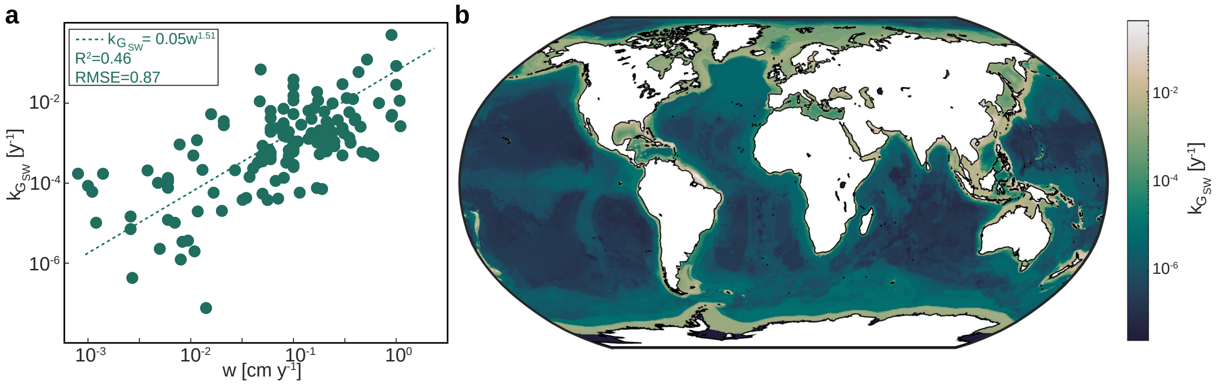

Supplementary Fig. 16 |: Estimates of OC reactivity at the sediment-water interface, $k_{G_{\mathbf{sw}}}$. (**a**) Measured $k_{G_{\rm sw}}$ as a function of sedimentation rate, w, using available data from Ref.[24]. Also shown in panel (a) is the geometric mean (GM) regression, including root-mean square error (RMSE) and R^2 values (green dotted line). (**b**) Globally gridded map of $k_{G_{\rm sw}}$ at 25 arc-minute resolution using the regression relationship determined in panel (a) and global w map show in Supplementary Fig. 7. All model analyses were performed using this map of $z_{\rm OPD}$ (Extended Data Table 3). The basemap and coastlines were created using the MATLAB Mapping Toolbox.

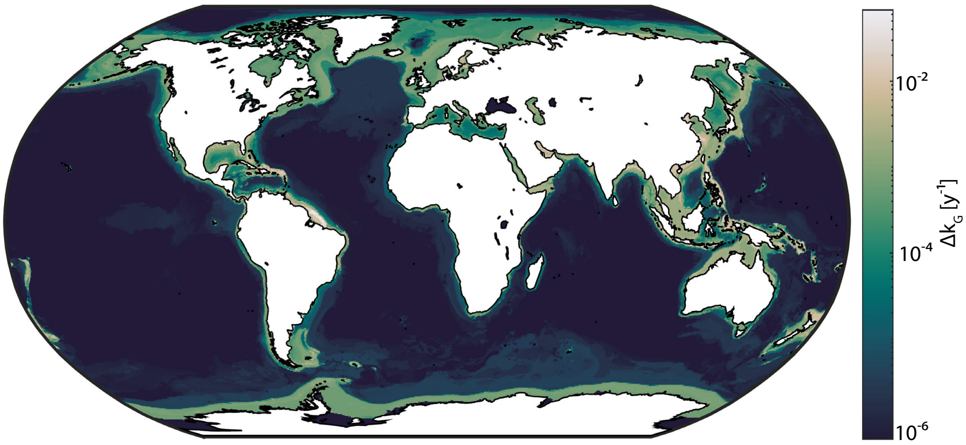

Supplementary Fig. 17 |: Difference in OC reactivity at the sediment-water interface and at the start of the sulphate reduction zone, Δk_G. Global map of Δk_G at 25 arc-minute resolution calculated using Eq. S174 using z_{MLD} and $k_{G_{\mathrm{sw}}}$ maps shown in Supplementary Figs. 14 and 16. The basemap and coastlines were created using the MATLAB Mapping Toolbox.

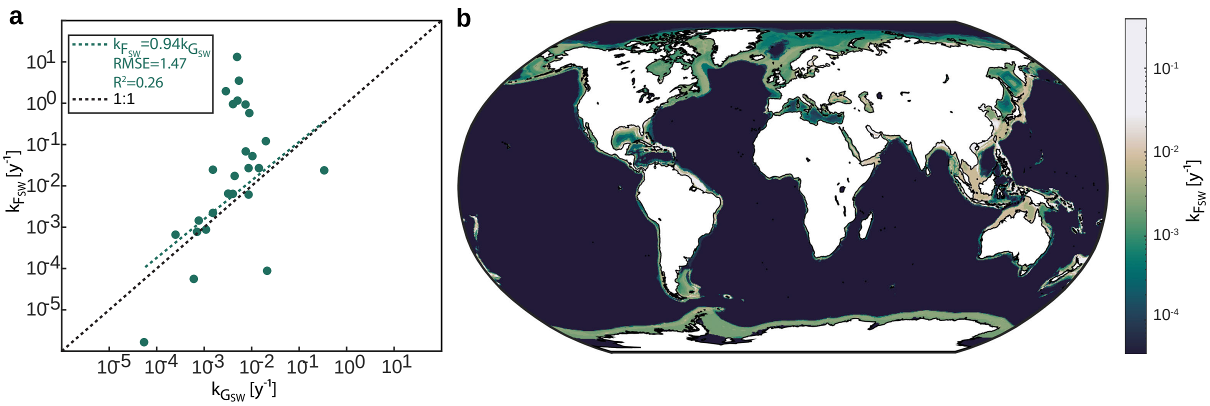

Supplementary Fig. 18 |: **Estimates of $\mathbf{Fe_{HR}}$ reactivity at the sediment-water interface, $k_{F_{\mathbf{sw}}}$.** (**a**) Measured $k_{F_{\mathrm{sw}}}$ as a function of OC reactivity at the sediment-water interface, $k_{G_{\mathrm{sw}}}$. Inputted $k_{F_{\mathrm{sw}}}$ values are determined by fitting Eq. S50 to available reactive iron profiles[73,94,128,129] (Supplementary Table 2; see also Supplementary Fig. 2). Inputted $k_{G_{\mathrm{sw}}}$ values are taken from the global map shown in Supplementary Fig. 16 (interpolated to the GPS coordinates of $k_{F_{\mathrm{sw}}}$-containing profiles). Also shown in panel (a) is the 1:1 line (black dotted line) and geometric mean (GM) regression, including root-mean square error (RMSE) and R^2 values (green dotted line). (**b**) Globally gridded map of $k_{F_{\mathrm{sw}}}$ at 25 arc-minute resolution, assuming $k_{F_{\mathrm{sw}}} = k_{G_{\mathrm{sw}}}$, as predicted by the regression in panel (a). All model analyses were performed using this map of $k_{F_{\mathrm{sw}}}$ (Extended Data Table 3). The basemap and coastlines were created using the MATLAB Mapping Toolbox.

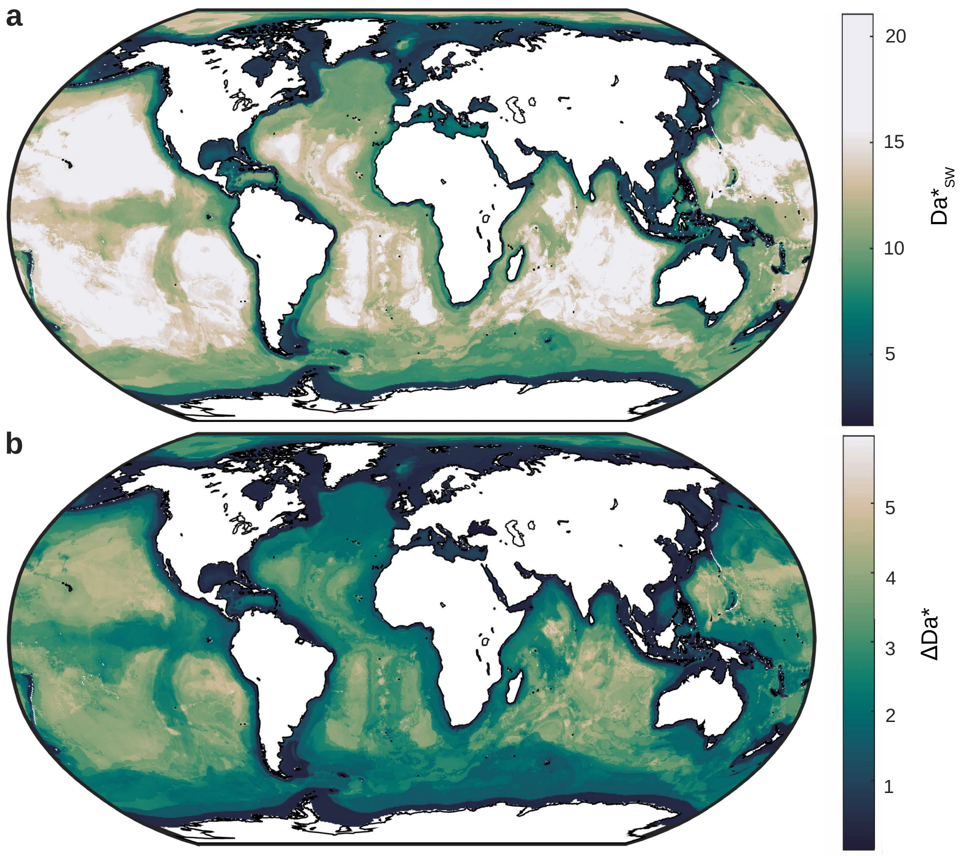

Supplementary Fig. 19 |: Estimates of the modified Damköhler number, Da*. (**a**) Globally gridded map of Da* at the sediment-water interface at 25 arc-minute resolution calculated by Eq. S115 using w, D_S, and $k_{G_{\mathrm{sw}}}$ maps shown in Supplementary Figs. 7, 11, and 16. (**b**) Global map of the difference in Da* at the sediment-water interface and at the start of the sulphate reduction zone, ΔDa*, at 25 arc-minute resolution calculated by Eq. S177 using w, D_S, and Δk_G maps shown in Supplementary Figs. 7, 11, and 17. The basemap and coastlines were created using the MATLAB Mapping Toolbox.

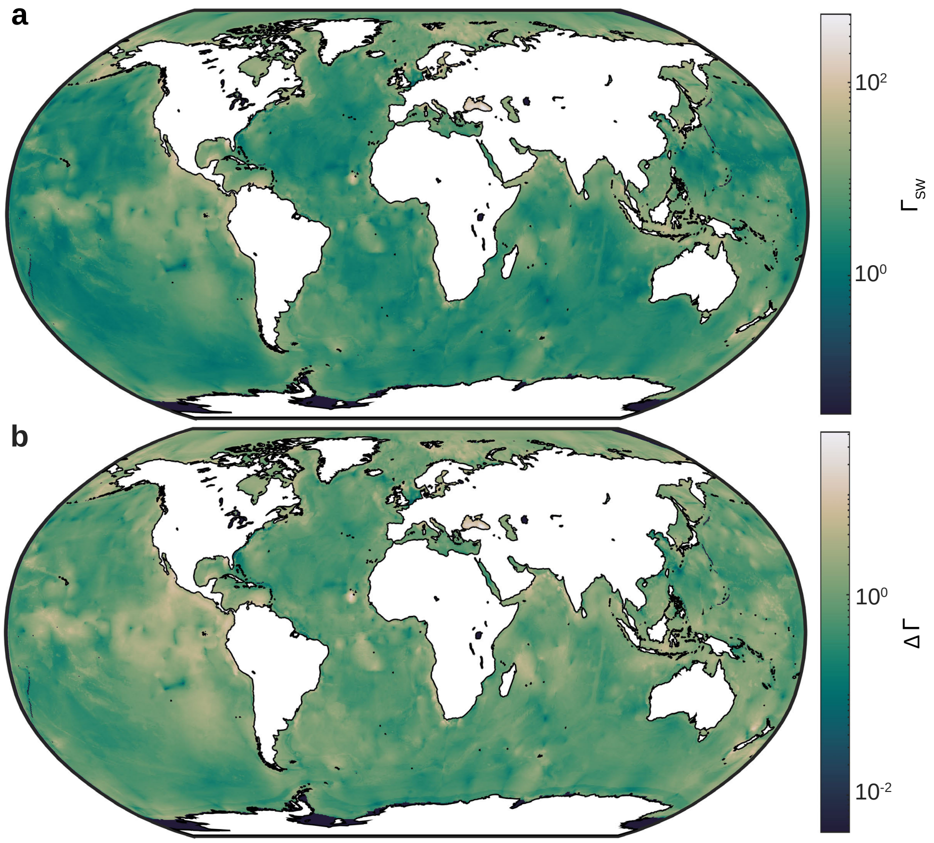

Supplementary Fig. 20 |: **Estimates of the non-dimensional OC content,** Γ. (**a**) Globally gridded map of OC content at the sediment-water interface, Γ_{sw}, at 25 arc-minute resolution calculated by Eq. S117 using the modern ocean sulphate concentration (i.e., $28\,\mathrm{mM}\,\mathrm{SO}_4^{2-}$) and G_{sw} map shown in Supplementary Fig. 8. (**b**) Global map of the difference in Γ at the sediment-water interface and at the start of the sulphate reduction zone, $\Delta\Gamma$, at 25 arc-minute resolution calculated by Eq. S179 using the ΔG map shown in Supplementary Fig. 9. The basemap and coastlines were created using the MATLAB Mapping Toolbox.

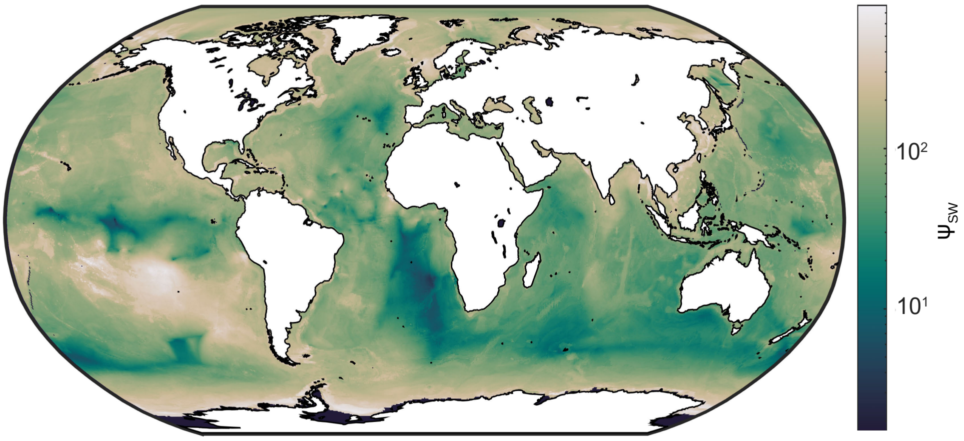

Supplementary Fig. 21 |: Estimates of the modified non-dimensional $\mathbf{Fe_{HR}}$ content at the sediment-water interface, $\boldsymbol{\Psi_{sw}}$. Globally gridded map of Ψ_{sw} at 25 arc-minute resolution calculated by Eq. S127 using the modern ocean sulphate concentration (i.e., 28 mM SO_4^{2-}) and F_{sw} map shown in Supplementary Fig. 10. The basemap and coastlines were created using the MATLAB Mapping Toolbox.

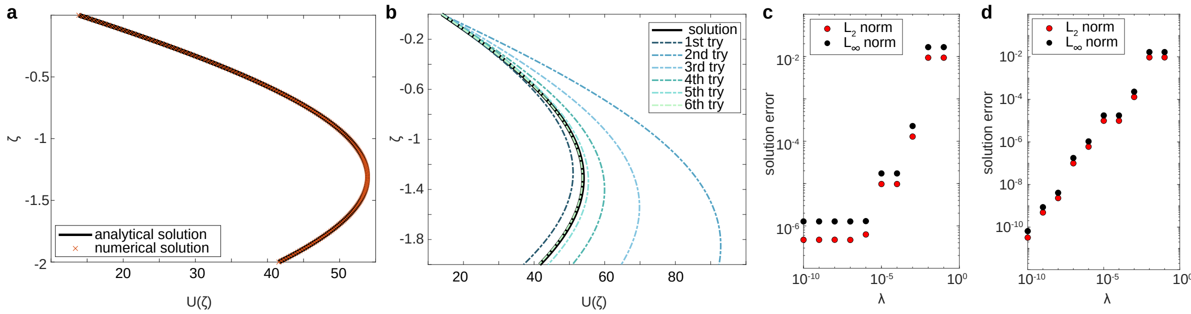

Supplementary Fig. 22 |: Assessing the shooting algorithm. To quantify performance, we define an arbitrary function with a known analytical solution, $U(\zeta)$, that depends on non-dimensional depth, ζ, and we estimate the error when finding a numerical solution. (**a**) Comparison of exact analytical solution with the numerical solution found by Matlab's ode45 solver using a shooting function to estimate the initial sulphate derivative. (**b**) Example highlighting how guesses are iteratively refined using a bisection function. Solution error, quantified as both L_2 (Eq. S180) and L_∞ (Eq. S182) norms, as a function of the shooting algorithm error tolerance threshold, λ, for two ode45 solver error tolerances, (**c**) 10^{-12} and (**d**) 10^{-1}. For the shooting error value of $\lambda = 10^{-6}$ chosen here, we expect L_2 and L_∞ errors of $\sim 10^{-6}$, which we interpret as the numerical error characteristic of our shooting algorithm.

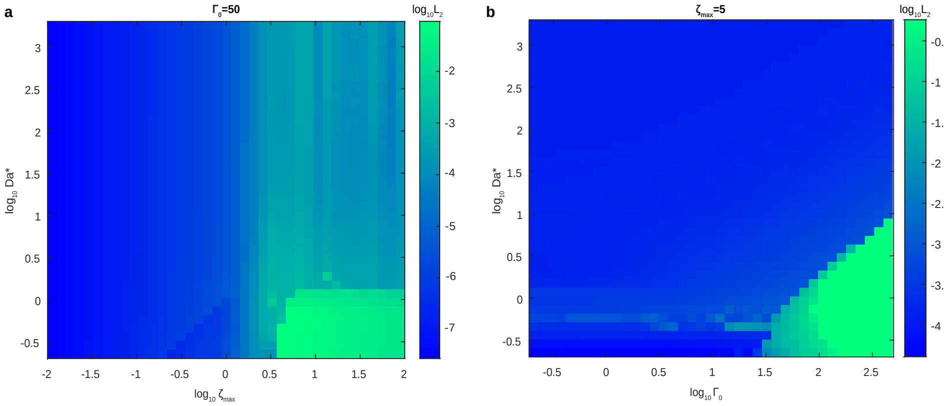

Supplementary Fig. 23 |: **Sulphate concentration solution stability field**. Both panels display numerical solver and implemented equation accuracy, quantified using the L_2 norm (Eq. S180) and calculated using the method of manufactured solutions (Supplementary Discussion Sec. 6; Eqs. S189–S192). Stability as a function of (**a**) $\zeta_{\max}$, the maximum non-dimensional depth for the model solution, vs. Da^* and (**b**) Γ_0, the OC content at the top of the sulphidic anoxic zone, vs. Da^*. Panel (a) assumes $\Gamma_0 = 50$, whereas panel (b) assumes $\zeta_{\max} = 5$; both are typical values observed in the modern ocean. White areas (if present) indicate $L_2 > 1$, which we interpret as regions of solver instability. The absence of white areas indicates that our sulphate concentration model is stable over the entire parameter space of interest.

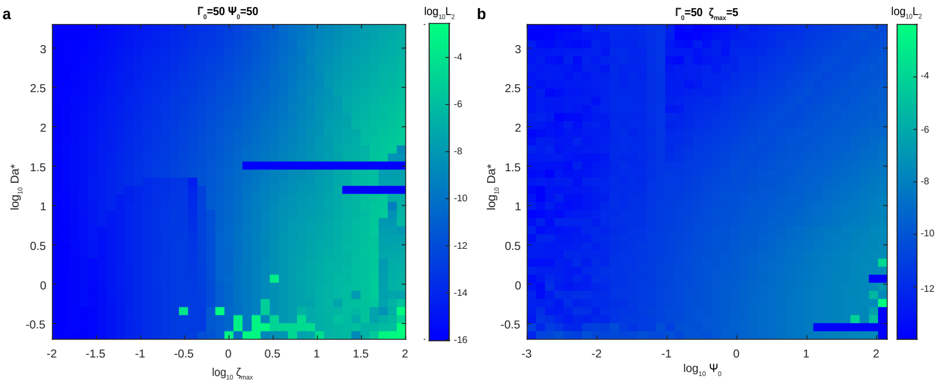

Supplementary Fig. 24 |: Sulphide concentration solution stability field. Both panels display numerical solver and implemented equation accuracy, quantified using the L_2 norm (Eq. S180) and calculated using the method of manufactured solutions (Supplementary Discussion Sec. 6; Eqs. S193–S196). Stability as a function of (**a**) ζ_{max}, the maximum non-dimensional depth for the model solution, vs. Da* and (**b**) Ψ_0, the Fe_{HR} content at the top of the sulphidic anoxic zone, vs. Da*. Both panels assume $\Gamma_0 = 50$, whereas panel (a) assumes $\Psi_0 = 50$ and panel (b) assumes $\zeta_{\text{max}} = 5$; all are typical values observed in the modern ocean. White areas (if present) indicate $L_2 > 1$, which we interpret as regions of solver instability. The absence of white areas indicates that our sulphide concentration model is stable over the entire parameter space of interest.

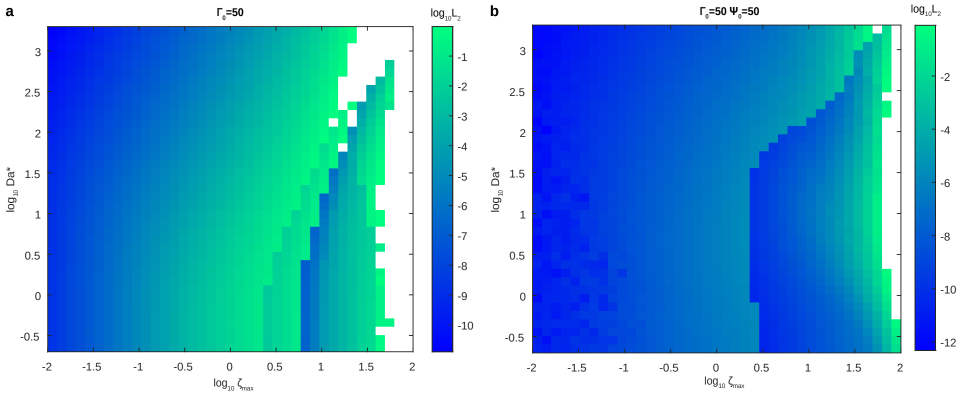

Supplementary Fig. 25 |: Sulphate and sulphide isotopic composition solution stability field. Both panels display numerical solver and implemented equation accuracy, quantified using the L_2 norm (Eq. S180) and calculated using the method of manufactured solutions (Supplementary Discussion Sec. 6). Stability as a function of $\zeta_{\max}$, the maximum non-dimensional depth for the model solution, vs. Da* for (**a**) sulphate isotopic composition (Eqs. S197–S207) and (**b**) sulphide isotopic compositions (Eqs. S208–S209). Both panels assume $\Gamma_0 = 50$, whereas panel (b) also assumes $\Psi_0 = 50$; both are typical values observed in the modern ocean. White areas (if present) indicate $L_2 > 1$, which we interpret as regions of solver instability. The absence of white areas when $\zeta_{\max} \leq 10$ indicates that our sulphate concentration model is stable over the entire parameter space of interest (i.e., since we solve our model with $\zeta_{\max} \leq 10$ always).

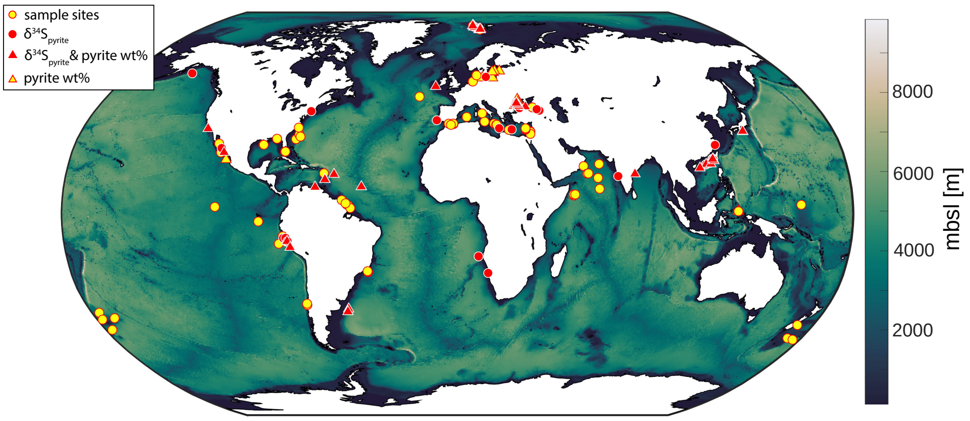

Supplementary Fig. 26 |: Map of all sites included in our literature compilation. Sites are separated into those with available pyrite contents (triangles) and those with available pyrite isotopic compositions (red markers). Sites containing other sulphur species contents/concentrations or isotopic compositions, but no pyrite data, are shown as yellow circles. Data compiled from Refs.[73, 53,57,58,77, 128,129,143,144,161,164–213] (Supplementary Data; Supplementary Table 2). The basemap and coastlines were created using the MATLAB Mapping Toolbox.

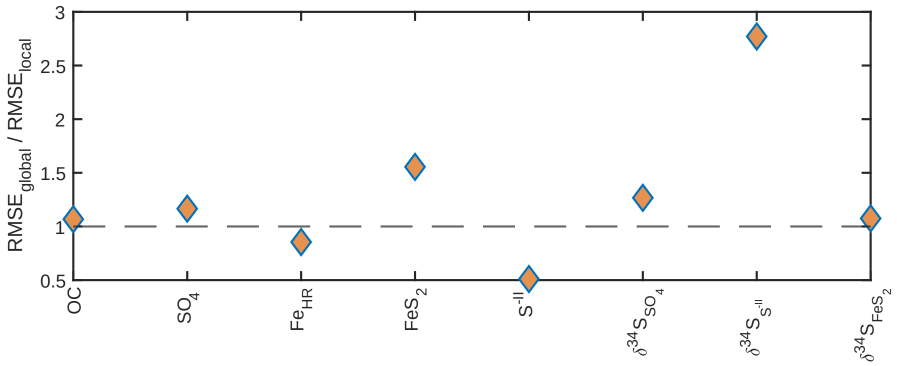

Supplementary Fig. 27 |: Comparison of model-data misfit root-mean square error (RMSE; Supplementary Table 2) for local and global estimated boundary conditions. For each variable, median global RMSE values (i.e., those determined when comparing observations to predictions using boundary conditions from globally gridded datasets) are normalised to median local RMSE values (i.e., those determined when comparing observations to predictions using measured boundary conditions at each coring location). Values greater than unity indicate poorer model performance with globally estimated boundary conditions for the respective species and *vice versa*.

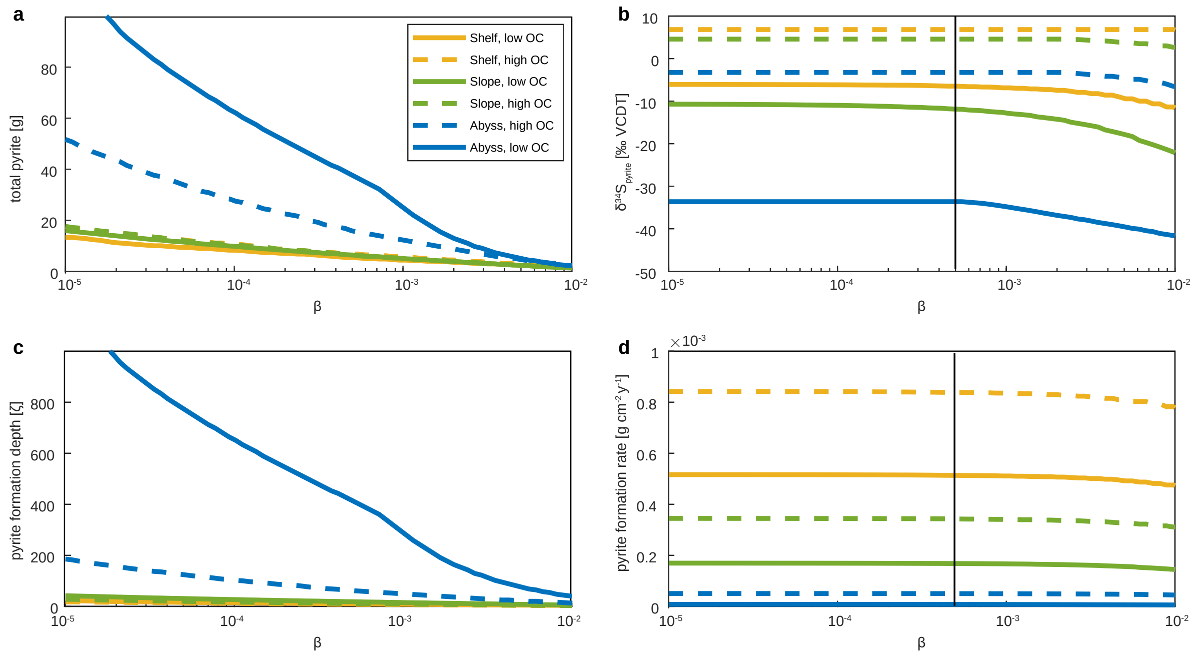

Supplementary Fig. 28 |: Assessing the influence of pyrite formation depth threshold, β. Calculated (**a**) total pyrite content in the sulphidic anoxic zone; (**b**) average isotopic composition of all pyrite in the sulphidic anoxic zone; (**c**) maximum formation depth, ζ_{max} (Eq. 51), defining the lower-boundary of the sulphidic anoxic zone; and (**d**) rate of pyrite formation within the sulphidic anoxic zone for characteristic profiles as functions of threshold value. Profiles are separated into those representing shelf, slope, and abyssal settings with high (>1 wt %) and low (≤1 wt %) OC content. To estimate extremes, we use the 5th percentile of OC content for low-OC conditions and the 95th percentile of OC content for high-OC conditions; all other boundary conditions are taken as the median for the respective bathymetric region (see Supplementary Discussion Sec. 8 for details). Although total pyrite content and ζ_{max} continue to increase with decreasing β by definition, pyrite isotopic composition and formation rate stabilise in all profiles for $\beta \leq 5{\times}10^{-4}$ (vertical black line). We therefore choose this value for all global model calculations performed here.

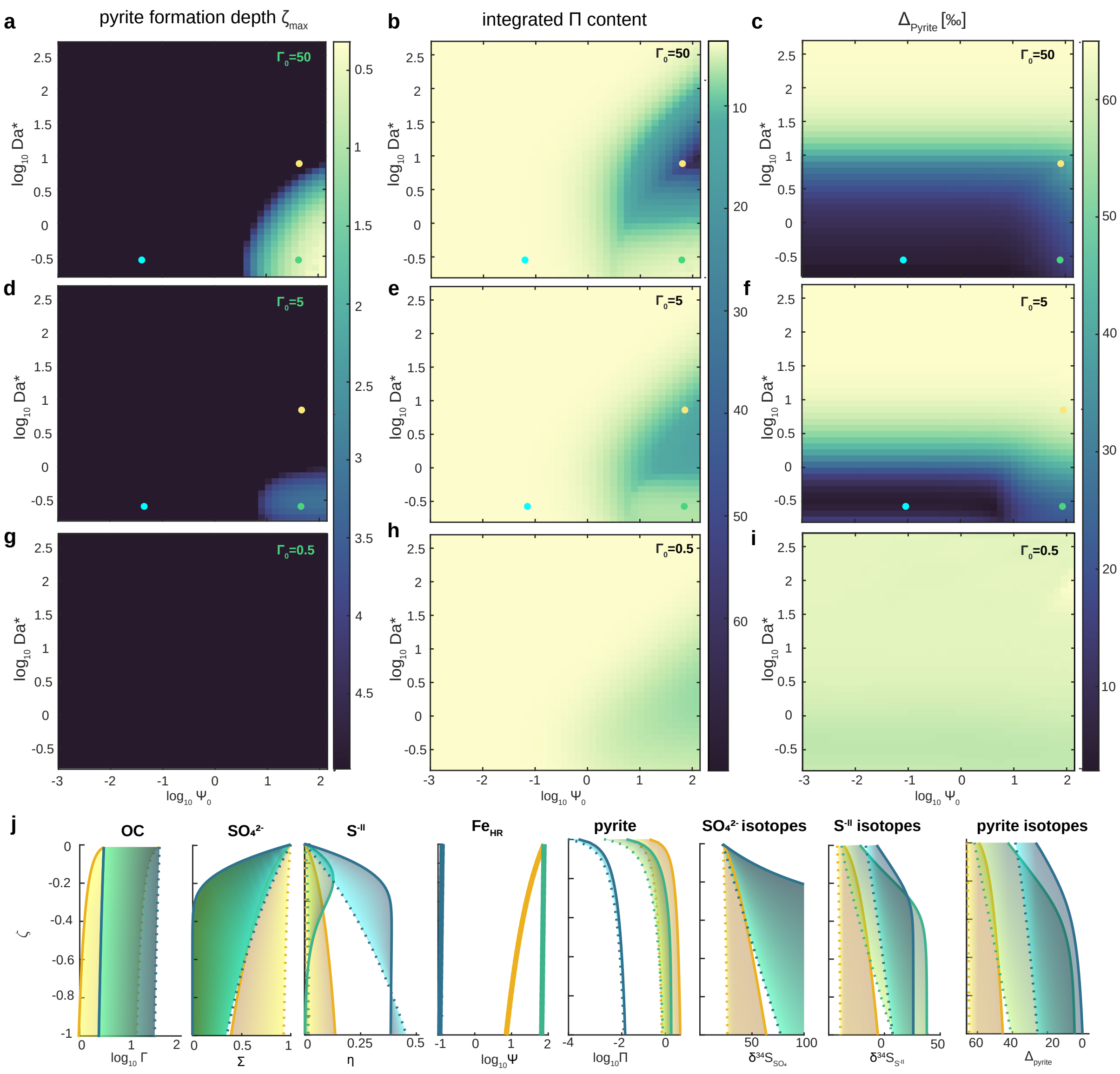

Supplementary Fig. 29 |: Assessing the importance of reactive iron content, Ψ_0. Parameter-space heat maps showing how pyrite parameters vary as functions of Ψ_0 and Da*: (**a**, **d**, **g**) pyrite formation depth, $\zeta_{\max}$ (assuming a threshold value of $\beta = 5 \times 10^{-4}$); (**b**, **e**, **h**) pyrite content (re-dimensionalised to wt % FeS_2); (**c**, **f**, **i**) difference between sulphate and pyrite isotopic composition, Δ_{pyrite} (Eq. S211). Top-row panels are for high-OC conditions ($\Gamma_0 = 50$), whereas middle-row panels are for the global OC average ($\Gamma_0 = 5$) and bottom-row panels are for low-OC conditions ($\Gamma_0 = 0.5$). (**j**) Illustrative non-dimensional content/concentration and isotopic profiles corresponding to three diagnostic regions (marked as coloured circles in each heat map). Shaded areas represent the expected range of profile shapes, each bounded by high-OC (solid lines; darker coloured shading) and low-OC (dotted lines; lighter coloured shading) solutions. In general, Fe_{HR} content is a strong driver of pyrite content but has little impact on pyrite isotopic composition.

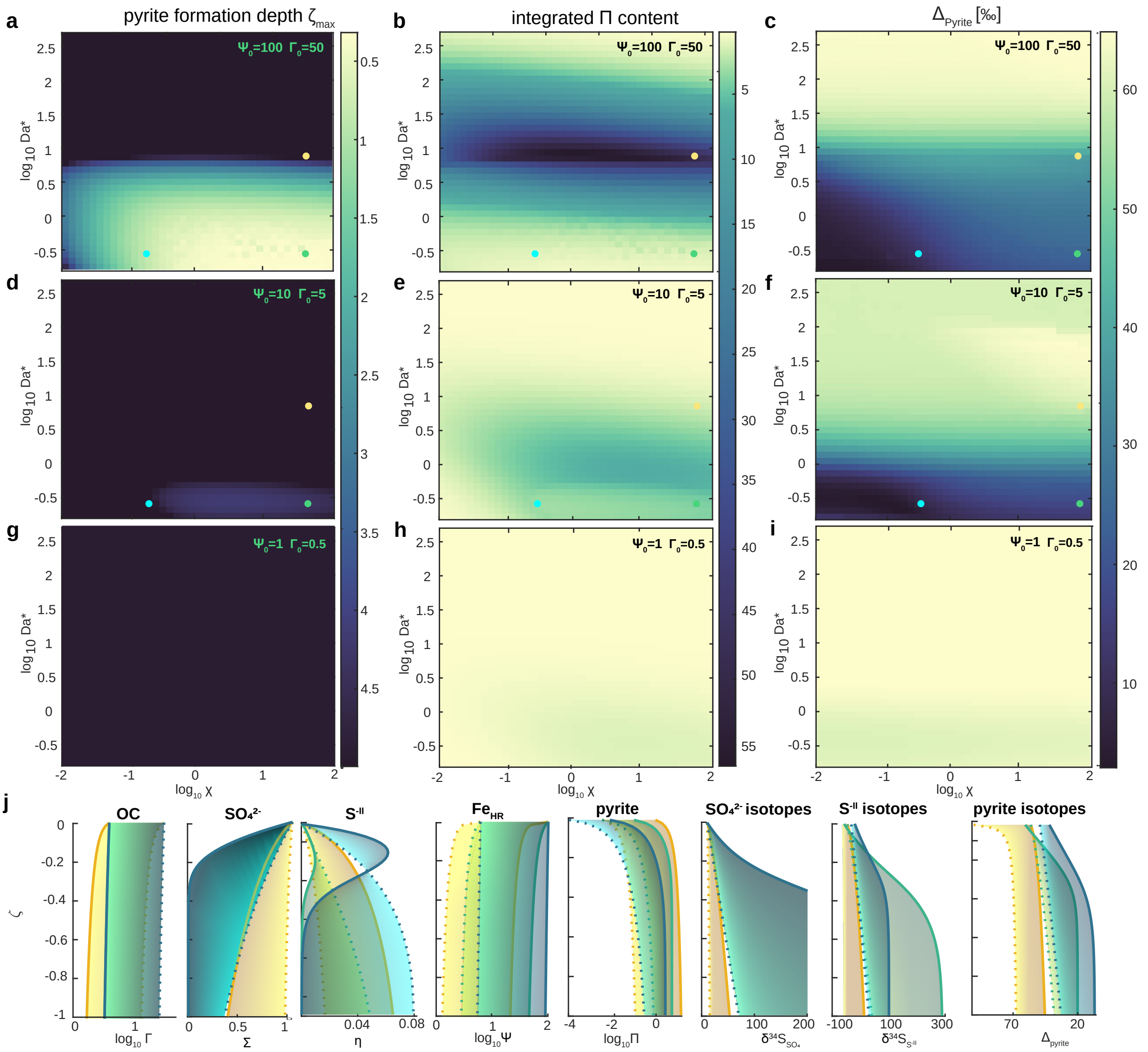

Supplementary Fig. 30 |: Assessing the importance of reactive iron reactivity, χ. Parameter-space heat maps showing how pyrite parameters vary as functions of χ and Da*: (**a**, **d**, **g**) pyrite formation depth, $\zeta_{\max}$ (assuming a threshold value of $\beta = 5 \times 10^{-4}$); (**b**, **e**, **h**) pyrite content (re-dimensionalised to wt % FeS_2); (**c**, **f**, **i**) difference between sulphate and pyrite isotopic composition, Δ_{pyrite} (Eq. S211). Top-row panels are for high-Fe_{HR} and high-OC conditions (Ψ_0 = 100, Γ_0 = 50), whereas middle-row panels reflect global average Fe_{HR} and OC conditions (Ψ_0 = 10, Γ_0 = 5), and bottom-row panels are for low-Fe_{HR} and low-OC conditions (Ψ_0 = 1, Γ_0 = 0.5). (**j**) Illustrative non-dimensional content/concentration and isotopic profiles corresponding to three diagnostic regions (marked as coloured circles in each heat map). Shaded areas represent the expected range of profile shapes, each bounded by high-OC (solid lines; darker coloured shading) and low-OC (dotted lines; lighter coloured shading) solutions. In general, χ is not a strong driver of pyrite formation depth, content, or isotopic composition.

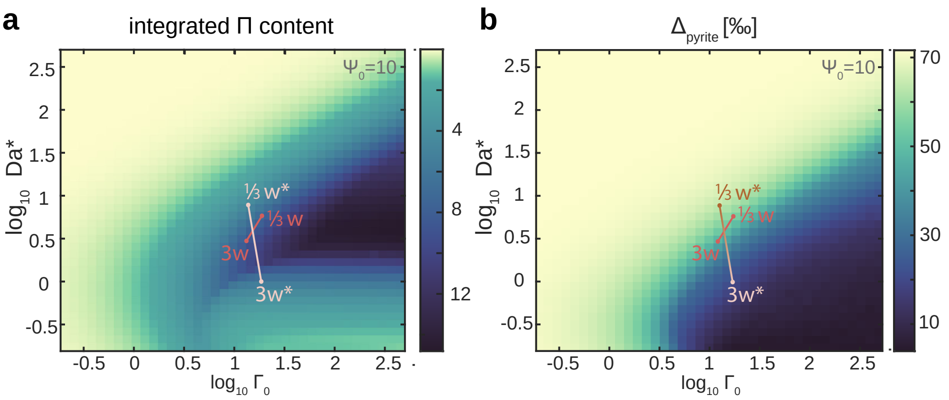

Supplementary Fig. 31 |: Visualising the impact of sedimentation-rate dependencies. Parameter-space heat maps showing how pyrite (**a**) content and (**b**) isotopic offset from sulphate, Δ_{pyrite}, vary as functions of Γ_0 and Da* for the global average Fe_{HR} case (heat maps identical to those shown in Fig. 3). Superimposed on top of each heat map are vectors showing the effect of increasing/decreasing sedimentation rate by a factor of three when including (w) or excluding (w^*) adjustments to sedimentation-rate dependent boundary conditions. Starting point for all vectors represents the average Γ_0 and Da* for shelf and slope environments (i.e., $z_{\text{sw}} \leq 2000$ m). Including dependencies—particularly $k_{G_{\text{sw}}}$ in the definition of Da*—clearly dampens pyrite content and, especially, Δ_{pyrite} sensitivity to changing sedimentation rate.

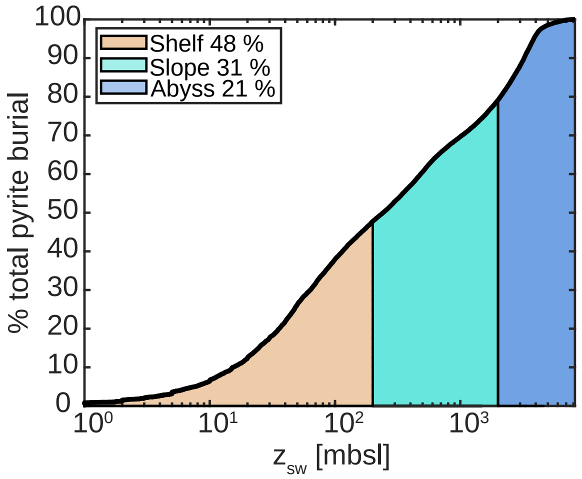

Supplementary Fig. 32 |: Pyrite burial as a function of water depth. Cumulative distribution function (CDF) of pyrite burial flux with overlying water depth. Burial flux is calculated for each grid point as shown in Fig. 2. CDF is colour-coded by regions of continental shelf ($z_{\mathrm{sw}} \leq 200$ m; ~48 % of total burial flux), slope ($200 < z_{\mathrm{sw}} \leq 2000$ m; ~31 % of total burial flux), and abyssal ocean ($z_{\mathrm{sw}} > 2000$ m; ~21 % of total burial flux). Despite contributing only ~10-15 % of total ocean area, the vast majority of pyrite burial occurs in continental shelf and slope regions.

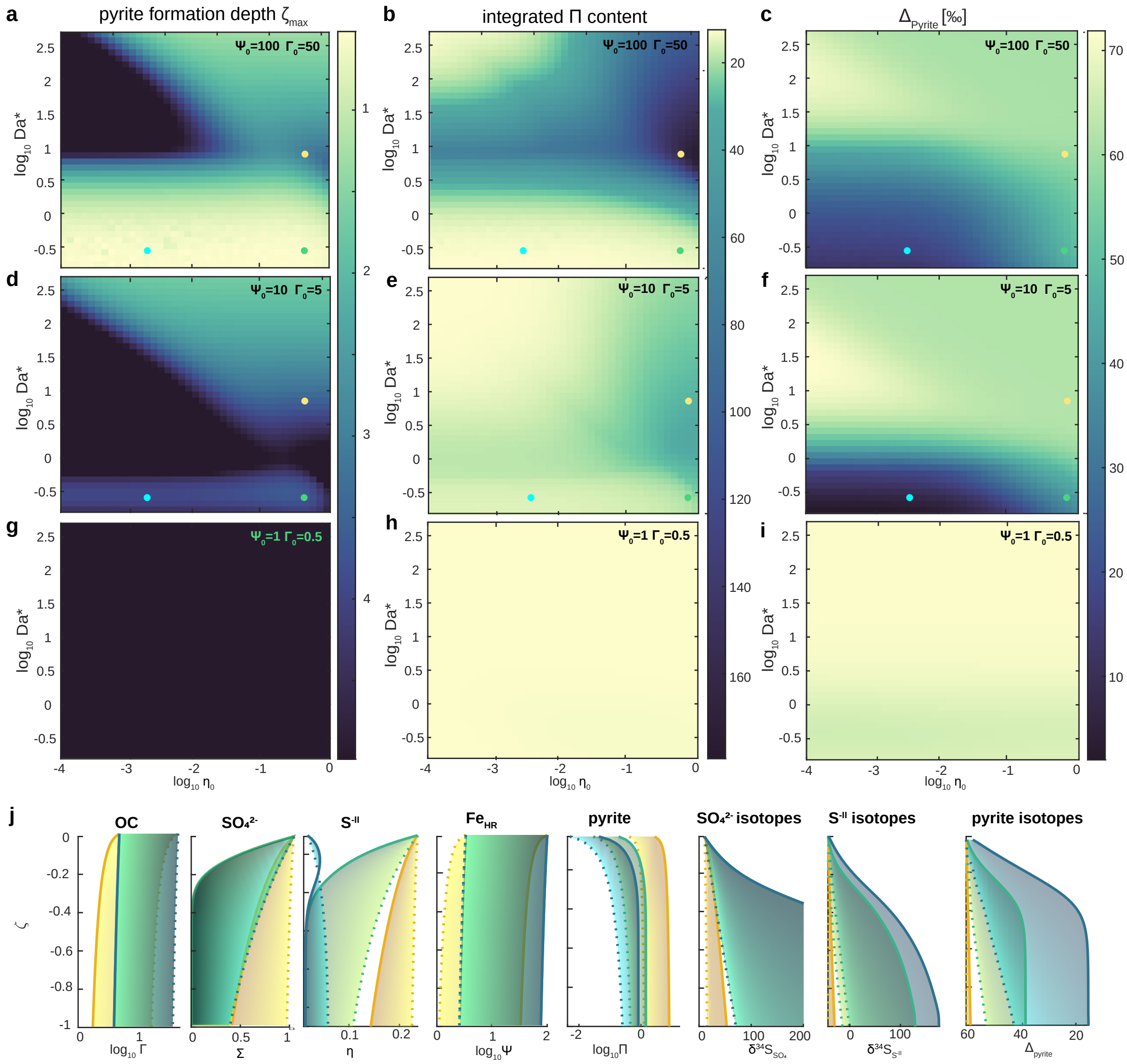

Supplementary Fig. 33 |: Assessing the importance of bottom-water euxinia. Parameter-space heat maps showing how pyrite parameters vary as functions of bottom-water sulphide concentration, η_0, and Da*: (**a**, **d**, **g**) pyrite formation depth, ζ_{max} (assuming a threshold value of $\beta = 5 \times 10^{-4}$); (**b**, **e**, **h**) pyrite content (re-dimensionalised to wt % FeS_2); (**c**, **f**, **i**) difference between sulphate and pyrite isotopic composition, Δ_{pyrite} (Eq. S211). Top-row panels are for high-Fe_{HR} and high-OC conditions ($\Psi_0 = 100$, $\Gamma_0 = 50$), whereas middle-row panels reflect global average Fe_{HR} and OC ($\Psi_0 = 10$, $\Gamma_0 = 5$), and bottom-row panels are for low-Fe_{HR} and low-OC conditions ($\Psi_0 = 1$, $\Gamma_0 = 0.5$). (**j**) Illustrative non-dimensional content/concentration and isotopic profiles corresponding to three diagnostic regions (marked as coloured circles in each heat map). Shaded areas represent the expected range of profile shapes, each bounded by high-OC (solid lines; darker coloured shading) and low-OC (dotted lines; lighter coloured shading) solutions. In general, η_0 only becomes a strong driver of pyrite concentration and isotopic composition for values of $\eta_0 > 0.1$, significantly higher than that observed in any modern setting. For panels (c) and (f), we choose a bottom-water sulphide isotopic composition of −40 ‰ VCDT to reflect modern Black Sea observations[162].

Supplementary References

[108] Aller, R.: Benthic fauna and biogeochemical processes in marine sediments: the role of burrow structures, pp. 301–338. John Wiley & Sons, Hoboken, NJ, USA (1988)

[109] Michaud, E., Robert, C.A., Stora, G.: Sedimentary organic matter distributions, burrowing activity, and biogeochemical cycling: natural patterns and experimental artifacts. Estuarine, Coastal and Shelf Science **90**, 21–34 (2010)

[110] Aller, R.C., Aller, J.Y., Zhu, Q., Heilbrun, C., Klingensmith, I., Kaushik, A.: Worm tubes as conduits for the electrogenic microbial grid in marine sediments. Science Advances **5**, 3651 (2019)

[111] Poulton, S.W., Krom, M.D., Raiswell, R.: A revised scheme for the reactivity of iron (oxyhydr) oxide minerals towards dissolved sulfide. Geochimica et Cosmochimica Acta **68**, 3703–3715 (2004)

[112] Raiswell, R., Berner, R.A.: Pyrite formation in euxinic and semi-euxinic sediments. American Journal of Science **285**, 710–724 (1985)

[113] Lord III, C.J., Church, T.M.: The geochemistry of salt marshes: sedimentary ion diffusion, sulfate reduction, and pyritization. Geochimica et Cosmochimica Acta **47**, 1381–1391 (1983)

[114] Thamdrup, B., Fossing, H., Jørgensen, B.B.: Manganese, iron and sulfur cycling in a coastal marine sediment, Aarhus Bay, Denmark. Geochimica et Cosmochimica Acta **58**, 5115–5129 (1994)

[115] King, G.M.: Effects of added manganic and ferric oxides on sulfate reduction and sulfide oxidation in intertidal sediments. FEMS Microbiology Ecology **6**, 131–138 (1990)

[116] Aller, R.C., Rude, P.D.: Complete oxidation of solid phase sulfides by manganese and bacteria in anoxic marine sediments. Geochimica et Cosmochimica Acta **52**, 751–765 (1988)

[117] Elsgaard, L., Jørgensen, B.B.: Anoxie transformations of radiolabeled hydrogen sulfide in marine and freshwater sediments. Geochimica et Cosmochimica Acta **56**, 2425–2435 (1992)

[118] Raven, M., Keil, R., Webb, S.: Microbial sulfate reduction and organic sulfur formation in sinking marine particles. Science **371**, 178–181 (2021)

[119] Werne, J.P., Hollander, D.J., Lyons, T.W., Damsté, J.S.: Organic sulfur biogeochemistry: recent advances and future research directions. Geological Society of America Special Papers **379**, 135–150 (2004)

[120] Pellerin, A., Bui, T.H., Rough, M., Mucci, A., Canfield, D.E., Wing, B.A.: Mass-dependent sulfur isotope fractionation during reoxidative sulfur cycling: a case study from Mangrove Lake, Bermuda. Geochimica et Cosmochimica Acta **149**, 152–164 (2015)

[121] Fike, D.A., Bradley, A.S., Rose, C.V.: Rethinking the ancient sulfur cycle. Annual Review of Earth and Planetary Sciences **43**, 593–622 (2015)

[122] Rothman, D.H., Forney, D.C.: Physical model for the decay and preservation of marine organic carbon. Science **316**, 1325–1328 (2007)

[123] Hemingway, J.D., Rothman, D.H., Grant, K.E., Rosengard, S.Z., Eglinton, T.I., Derry, L.A., Galy, V.V.: Mineral protection regulates long-term global preservation of natural organic carbon. Nature **570**, 228–231 (2019)

[124] Rothman, D.H.: Slow closure of Earth's carbon cycle. Proceedings of the National Academy of Sciences **121**, 2310998121 (2024)

[125] Halfon, E.: Regression method in ecotoxicology: a better formulation using the geometric mean functional regression. Environmental Science & Technology **19**, 747–749 (1985)

[126] Tenzer, R., Gladkikh, V.: Assessment of density variations of marine sediments with ocean and

sediment depths. The Scientific World Journal **2014**, 823296 (2014)

[127] Pyzik, A.J., Sommer, S.E.: Sedimentary iron monosulfides: kinetics and mechanism of formation. Geochimica et Cosmochimica Acta **45**, 687–698 (1981)

[128] Mazumdar, A., Peketi, A., Joao, H., Dewangan, P., Borole, D., Kocherla, M.: Sulfidization in a shallow coastal depositional setting: diagenetic and palaeoclimatic implications. Chemical Geology **322**, 68–78 (2012)

[129] Zindorf, M., März, C., Wagner, T., Gulick, S.P., Strauss, H., Benowitz, J., Jaeger, J., Schnetger, B., Childress, L., LeVay, L., *et al.*: Deep sulfate-methane-transition and sediment diagenesis in the Gulf of Alaska (IODP Site U1417). Marine Geology **417**, 105986 (2019)

[130] Rooze, J., Egger, M., Tsandev, I., Slomp, C.P.: Iron-dependent anaerobic oxidation of methane in coastal surface sediments: potential controls and impact. Limnology and Oceanography **61**, 267–282 (2016)

[131] Beal, E.J., House, C.H., Orphan, V.J.: Manganese-and iron-dependent marine methane oxidation. Science **325**, 184–187 (2009)

[132] Faust, J.C., Tessin, A., Fisher, B.J., Zindorf, M., Papadaki, S., Hendry, K.R., Doyle, K.A., März, C.: Millennial scale persistence of organic carbon bound to iron in Arctic marine sediments. Nature Communications **12**, 275 (2021)

[133] Hellige, K., Pollok, K., Larese-Casanova, P., Behrends, T., Peiffer, S.: Pathways of ferrous iron mineral formation upon sulfidation of lepidocrocite surfaces. Geochimica et Cosmochimica Acta **81**, 69–81 (2012)

[134] Lasaga, A., Holland, H.: Mathematical aspects of non-steady-state diagenesis. Geochimica et Cosmochimica Acta **40**, 257–266 (1976)

[135] Jørgensen, B.B.: A theoretical model of the stable sulfur isotope distribution in marine sediments. Geochimica et Cosmochimica Acta **43**, 363–374 (1979)

[136] Ding, Y., Fu, X., Xu, L., Li, J., Ouyang, M., Wu, H.: Scaling analysis of diffusion–reaction process in proton exchange membrane fuel cell with the second Damköhler number. Chemical Engineering Journal **465**, 143011 (2023)

[137] Müller, P.J., Suess, E.: Productivity, sedimentation rate, and sedimentary organic matter in the oceans—i. Organic carbon preservation. Deep Sea Research Part A. Oceanographic Research Papers **26**, 1347–1362 (1979)

[138] NASA Ocean Biology Processing Group: Sea-viewing Wide Field-of-view Sensor (SeaWiFS) Level-2 Ocean Color Data, version R2018.8. NASA Ocean Biology Distributed Active Archive Center. accessed [5 May 2024] (2018). https://doi.org/10.5067/ORBVIEW-2/SEAWIFS/L2/OC/2018

[139] NASA Ocean Biology Processing Group: Distance to the nearest coast. NASA Ocean Biology Distributed Active Archive Center. accessed [5 May 2024] (2009)

[140] Seiter, K., Hensen, C., Schröter, J., Zabel, M.: Organic carbon content in surface sediments—defining regional provinces. Deep Sea Research Part I: Oceanographic Research Papers **51**, 2001–2026 (2004)

[141] Lee, T.R., Wood, W.T., Phrampus, B.J.: A machine learning (kNN) approach to predicting global seafloor total organic carbon. Global Biogeochemical Cycles **33**, 37–46 (2019)

[142] Atwood, T.B., Witt, A., Mayorga, J., Hammill, E., Sala, E.: Global patterns in marine sediment carbon stocks. Frontiers in Marine Science **7**, 165 (2020)

[143] Peketi, A., Mazumdar, A., Joao, H., Patil, D., Usapkar, A., Dewangan, P.: Coupled C–S–Fe geochemistry in a rapidly accumulating marine sedimentary system: diagenetic and depositional

implications. Geochemistry, Geophysics, Geosystems **16**, 2865–2883 (2015)

[144] Wijsman, J.W., Middelburg, J.J., Heip, C.H.: Reactive iron in Black Sea sediments: implications for iron cycling. Marine Geology **172**, 167–180 (2001)

[145] Raiswell, R., Canfield, D.E.: Sources of iron for pyrite formation in marine sediments. American Journal of Science **298**, 219–245 (1998)

[146] Teal, L., Bulling, M.T., Parker, E., Solan, M.: Global patterns of bioturbation intensity and mixed depth of marine soft sediments. Aquatic Biology **2**, 207–218 (2008)

[147] Arndt, S., Jørgensen, B.B., LaRowe, D.E., Middelburg, J., Pancost, R., Regnier, P.: Quantifying the degradation of organic matter in marine sediments: a review and synthesis. Earth-Science Reviews **123**, 53–86 (2013)

[148] Freitas, F.S., Pika, P.A., Kasten, S., Jørgensen, B.B., Rassmann, J., Rabouille, C., Thomas, S., Sass, H., Pancost, R.D., Arndt, S.: New insights into large-scale trends of apparent organic matter reactivity in marine sediments and patterns of benthic carbon transformation. Biogeosciences **18**, 4651–4679 (2021)

[149] Pika, P.A., Hülse, D., Eglinton, T.I., Arndt, S.: Regional and global patterns of apparent organic matter reactivity in marine sediments. Global Biogeochemical Cycles **505**, 2022–007636 (2023)

[150] Malinverno, A., Martinez, E.A.: The effect of temperature on organic carbon degradation in marine sediments. Scientific Reports **5**, 1–10 (2015)

[151] Stolpovsky, K., Dale, A.W., Wallmann, K.: A new look at the multi-G model for organic carbon degradation in surface marine sediments for coupled benthic–pelagic simulations of the global ocean. Biogeosciences **15**, 3391–3407 (2018)

[152] Reimers, C.E., Suess, E.: The partitioning of organic carbon fluxes and sedimentary organic matter decomposition rates in the ocean. Marine Chemistry **13**, 141–168 (1983)

[153] Müller, P., Mangini, A.: Organic carbon decomposition rates in sediments of the Pacific manganese nodule belt dated by ^{230}Th and ^{231}Pa. Earth and Planetary Science Letters **51**, 94–114 (1980)

[154] Harvey, H.R., Tuttle, J.H., Bell, J.T.: Kinetics of phytoplankton decay during simulated sedimentation: changes in biochemical composition and microbial activity under oxic and anoxic conditions. Geochimica et Cosmochimica Acta **59**, 3367–3377 (1995)

[155] Borowski, W.S., Paull, C.K., Ussler Iii, W.: Global and local variations of interstitial sulfate gradients in deep-water, continental margin sediments: sensitivity to underlying methane and gas hydrates. Marine Geology **159**, 131–154 (1999)

[156] Emerson, S.: Organic carbon preservation in marine sediments, vol. 32, pp. 78–87. American Geophysical Union, Washington, D.C., USA (1985)

[157] Toth, D.J., Lerman, A.: Organic matter reactivity and sedimentation rates in the ocean. Geochimica et Cosmochimica Acta **41**, 1405–1412 (1977)

[158] Stolpovsky, K., Dale, A.W., Wallmann, K.: Toward a parameterization of global-scale organic carbon mineralization kinetics in surface marine sediments. Global Biogeochemical Cycles **29**, 812–829 (2015)

[159] National Geophysical Data Center: The NGDC Seafloor Sediment Grain Size Database. First Version. NOAA National Centers for Environmental Information. Accessed [3 June 2024] (1976). https://doi.org/10.7289/V5G44N6W

[160] Paul, R.K.: Multicollinearity: causes, effects and remedies. IASRI, New Delhi **1**, 58–65 (2006)

[161] Aharon, P., Fu, B.: Sulfur and oxygen isotopes of coeval sulfate–sulfide in pore fluids of cold seep

sediments with sharp redox gradients. Chemical Geology **195**, 201–218 (2003)

[162] Volkov, I.I., Neretin, L.N.: Hydrogen sulfide in the Black Sea, pp. 309–331. Springer, Berlin, Germany (2008)

[163] Canfield, D.E., Berner, R.A.: Dissolution and pyritization of magnetite in anoxie marine sediments. Geochimica et Cosmochimica Acta **51**, 645–659 (1987)

[164] Böttcher, M.E., Ferdelman, T.G., Jørgensen, B.B., Blake, R., Surkov, A.V., Claypool, G.E., *et al.*: Sulfur isotope fractionation by the deep biosphere within sediments of the eastern equatorial Pacific and Peru Margin. In: Proceedings of the Ocean Drilling Program, Scientific Results, vol. 201, pp. 1–21 (2006)

[165] Michaud, A.B., Laufer, K., Findlay, A., Pellerin, A., Antler, G., Turchyn, A.V., Røy, H., Wehrmann, L.M., Jørgensen, B.B.: Glacial influence on the iron and sulfur cycles in Arctic fjord sediments (Svalbard). Geochimica et Cosmochimica Acta **280**, 423–440 (2020)

[166] Borowski, W.S., Rodriguez, N.M., Paull, C.K., Ussler III, W.: Are ^{34}S-enriched authigenic sulfide minerals a proxy for elevated methane flux and gas hydrates in the geologic record? Marine and Petroleum Geology **43**, 381–395 (2013)

[167] Mossmann, J.-R., Aplin, A.C., Curtis, C.D., Coleman, M.L.: Geochemistry of inorganic and organic sulphur in organic-rich sediments from the Peru Margin. Geochimica et Cosmochimica Acta **55**, 3581–3595 (1991)

[168] Brüchert, V., Pratt, L.M.: Stable sulfur isotopic evidence for historical changes of sulfur cycling in estuarine sediments from northern Florida. Aquatic Geochemistry **5**, 249–268 (1999)

[169] Neretin, L.N., Böttcher, M.E., Jørgensen, B.B., Volkov, I.I., Lüschen, H., Hilgenfeldt, K.: Pyritization processes and greigite formation in the advancing sulfidization front in the Upper Pleistocene sediments of the Black Sea. Geochimica et Cosmochimica Acta **68**, 2081–2093 (2004)

[170] Brüchert, V., Knoblauch, C., Jørgensen, B.B.: Controls on stable sulfur isotope fractionation during bacterial sulfate reduction in Arctic sediments. Geochimica et Cosmochimica Acta **65**, 763–776 (2001)

[171] Calvert, S., Thode, H., Yeung, D., Karlin, R.: A stable isotope study of pyrite formation in the Late Pleistocene and Holocene sediments of the Black Sea. Geochimica et Cosmochimica Acta **60**, 1261–1270 (1996)

[172] Crémière, A., Strauss, H., Sebilo, M., Hong, W.-L., Gros, O., Schmidt, S., Tocny, J., Henry, F., Gontharet, S., Laverman, A.M.: Sulfur diagenesis under rapid accumulation of organic-rich sediments in a marine mangrove from Guadeloupe (French West Indies). Chemical Geology **454**, 67–79 (2017)

[173] Riedinger, N., Brunner, B.: Data report: concentration and sulfur isotope composition of iron monosulfide and pyrite from sediments collected during IODP Expedition 316. In: Proceedings of the Ocean Drilling Program, Scientific Results, vol. 314, pp. 315–316 (2014)

[174] Dale, A.W., Brüchert, V., Alperin, M., Regnier, P.: An integrated sulfur isotope model for Namibian shelf sediments. Geochimica et Cosmochimica Acta **73**, 1924–1944 (2009)

[175] Diaz, R., Moreira, M., Mendoza, U., Machado, W., Böttcher, M.E., Santos, H., Belém, A., Capilla, R., Escher, P., Albuquerque, A.L.: Early diagenesis of sulfur in a tropical upwelling system, Cabo Frio, southeastern Brazil. Geology **40**, 879–882 (2012)

[176] Antler, G., Turchyn, A.V., Rennie, V., Herut, B., Sivan, O.: Coupled sulfur and oxygen isotope insight into bacterial sulfate reduction in the natural environment. Geochimica et Cosmochimica Acta **118**, 98–117 (2013)

[177] Riedinger, N., Brunner, B., Krastel, S., Arnold, G.L., Wehrmann, L.M., Formolo, M.J., Beck, A.,

Bates, S.M., Henkel, S., Kasten, S., *et al.*: Sulfur cycling in an iron oxide-dominated, dynamic marine depositional system: the Argentine continental margin. Frontiers in Earth Science **5**, 33 (2017)

[178] Sweeney, R.E., Kaplan, I.: Diagenetic sulfate reduction in marine sediments. Marine Chemistry **9**, 165–174 (1980)

[179] Donahue, M.A., Werne, J.P., Meile, C., Lyons, T.W.: Modeling sulfur isotope fractionation and differential diffusion during sulfate reduction in sediments of the Cariaco Basin. Geochimica et Cosmochimica Acta **72**, 2287–2297 (2008)

[180] Hardisty, D.S., Lyons, T.W., Riedinger, N., Isson, T.T., Owens, J.D., Aller, R.C., Rye, D.M., Planavsky, N.J., Reinhard, C.T., Gill, B.C., *et al.*: An evaluation of sedimentary molybdenum and iron as proxies for pore fluid paleoredox conditions. American Journal of Science **318**, 527–556 (2018)

[181] Henneke, E., Luther III, G.W., De Lange, G.J., Hoefs, J.: Sulphur speciation in anoxic hypersaline sediments from the eastern Mediterranean Sea. Geochimica et Cosmochimica Acta **61**, 307–321 (1997)

[182] Böning, P., Brumsack, H.-J., Böttcher, M.E., Schnetger, B., Kriete, C., Kallmeyer, J., Borchers, S.L.: Geochemistry of Peruvian near-surface sediments. Geochimica et Cosmochimica Acta **68**, 4429–4451 (2004)

[183] Hu, Y., Chen, L., Feng, D., Liang, Q., Xia, Z., Chen, D.: Geochemical record of methane seepage in authigenic carbonates and surrounding host sediments: a case study from the South China Sea. Journal of Asian Earth Sciences **138**, 51–61 (2017)

[184] Turchyn, A.V., Antler, G., Byrne, D., Miller, M., Hodell, D.A.: Microbial sulfur metabolism evidenced from pore fluid isotope geochemistry at Site U1385. Global and Planetary Change **141**, 82–90 (2016)

[185] Wehrmann, L.M., Formolo, M.J., Owens, J.D., Raiswell, R., Ferdelman, T.G., Riedinger, N., Lyons, T.W.: Iron and manganese speciation and cycling in glacially influenced high-latitude fjord sediments (West Spitsbergen, Svalbard): evidence for a benthic recycling-transport mechanism. Geochimica et Cosmochimica Acta **141**, 628–655 (2014)

[186] Jørgensen, B.B., Böttcher, M.E., Lüschen, H., Neretin, L.N., Volkov, I.I.: Anaerobic methane oxidation and a deep H_2S sink generate isotopically heavy sulfides in Black Sea sediments. Geochimica et Cosmochimica Acta **68**, 2095–2118 (2004)

[187] Böttcher, M.E., Brumsack, H.-J., De Lange, G.J., Robertson, A.: Sulfate reduction and related stable isotope (δ^{34}S, δ^{18}O) variations in interstitial waters from the Eastern Mediterranean. In: Proceedings of the Ocean Drilling Program, Scientific Results, vol. 160, pp. 365–376 (1998)

[188] Ku, T., Walter, L., Coleman, M., Blake, R., Martini, A.M.: Coupling between sulfur recycling and syndepositional carbonate dissolution: evidence from oxygen and sulfur isotope composition of pore water sulfate, South Florida Platform, USA. Geochimica et Cosmochimica Acta **63**, 2529–2546 (1999)

[189] Ku, T.C., Kay, J., Browne, E., Martini, A.M., Peters, S.C., Chen, M.D.: Pyritization of iron in tropical coastal sediments: implications for the development of iron, sulfur, and carbon diagenetic properties, Saint Lucia, Lesser Antilles. Marine Geology **249**, 184–205 (2008)

[190] Wehrmann, L.M., Titschack, J., Böttcher, M.E., Ferdelman, T.G.: Linking sedimentary sulfur and iron biogeochemistry to growth patterns of a cold-water coral mound in the Porcupine Basin, SW Ireland (IODP Expedition 307). Geobiology **13**, 424–442 (2015)

[191] Wehrmann, L.M., Riedinger, N., Brunner, B., Kamyshny Jr, A., Hubert, C.R., Herbert, L.C., Brüchert, V., Jørgensen, B.B., Ferdelman, T.G., Formolo, M.J.: Iron-controlled oxidative sulfur cycling recorded in the distribution and isotopic composition of sulfur species in glacially influenced

fjord sediments of west Svalbard. Chemical Geology **466**, 678–695 (2017)

[192] Lein, A.Y.: Biogeochemistry of the anaerobic diagenesis of recent Baltic Sea sediments. Ecological Bulletins, 441–461 (1983)

[193] Werne, J.P., Lyons, T.W., Hollander, D.J., Formolo, M.J., Damsté, J.S.S.: Reduced sulfur in euxinic sediments of the Cariaco Basin: sulfur isotope constraints on organic sulfur formation. Chemical Geology **195**, 159–179 (2003)

[194] Böttcher, M.E., Bernasconi, S.M., Brumsack, H.-J.: 32. Carbon, sulfur, and oxygen isotope geochemistry of interstitial waters from the western Mediterranean. In: Proceedings of the Ocean Drilling Program, Scientific Results, vol. 161, pp. 413–421 (1999)

[195] Lein, A.Y., Ivanov, M.V.: Biogeokhimicheskii Tsikl Metana V Okeane (Biogeochemical Cycle of Methane in the Ocean)., pp. 1–576. Nauka Publishers, Moscow, Russia (2009)

[196] Lin, Z., Sun, X., Lu, Y., Xu, L., Gong, J., Lu, H., Teichert, B.M., Peckmann, J.: Stable isotope patterns of coexisting pyrite and gypsum indicating variable methane flow at a seep site of the Shenhu area, South China Sea. Journal of Asian Earth Sciences **123**, 213–223 (2016)

[197] Lin, Q., Wang, J., Taladay, K., Lu, H., Hu, G., Sun, F., Lin, R.: Coupled pyrite concentration and sulfur isotopic insight into the paleo sulfate–methane transition zone (SMTZ) in the northern South China Sea. Journal of Asian Earth Sciences **115**, 547–556 (2016)

[198] Böttcher, M.E., Hespenheide, B., Llobet-Brossa, E., Beardsley, C., Larsen, O., Schramm, A., Wieland, A., Böttcher, G., Berninger, U.-G., Amann, R.: The biogeochemistry, stable isotope geochemistry, and microbial community structure of a temperate intertidal mudflat: an integrated study. Continental Shelf Research **20**, 1749–1769 (2000)

[199] Lin, Z., Sun, X., Peckmann, J., Lu, Y., Xu, L., Strauss, H., Zhou, H., Gong, J., Lu, H., Teichert, B.M.: How sulfate-driven anaerobic oxidation of methane affects the sulfur isotopic composition of pyrite: a SIMS study from the South China Sea. Chemical Geology **440**, 26–41 (2016)

[200] Böttcher, M.E., Khim, B.-K., Suzuki, A.: 7. Microbial sulfate reduction in interstitial waters from sediments of the Southwest Pacific (Sites 1119–1124): evidence from stable sulfur isotopes. In: Proceedings of the Ocean Drilling Program, Scientific Results, vol. 181, pp. 1–11 (2002)

[201] Lin, Q., Wang, J., Algeo, T.J., Sun, F., Lin, R.: Enhanced framboidal pyrite formation related to anaerobic oxidation of methane in the sulfate-methane transition zone of the northern South China Sea. Marine Geology **379**, 100–108 (2016)

[202] Yücel, M., Moore, W.S., Butler, I.B., Boyce, A., Luther III, G.W.: Recent sedimentation in the Black Sea: new insights from radionuclide distributions and sulfur isotopes. Deep Sea Research Part I: Oceanographic Research Papers **66**, 103–113 (2012)

[203] Lin, Z., Sun, X., Strauss, H., Lu, Y., Gong, J., Xu, L., Lu, H., Teichert, B.M., Peckmann, J.: Multiple sulfur isotope constraints on sulfate-driven anaerobic oxidation of methane: evidence from authigenic pyrite in seepage areas of the South China Sea. Geochimica et Cosmochimica Acta **211**, 153–173 (2017)

[204] Lin, Z., Sun, X., Strauss, H., Lu, Y., Böttcher, M.E., Teichert, B.M., Gong, J., Xu, L., Liang, J., Lu, H., Chen, H., Wang, X., Wu, N., Yao, Y., Zhang, F., Qiu, W., Liu, Y., Zheng, M.: Multiple sulfur isotopic evidence for the origin of elemental sulfur in an iron-dominated gas hydrate-bearing sedimentary environment. Marine Geology **403**, 271–284 (2018)

[205] Böttcher, M.E., Schale, H., Schnetger, B., Wallmann, K., Brumsack, H.-J.: Stable sulfur isotopes indicate net sulfate reduction in near-surface sediments of the deep Arabian Sea. Deep Sea Research Part II: Topical Studies in Oceanography **47**, 2769–2783 (2000)

[206] Liu, J., Pellerin, A., Izon, G., Wang, J., Antler, G., Liang, J., Su, P., Jørgensen, B.B., Ono, S.: The

multiple sulphur isotope fingerprint of a sub-seafloor oxidative sulphur cycle driven by iron. Earth and Planetary Science Letters **536**, 116165 (2020)

[207] Zhu, M.-X., Chen, K.-K., Yang, G.-P., Fan, D.-J., Li, T.: Sulfur and iron diagenesis in temperate unsteady sediments of the east china sea inner shelf and a comparison with tropical mobile mud belts (MMBs). Journal of Geophysical Research: Biogeosciences **121**, 2811–2828 (2016)

[208] Liu, J., Pellerin, A., Antler, G., Kasten, S., Findlay, A.J., Dohrmann, I., Røy, H., Turchyn, A.V., Jørgensen, B.B.: Early diagenesis of iron and sulfur in Bornholm Basin sediments: the role of near-surface pyrite formation. Geochimica et Cosmochimica Acta **284**, 43–60 (2020)

[209] Zopfi, J., Böttcher, M.E., Jørgensen, B.B.: Biogeochemistry of sulfur and iron in *thioploca*-colonized surface sediments in the upwelling area off central Chile. Geochimica et Cosmochimica Acta **72**, 827–843 (2008)

[210] Llobet-Brossa, E., Rabus, R., Böttcher, M.E., Könneke, M., Finke, N., Schramm, A., Meyer, R.L., Grötzschel, S., Rosselló-Mora, R., Amann, R.: Community structure and activity of sulfate-reducing bacteria in an intertidal surface sediment: a multi-method approach. Aquatic Microbial Ecology **29**, 211–226 (2002)

[211] Masterson, A., Alperin, M.J., Berelson, W.M., Johnston, D.T.: Interpreting multiple sulfur isotope signals in modern anoxic sediments using a full diagenetic model (California-Mexico margin: Alfonso basin). American Journal of Science **318**, 459–490 (2018)

[212] Böttcher, M.E., Khim, B.-K., Suzuki, A., Gehre, M., Wortmann, U.G., Brumsack, H.-J.: Microbial sulfate reduction in deep sediments of the Southwest Pacific (ODP Leg 181, Sites 1119–1125): evidence from stable sulfur isotope fractionation and pore water modeling. Marine Geology **205**, 249–260 (2004)

[213] Meister, P., Brunner, B., Picard, A., Böttcher, M.E., Jørgensen, B.B.: Sulphur and carbon isotopes as tracers of past sub-seafloor microbial activity. Scientific Reports **9**, 604 (2019)
